# Excited-state antiaromaticity relief drives facile photoprotonation of carbons in aminobiphenyls†

**DOI:** 10.1039/d4sc00642a

**Published:** 2024-03-04

**Authors:** Josip Draženović, Croix J. Laconsay, Nađa Došlić, Judy I-Chia Wu, Nikola Basarić

**Affiliations:** a Department of Organic Chemistry and Biochemistry, Ruđer Bošković Institute Bijenička Cesta 54 10000 Zagreb Croatia nbasaric@irb.hr; b Department of Chemistry, University of Houston Houston TX 77204 USA jiwu@central.uh.edu; c Department of Physical Chemistry, Ruđer Bošković Institute Bijenička Cesta 54 10000 Zagreb Croatia nadja.doslic@irb.hr

## Abstract

A combined computational and experimental study reveals that *ortho*-, *meta*- and *para*-aminobiphenyl isomers undergo distinctly different photochemical reactions involving proton transfer. Deuterium exchange experiments show that the *ortho*-isomer undergoes a facile photoprotonation at a carbon atom *via* excited-state intramolecular proton transfer (ESIPT). The *meta*-isomer undergoes water-assisted excited-state proton transfer (ESPT) and a photoredox reaction *via* proton-coupled electron transfer (PCET). The *para*-isomer undergoes a water-assisted ESPT reaction. All three reactions take place in the singlet excited-state, except for the photoredox process of the *meta*-isomer, which involves a triplet excited-state. Computations illustrate the important role of excited-state antiaromaticity relief in these photoreactions.

## Abstract 2

*ortho*-, *meta*- and *para*-aminobiphenyl isomers undergo distinctly different photochemical reactions involving proton transfer, which are driven by excited-state antiaromaticity relief.

## Graphical abstract

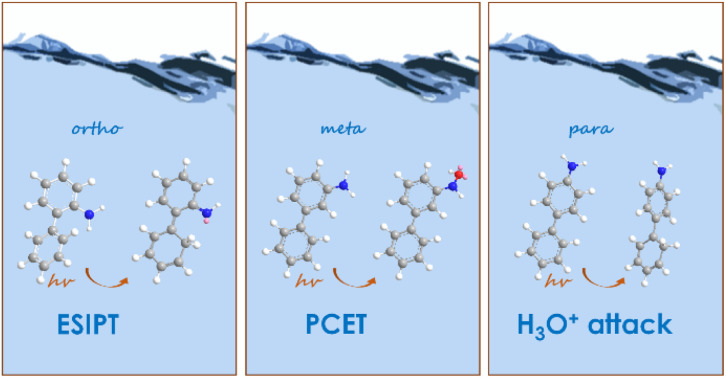

## Introduction

Excited-state proton transfer reactions typically occur between a proton donor (*e.g.*, NH or OH) and an electronegative heteroatom.^1^ Sometimes, however, the proton acceptor can be a carbon atom.^2^ Examples of facile photoprotonation in aromatic organic compounds by aqueous acids have been known since the mid-1980's.^3–5^ In 2002, Wan *et al.* reported the first example of direct photoprotonation of a carbon atom of an aromatic ring *via* excited-state intramolecular proton transfer (ESIPT).^6,7^ Irradiating 2-phenylphenol in D_2_O–CH_3_CN solution led to regioselective D-exchange at the 2′ and 4′ positions. Deuteration at the 2′ position was believed to proceed through ESIPT involving an initial hydrogen bonding interaction between the OH group of the phenol moiety and the π-system of the adjacent phenyl moiety. Upon irradiation, the phenolic OH proton shifts to the 2′ position of the unsubstituted phenyl ring. When the distance between the acidic and basic carbon site is far, regioselective D-exchange can occur by excited-state proton transfer (ESPT) involving a chain of protic solvents.^8–14^ Many examples of ESIPT and ESPT involving proton transfer, either from an OH group^15–17^ or an amine group^18–20^ to a basic carbon atom have been reported. These findings are at first sight somewhat surprising, since protonation of a carbon atom of an aromatic ring typically is a slow process in the ground state.^21,22^ Yet, why do some aromatic ring carbons gain basicity in the excited-state?

**Scheme 1:**
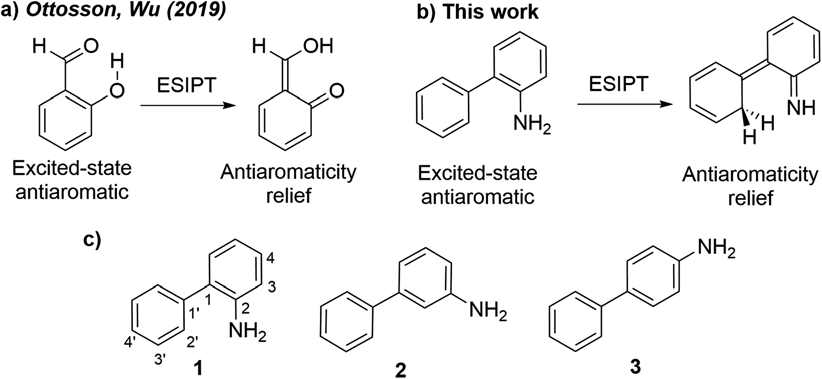
(a) Antiaromaticity relief in the ESIPT reaction of *o*-salicylic acid.^23^ (b) Antiaromaticity relief in the ESIPT reaction of *o*-aminobiphenyl. (c) Amino arene compounds investigated and carbon numberings for the phenyl and aniline rings.

Some of us recently related the phenomena of excited-state proton transfer in aromatic organic compounds to a reversal of aromaticity and antiaromaticity in the lowest ππ* excited-states.^23^*o*-Salicylic acid,^24^ for example, is 4*n* + 2 aromatic in the ground state, but shows enhanced antiaromatic character in the first ^1^ππ* state. Proton transfer from an OH group to the carbonyl site disrupts cyclic 4*n* + 2 conjugation in the six membered ring and this reaction alleviates excited-state antiaromaticity (Scheme 1a). The low barriers of many other excited state proton and electron transfer reactions, for example, ESIPT in phenol-benzoxazoles,^25^ the photoacidity of naphthols,^26^ and proton coupled electron transfer reactions in phenols^27^ have been attributed to the effects of excited-state antiaromaticity relief. The concepts of excited-state aromaticity and antiaromaticity were first explored by Dewar,^28^ Zimmerman,^29–31^ and later Dougherty^32^ to explain the mechanisms of pericyclic reactions. Later, Baird proposed, based on a set of perturbation molecular orbital theory analyses, that “the rules for ground state aromaticity are reversed in the first ^3^ππ* state: 4*n* rings display ‘aromatic’ character whereas 4*n* + 2 systems display ‘antiaromaticity’.^33^ Baird's rules apply also to the lowest ππ* singlet excited states of annulenes^34–36^ and has garnered considerable attention in many areas of applied organic photochemistry.^37–40^

Here, we relate the potential for carbon atom photobasicity in aromatic ring to a reversal of aromaticity and antiaromaticity in the lowest ππ* excited states. In the ground state, protonating an aromatic ring and disrupting cyclic 4*n* + 2 π-conjugation is a slow process due to a loss of aromaticity. But in the lowest singlet or triplet excited state, these rings gain antiaromatic character and the ring carbon atoms display increased basicity, as protonation now affords a channel to alleviate excited-state antiaromaticity (Scheme 1b). To model the effects of excited-state antiaromaticity relief in the photoprotonation of aromatic ring carbons, here, we investigate the photophysics and photochemistry of a series of *ortho*-(1), *meta*-(2) and *para*-(3) aminobiphenyls (Scheme 1c). These chromophores can be found in many structures with important applications in supramolecular chemistry or material science.^41^ We now report a combined experimental and computational study, showing that the aromatic rings of aminobiphenyls can gain significant antiaromatic character in the singlet and/or triplet states, demonstrating that antiaromaticity relief is a major driving force for the photoreactivities of *ortho*-(1), *meta*-(2) and *para*-(3) aminobiphenyls.

## Results and discussion

### Photophysical properties

**Fig. 1:**
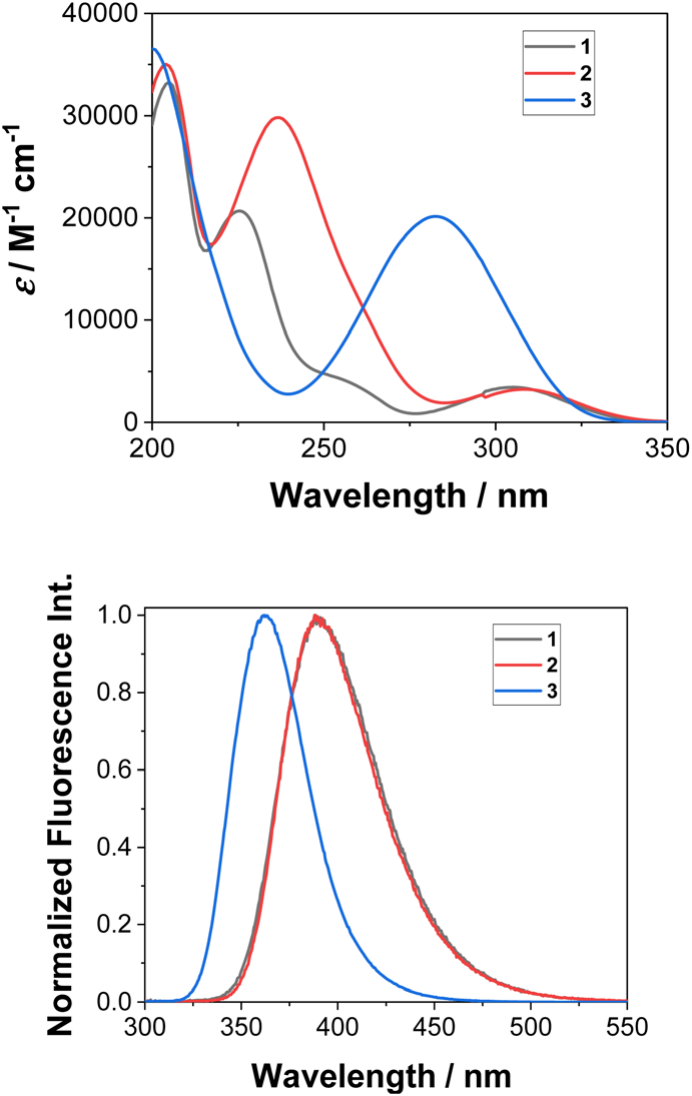
Measured absorption (top) and normalized emission spectra (bottom) of 1–3 in CH_3_CN (*λ*_exc_ = 280 nm).

Absorption and emission spectra for the aminobiphenyls 1–3 were recorded in CH_3_CN (see Fig. S1–S3 in the ESI†). The absorption spectra of 1 and 2 show maxima at 305 nm, and that of 3 shows a maximum at 283 nm (Fig. 1, top). These absorption bands correspond to the S_1_ ← S_0_ transition and are characterized as ππ* states as confirmed by the symmetry of the computed orbitals. The higher singlet states of 1 (≈250 nm, shoulder), 2 (236 nm), and 3 (200 nm) indicate significant charge transfer character. The emission spectra of 1 and 2 nearly overlap and show maxima at 390 nm, while that of 3 shows a maximum at 361 nm (Fig. 1, bottom). Stokes' shifts of all three isomers range between 6600–7500 cm^−1^ (Table S1 in the ESI†). Quantum yields of fluorescence were measured by use of *N*-acetyl-tryptophanamide in water as a reference (*Φ*_f_ = 0.12).^42^ In accordance with an observed lower photoreactivity, 3 (*Φ*_f_ = 0.55) shows higher fluorescence compared to 1 (*Φ*_f_ = 0.21) and 2 (*Φ*_f_ = 0.26).

Time-correlated single photon counting (TC-SPC) experiments were performed to measure the decay of fluorescence for 1–3 in CH_3_CN solutions (Fig. S7–S9 and Table S2 in the ESI†). The decay times do not fit to a single-exponential function. In addition to the major decay component (2.5–6.6 ns), a short decay component was detected (<100 ps); however, a precise measurement for the short decay component was not possible with the setup used. Dual fluorescence for aminonaphthalenes have been observed and attributed to emission from the L_a_ and L_b_ states.^43^ Our computations agree with this interpretation, since the excitation at 280 nm used for the SPC can give rise to the adiabatic transition to the S_2_ state. The other, less likely interpretation for dual emission would be that it originates from different vibrational levels of the excited states.^44^

**Fig. 2:**
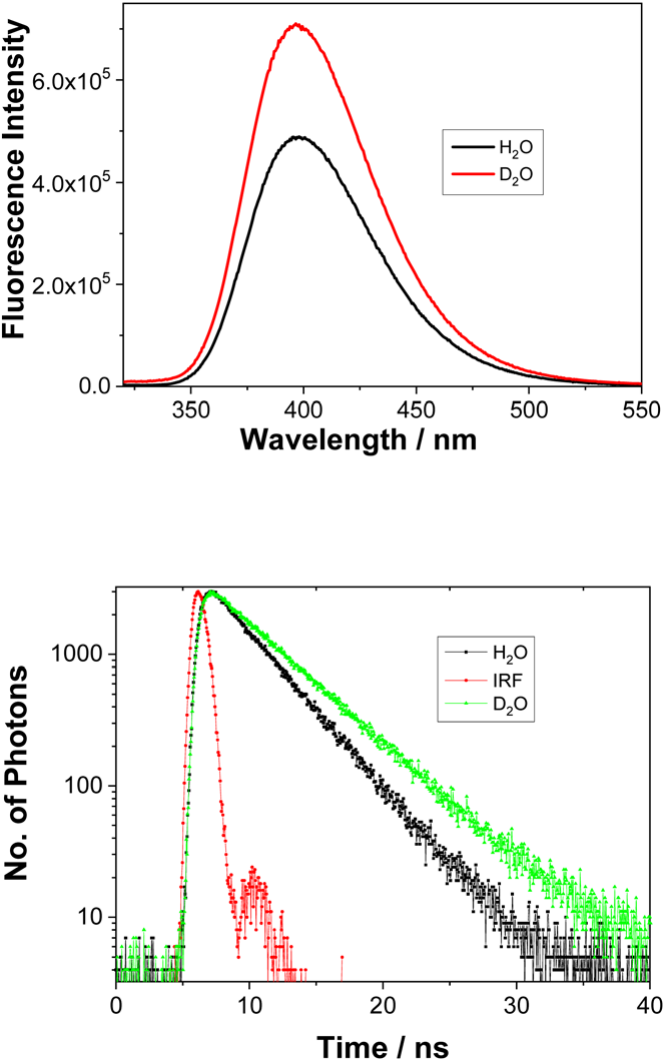
Fluorescence spectra of 1 (*λ*_ex_ = 280 nm) in CH_3_CN–H_2_O (1 : 1) or CH_3_CN–D_2_O (1 : 1) (top) and the corresponding decays of fluorescence at 400 nm (bottom). Fitting data can be found in Table S3.†

Addition of a protic solvent (*i.e.*, H_2_O) to CH_3_CN increases *Φ*_f_ for all three compounds (Fig. S4–S6 in the ESI†). We surmise that the presence of H_2_O may hinder vibrational modes that lead to a non-radiative decay from the S_1_ state, thereby enhancing fluorescence. Direct comparisons of the emission spectra for 1–3 in CH_3_CN–H_2_O and in CH_3_CN–D_2_O, reveal stronger fluorescence and a slower decay in D_2_O (Fig. 2, S10, S11 and Table S3 in the ESI†), especially for 1. This finding strongly indicates the involvement of H_2_O in blocking a non-radiative decay pathway from the singlet excited state; D_2_O shows slower decay kinetics due to a kinetic isotope effect. These findings are in line with the observed ESIPT reactivity of 1, which takes place only in the presence of H_2_O as a protic solvent.

### Acid–base properties

**Fig. 3:**
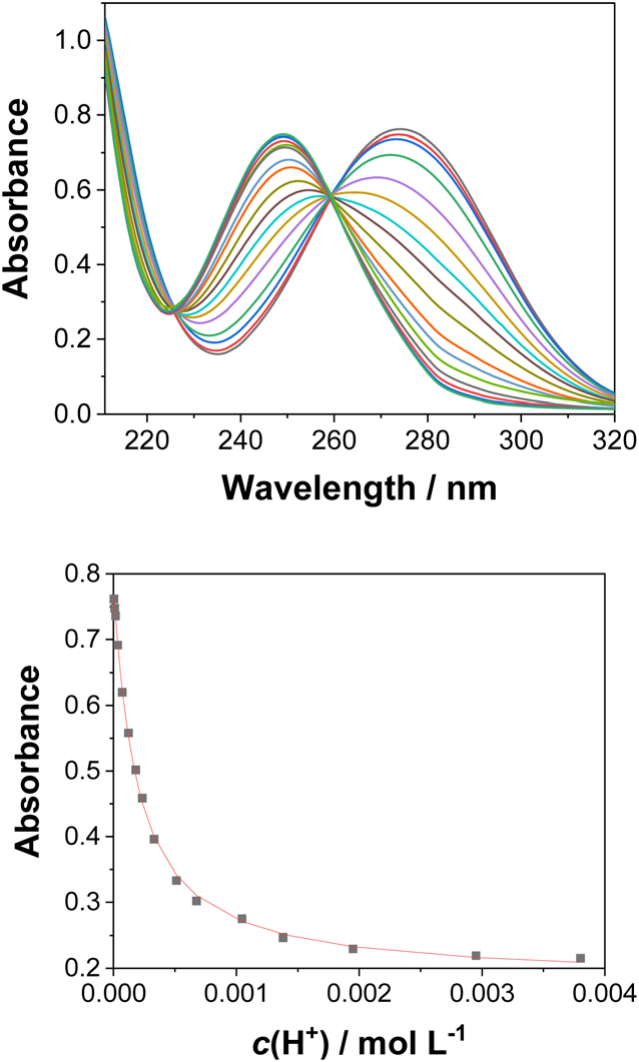
Absorption spectra of 3 (*c*_0_ = 3.67 × 10^−5^ M) in CH_3_CN–H_2_O (1 : 4) at different pH values (top), and dependence of the absorption at 274 nm on the pH (bottom). The black dots are experimental values and the red line corresponds to a model involving a one-step protonation equilibrium.

**Fig. 4:**
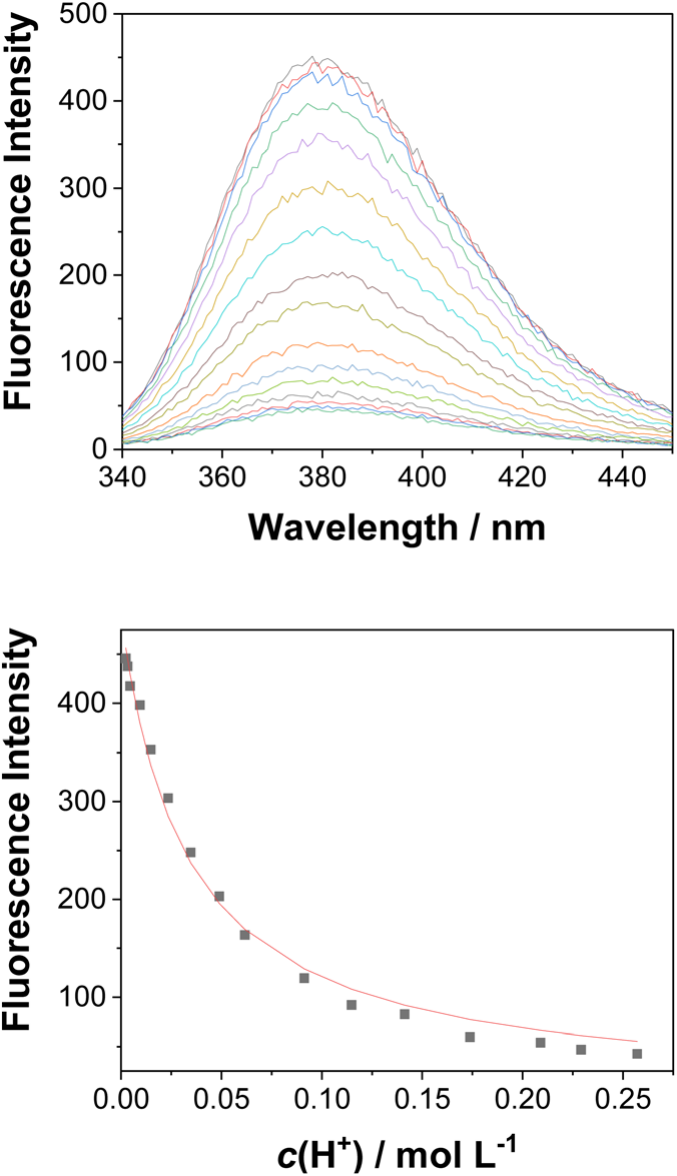
Fluorescence spectra (*λ*_ex_ = 260 nm) of 3 (*c*_0_ = 4.51 × 10^−6^ M) in CH_3_CN–H_2_O (1 : 4) at different pH values (top), and dependence of the fluorescence intensity at 382 nm on the pH (bottom). The black dots are experimental values and the red line corresponds to a model involving a one-step protonation equilibrium.

UV-vis and fluorescence titrations in acidic media were performed to determine the p*K*_a_ values and corresponding 
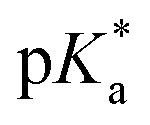
 values in the S_1_ state for 1–3 (Table 1). Fig. 3 and 4 show the spectra for 3, and those of 1 and 2 are included in the ESI (Fig. S12–S18).† Increasing the acidity of the solution resulted in a hypochromic shift of the lowest-energy absorption band for 1–3 (*i.e.*, due to protonation of the nitrogen atom) and a quenched fluorescence. The lower fluorescence quantum yields of 1–3 in acidic solutions are in line with an observed efficient photoreaction in acidic media. As shown in Table 1, compounds 1–3 display decreased basicity in the S_1_ state (lower 
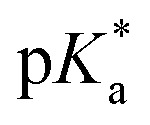
 values). These results suggest that upon photoexcitation, electron density on the nitrogen atom is delocalized into the aromatic ring, and the nitrogen atom becomes less basic (see computed NPA charges for N in Fig. 6).

**Table 1:** Measured p*K*_a_ and 
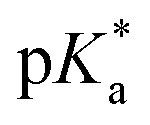
 values of 1–3 by UV-vis and fluorescence titration^a^

Compound	p*K*_a_ (UV-vis)	p*K*_a_ (fluorescence)	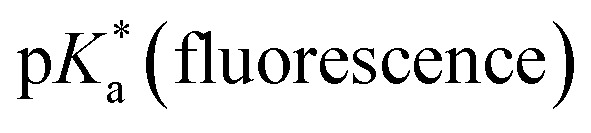
1	3.48 ± 0.02	3.27 ± 0.02	0.56 ± 0.04
2	3.94 ± 0.03	3.74 ± 0.06	0.15 ± 0.03
3	3.75 ± 0.01	3.80 ± 0.04	1.49 ± 0.02

a All titration experiments were conducted in CH_3_CN–H_2_O (1 : 4) at 25 °C.

### Irradiation experiments

Irradiating 1–3 in CH_3_CN–D_2_O (2 : 1) at pD = 2 at ≈300 nm led to regiospecific deuteration: specifically, at the C2′ position of the phenyl ring in 1 and 3, and the C4 position of the aniline ring in 2. Additionally, irradiating 2 gave a photoredox product 4 (*vide infra*). The acidity of the mixture was adjusted by addition of D_2_SO_4_ or a NaOD–D_3_PO_4_ buffer. The content of deuteration and position of the deuterium in molecules 1–3 was determined after aqueous workup from MS and ^1^H NMR, respectively. Experimental procedures and graphs showing dependence of D-exchange with the irradiation time, the D_2_O concentration, and the pH (pD) are included in the ESI (Fig. S19–S34).† Experiments for the naphthyl derivative of 1 under similar acidic conditions (pD = 2 in the presence of D_2_SO_4_) also showed efficient D-exchange.^20^

**Scheme 2:**
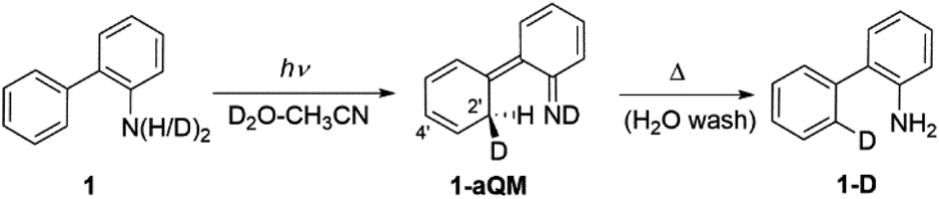
ESIPT reaction for D-exchange in 1.

Irradiating 1 in CH_3_CN–D_2_O led to regiospecific D-exchange at the C2′position of the phenyl ring and yielded 1-D (Scheme 2). Deuteration depends on irradiation time (Fig. S19–S21 in the ESI†); 3 h irradiation gave almost complete D-exchange at the C2′ position, and upon longer irradiation, both *ortho*-H-atoms of the phenyl ring were replaced by D. A control experiment for 1 in CH_3_CN–D_2_O (2 : 1) at pD = 2 without irradiation showed no sign of D-exchange, confirming that D-exchange is a photochemical process. Moreover, the deuteration takes place over wide pH (pD) range with similar efficiency (see Fig. S23 in the ESI†). Most importantly, the D-exchange at pH ≈ 7 is not in agreement with a plausible mechanism that would involve electrophilic attack of H_3_O^+^ (D_3_O^+^), *vide infra*. A proposed reaction is shown in Scheme 2.^6,20^ Upon irradiation, 1 undergoes ESIPT giving aza-quinone methide 1-aQM, and the intermediate then re-aromatizes to 1-D. The last step is expected not to be pH- (pD-) dependent. The quantum yield for D-exchange in 1 (*Φ*_R_ = 0.016 ± 0.004, Table S4†) in CH_3_CN–D_2_O (2 : 1, v/v, pD = 2) was determined by use of KI/KIO_3_ as an actinometer (*Φ*_254_ = 0.74).^45,46^ This value is lower compared to the quantum yield for D-exchange in 2-phenylphenol (*Φ*_254_ = 0.041 ± 0.004),^6^ as expected by the lower acidity of the NH_2_*versus* OH group.

We found that the efficiency of D-exchange increases upon increased D_2_O content (between 0.1% to 1% D_2_O) and levels off at concentrations higher than 1% (see Fig. S22 in the ESI†). Interestingly, a higher D_2_O concentration does not lead to regioselective D-exchange at the more distant C4′ position. These results contrast with Wan's 2002 report on 2-phenylphenol, which showed both C2′ and C4′ deuterated photoproducts.^6^ It was found that, upon irradiating 2-phenylphenol, D-exchange at the C2′ position takes place *via* an ESIPT mechanism, whereas at higher D_2_O concentration, D-exchange at the C4′ position occurs *via* a H_2_O-mediated ESPT mechanism. Thus, deuteration of position C4′ may also take place in 1, but it is significantly less efficient compared to the ESIPT to position 2′. Note that we detected some tri-deuterated compound 1 after long irradiation (Fig. S19 in the ESI†), which may be due to the less efficient deuteration of the 4′ position, not detectable by ^1^H NMR (<5%).

**Scheme 3:**
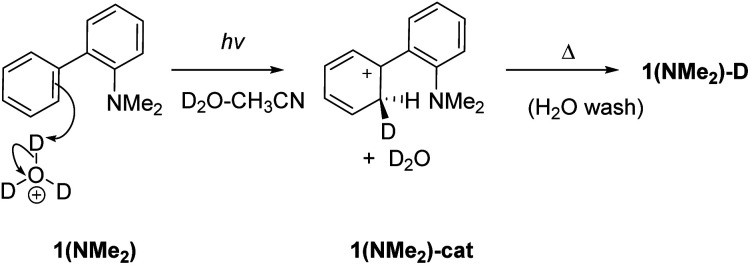
Photoinduced electrophilic attack of D_3_O^+^ at C2 for D-exchange in 1(NMe_2_). This mechanism is likely not important except for very acidic conditions.

To investigate whether D_2_O is required for D-exchange, we prepared a deuterated version of 1 (ND_2_, see the ESI† for details) and irradiated it in anhydrous CH_3_CN. No D-exchange occurred at the phenyl rings, suggesting that D_2_O is required for D-exchange to occur. To investigate whether an NH_2_ group is required for D-exchange, we prepared the corresponding dimethylated derivative 1(NMe_2_). Irradiated and non-irradiated samples of 1(NMe_2_) in CH_3_CN–D_2_O (2 : 1) both showed comparable minor D-exchange, more than ten times less efficient than for 1 (Table S7†), indicating that an NH_2_ group is necessary for D-exchange. To investigate whether photoinduced electrophilic attack of D_3_O^+^ to the carbon atom site contributes to D-exchange, we irradiated 1 in CH_3_CN–D_2_O in the presence of deuterated sodium phosphate buffer at different pD values (Table S6 and Fig S23 in the ESI†). D-exchange is low at pD < 3 and 1-D is observed at pD values 3–8, suggesting that an electrophilic D_3_O^+^ attack mechanism is unlikely (although it cannot be completely ruled out that some D-exchange may operate at very low pD values with much lower efficiency). These results obtained at pH values higher than the p*K*_a_ also indicate that ESIPT involves proton transfer from an NH_2_ group (and not NH_3_^+^). Electrophilic attack of D_3_O^+^ to the deuterated carbon atom site (Scheme 3) is only competitive in very acidic solutions (*i.e.*, when the concentration of D_3_O^+^ is high enough so that bimolecular reaction can compete with fast deactivation of 1 from S_1_: *τ* = 2.5 or 3.6 ns; Tables S2 and S3†).

Compounds 2 and 3 cannot undergo ESIPT, since the acidic NH_2_ group is too far away from the neighboring phenyl ring. But water (D_2_O)-mediated excited-state proton transfer may give rise to D-exchange at distal sites.

**Scheme 4:**
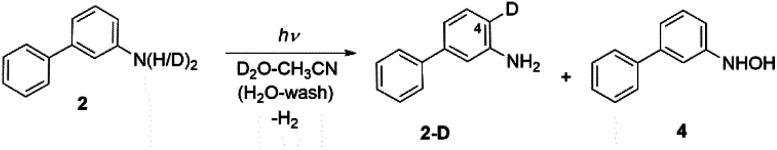
Excited-state proton transfer and photoredox reactions for 2. See Scheme 6 for the proposed photoredox mechanism for the formation of 4.

Irradiating 2 in CH_3_CN–D_2_O gave two products, a compound 2-D resulting from regioselective D-exchange at the C4 position of the aniline ring, and a photoredox product 4 (Scheme 4). The photoproduct 4 could not be separated from the starting material. Attempts of HPLC separation provided their mixture or the starting compound 2. The structure of photoproduct 4 was therefore assigned based on NMR characterization of the mixture with 2 and MS analysis (see Fig. S24–S30 in the ESI†). The ^15^N–^1^H HMBC NMR correlation of the mixture clearly points to two different N-atoms (Fig. S29†). The structure for 4 was also confirmed by chemical synthesis. We performed a reduction of 3-nitrobiphenyl with Raney nickel and obtained the same mixture as the photolysis of 2 (see Fig. S31 in the ESI†). 2-D only is observed in acidic solution and may occur by electrophilic attack of D_3_O^+^ at the C4 position *via* water-assisted D-exchange. Computed partial charges for 2 support the observed regioselectivity, showing that in the S_1_ state, C4 is the most negatively charged ring carbon atom in the aniline ring (see Fig. 6, and discussion below).

Formation of a hydroxylamine photoproduct 4 suggests that photoexcitation of 2 triggers a photoredox process. To investigate how 4 was formed, compound 2 was irradiated in CH_3_CN–H_2_O, at different pH values and in the presence of different acids (H_2_SO_4_ and HCl). Product 4 was formed regardless of the pH values and the acid used, and these results suggest that an acidic media is not required for the observed photoredox process. We surmise that a proton-coupled electron transfer (PCET) reaction occurs between 2 and H_2_O, generating an OH radical and H atom (*vide infra*), which then forms 4 and releases H_2_. Although we did not detect the formation of H_2_, it is a probable product based on the proposed photoredox mechanism.

**Scheme 5:**
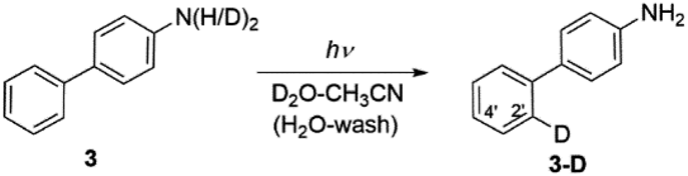
Excited-state proton transfer reaction for D-exchange in 3.

Irradiating 3 in CH_3_CN–D_2_O led to regioselective D-exchange at the C2′ position of the phenyl ring, giving 3-D (Scheme 5). D-exchange occurs only at very low pD values (see also Fig. S32–S34 in the ESI†) with a quantum yield of <10^−4^. The reaction takes place only in acidic solution and possibly proceeds through a water-assisted ESPT reaction involving D_3_O^+^. Computed partial charges for 3 support the observed regioselectivity, showing that in the S_1_ state, the C2′ and C4′ positions are most negatively charged ring carbon atoms in the phenyl ring (Fig. S52†). D-exchange at the C2′ position may be explained by a closer proximity to the NH_2_ group, making it a more accessible site *via* water-assisted ESPT. Note also that there are two C2′ positions but only one C4′ position, which may explain the observed protonation at C2′.

Do these photoreactions happen in the singlet or triplet excited state? To investigate whether the ESPT of 1–3 and the photoredox of 2 occur from the singlet or triplet excited state, the experiments were repeated in CH_3_CN–D_2_O at pD = 2 and purged by N_2_ or O_2_. O_2_ strongly quenches triplet excited-states, and thus reactions that occur in the triplet state would be significantly quenched. For 1, we observed some quenching by O_2_ (Table S9 in the ESI†), suggesting that D-exchange may involve some triplet excited-state species. However, some quenching can occur in the S_1_ state. We expect that singlet–triplet energy transfer can give rise to singlet oxygen and produce the triplet excited state of 1. Note that the energy gap between the S_1_ and T_1_ state of 1 (*E* = 27.2 kcal mol^−1^) is within a reasonable range to populate ^1^O_2_ (*E* = 22.4 kcal mol^−1^). Spin is conserved in this process. D-exchange in 2 and 3 were not significantly quenched by O_2_, suggesting that these reactions do not involve triplet excited-states. The photoredox reaction of 2 giving hydroxylamine 4 was completely quenched by O_2_, indicating that the suggested PCET reaction for 2 involves a triplet excited state.

In summary, the three aminobiphenyl isomers 1–3 are likely undergoing three different photoreactions. The *ortho*-derivative, 1, undergoes ESIPT. We expect that the *meta*-derivative, 2, undergoes water-assisted ESPT and a photoredox reaction *via* PCET. We also expect that the *para*-derivative, 3, undergoes a water-assisted ESPT reaction. All of these reactions appear to take place in the singlet excited state, except for the photoredox process of 2, which involves a triplet excited state.

### Laser flash photolysis

**Fig. 5:**
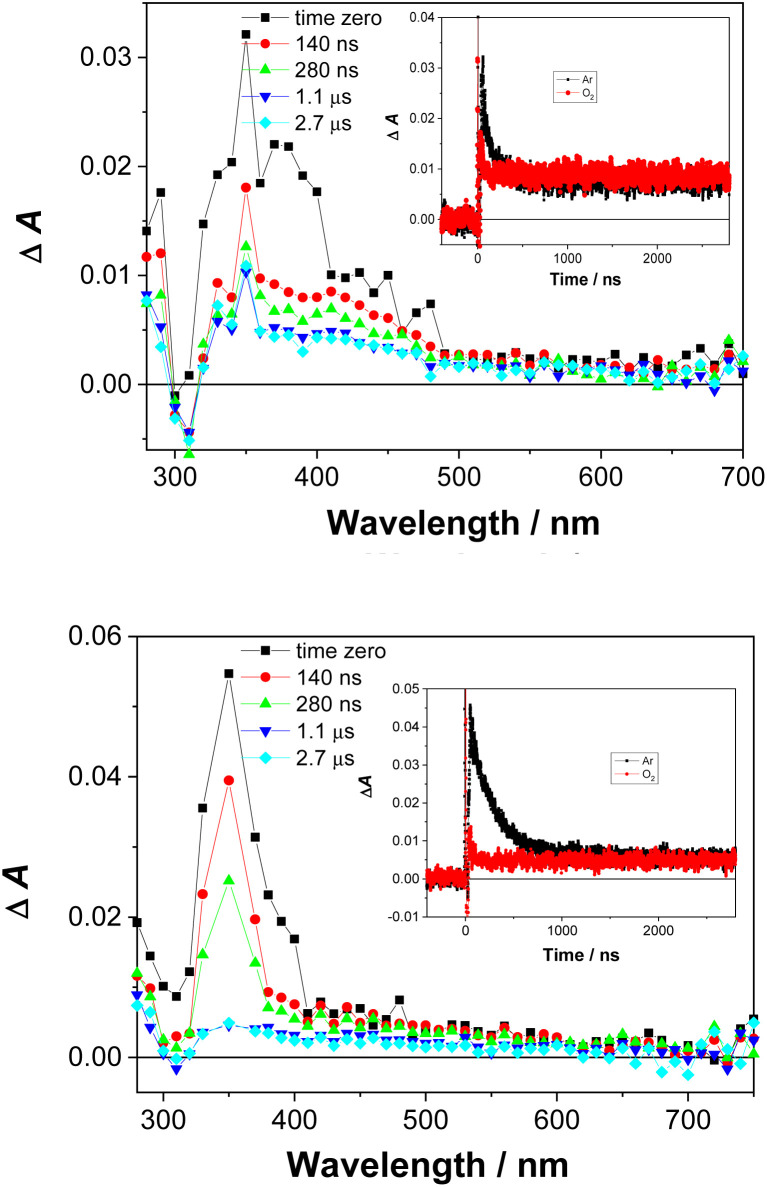
Transient absorption spectra of 1 (*c* = 1.33 × 10^−4^ M), (top) and 2 (*c* = 3.68 × 10^−5^ M), (bottom) in Ar-purged (CH_3_CN–H_2_O) (4 : 1), *A*_266_ = 0.3, laser pulse 20 mJ. Insets: the decay of transient absorption at 360 nm for the Ar- and O_2_-purged solution.

Laser flash photolysis experiments were carried out to probe for triplet excited states and reactive intermediates in the photoreactions of 1 and 2. All spectra and experimental details are included in the ESI (Fig. S35–S42†). In Ar-purged CH_3_CN and CH_3_CN–H_2_O solutions, a transient signal with a maximum of absorption at ≈350 nm was observed for 1 and 2 (Fig. 5). The transient decayed within nanoseconds (*τ* ≈ 100–300 ns) and was quenched by O_2_.

We assigned the transient signals to the triplet excited states of 1 and 2 by comparison to the transient absorption spectra of hydroxybiphenyls,^47,48^ and triplet excited states of aniline derivatives.^49,50^1-aQM was not detected, possibly due to a short lifetime and the low efficiency of its formation. It is anticipated to have a short lifetime, similar to aza-QM, detected from 2-(2-aminophenyl)naphthalene,^20^ or similar QM derivatives formed from phenols.^48^ The radical cations of 1 and 2 and solvated electron also were not detected, which preclude a photoionisation mechanism. The tentative assignments of the transients observed in the LFP of 1 and 2 is provided in the ESI.†

### Computed vertical excitations, charges, and nucleus-independent chemical shifts (NICS)

Computed vertical excitation energies for 1–3 at the ADC(2)/cc-pVDZ level are in good agreement with experimental UV-vis data (Tables S10–S12†). Plots of orbitals involved in the first three singlet excited states are included in Fig. S43–S45 of the ESI,† while the corresponding electron density difference plots are included in Fig. S46–S48.† All three compounds have ππ* character in the S_1_ state and show decreased electron density at the N atom and increased electron density in the phenyl ring in the S_1_ state. These findings are consistent with a red-shifted UV-vis spectra and quenched fluorescence at lower pH due to protonation of the N atom.

Computed Natural Population Analysis (NPA) charges at the CPCM(CH_3_CN)-PBE0-D3/def2-TZVPP level for 1–3 in the S_0_ and S_1_ states are shown in Fig. S52 of the ESI,† and these results explain the regioselectivity of D-exchange in 1–3 (partial charges computed at the MP2/cc-pVDZ and ADC(2)/cc-pVDZ levels are included to Fig. S49–S51†). For 1 and 3, all carbon atoms of the phenyl ring (besides the linker carbons) become more negatively charged in the S_1_ state; this is consistent with an observed deuteration at the phenyl ring (*i.e.*, Ring-1). In the S_1_ state of 1, C4′ of the phenyl ring bears the most negative charge, and the proximity of C2′ to the NH_2_ explains regioselective D-exchange *via* ESIPT at this position (Fig. S52†). In the S_1_ state of 3, C2′ and C4′ of the phenyl ring bear the most negative charge, yet the C2′ position is closer to the NH_2_ group and thus may be more accessible for water-assisted ESPT. For 2, all carbon atoms of the aniline ring (besides C3, which is attached to the NH_2_ group) become more negatively charged in the S_1_ state; this is consistent with an observed deuteration at the aniline ring (*i.e.*, Ring-2). In the S_1_ state of 2, C4 of the aniline ring is the most negatively charged ring carbon atom, which supports the observed regioselective D-exchange at this position.

**Fig. 6:**
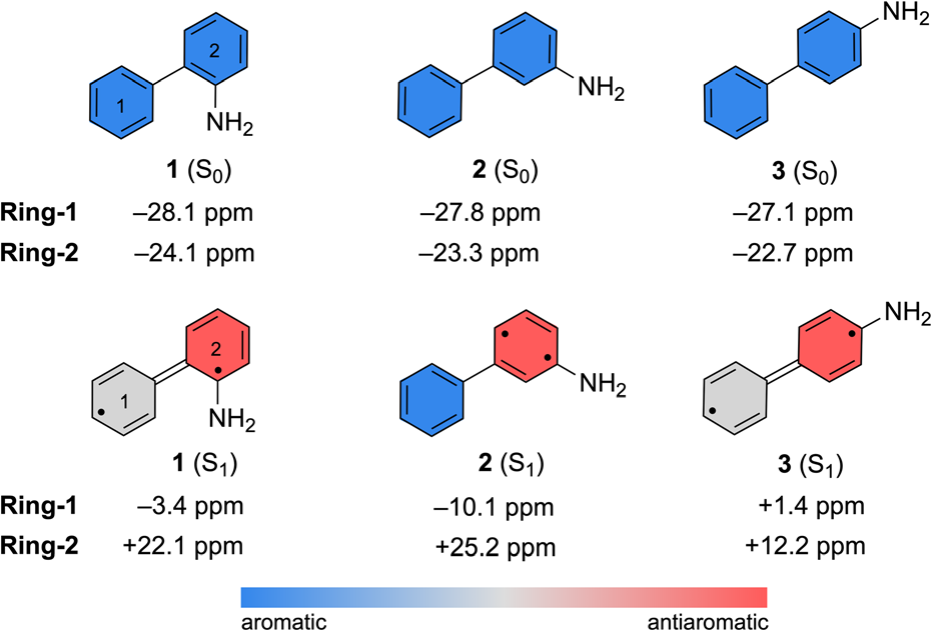
Computed NICS(1)_*zz*_ values for the S_0_ and S_1_ state minimum geometries of 1–3. NICS(1)_*zz*_ for the S_1_ state minimum geometries were computed as triplet states.

Computed nucleus-independent chemical shifts (NICS) at the CPCM(CH_3_CN)-PBE0-D3/def2-TZVPP level, based on the S_0_ and S_1_ state minimum geometries of 1–3, are shown in Fig. 6. NICS(1)_*zz*_ values were computed at the phenyl (Ring-1) and aniline (Ring-2) ring centers. NICS for the S_1_ states were computed as triplet states based on geometries optimized in the S_1_ state with TD-CPCM(CH_3_CN)-PBE0-D3/def2-TZVPP. In the ground state, all three compounds show large negative NICS(1)_*zz*_ values for Ring-1 and Ring-2, indicating strong aromatic character. In the S_1_ state, computed NICS for 1 and 3 show that Ring-1 is non-aromatic (*i.e.*, NICS(1)_*zz*_ values close to zero) and Ring-2 is antiaromatic (*i.e.*, positive NICS(1)_*zz*_ values).

In contrast, computed NICS(1)_*zz*_ for the S_1_ state of 2 reveals a localized antiaromatic character in Ring-2, while Ring-1 retains aromaticity. Differences in the (anti) aromaticity patterns of 1–3 in the S_1_ state may have mechanistic consequences. We note that in 1 and 3, excited-state antiaromatic character of the aminobiphenyl compound is delocalized between Ring-1 and Ring-2. But in 2, it is localized in Ring-2. A higher photoreactivity of the aniline fragment in 2 may explain why it undergoes a photo-induced PCET reaction and uniquely deuterates at the aniline ring. In accordance, computed spin densities for the T_1_ state of 2 shows more spin in the aniline ring, while those of 1 and 3 display delocalized spins across the two rings (Fig. S53 in the ESI†).

### Antiaromaticity relief in the ESIPT reaction of 1

**Fig. 7:**
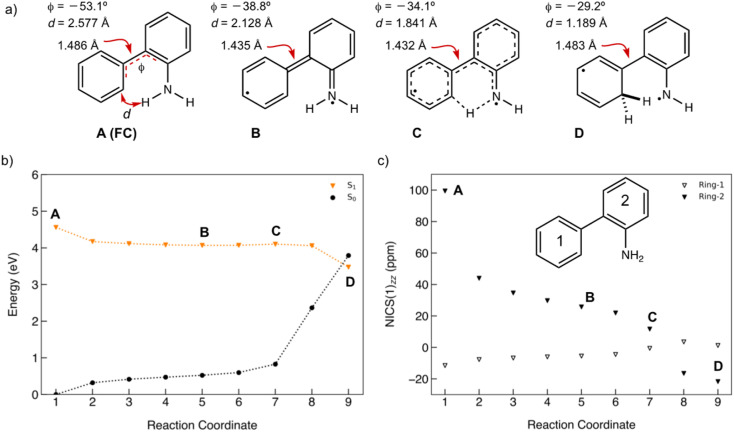
(a) Selected structures on the S_1_ state MEP for ESIPT in 1: the Franck–Condon point (A), the lowest energy point on the S_1_ state MEP (B), the highest energy point after (B) and (C), and a post-transition structure (D). *d* is the distance between the migrating H and the proton accepting carbon atom. *Φ* is the CCCC dihedral angle across the central linker CC bond. (b) A plot of the S_1_ state MEP computed at ADC(2)/cc-pVDZ for the ESIPT reaction of 1. Single-point S_0_ state energies were computed for each structure at MP2/cc-PVDZ. (c) NICS(1)_*zz*_ values computed at CPCM(CH_3_CN)-PBE0-D3/def2-TZVPP for structures along the MEP (*cf.* orange curve in Fig. 7b).

A minimum energy pathway (MEP) was computed at the ADC(2)/cc-pVDZ level to investigate the ESIPT reaction of 1. Relevant structures (A–D) along the MEP are shown in Fig. 7a. Plots of the S_1_ state MEP and S_0_ state single-point energies at MP2/cc-pVDZ are shown in Fig. 7b. Structure A is the Franck–Condon point. Structure B is the lowest energy point on the MEP. Structure C is a transition state-like structure (*i.e.*, proton is transferring—not necessarily a first-order saddle point) and the highest energy point after B. Structure D is a post-transition structure and the last point on the MEP considered. A few important observations emerge from Fig. 7: (1) the ESIPT reaction of 1 is a near-barrierless process in the S_1_ state. (2) As 1 relaxes from the Franck–Condon point, there is significant electron delocalization across the biphenyl linker (note shortened linker C–C bond length in B, as shown in Fig. 7a). (3) As D forms, the S_1_ and S_0_ state surfaces cross and a conical intersection brings the excited-state species back to the ground state. A computed MEP for 1 with H_2_O is included to Fig. S54, Tables S17 and S18 in the ESI,† showing a similar energy profile for ESIPT as that shown in Fig. 7. Cartesian coordinates of points A–D are included to Tables S13–16.†

Nucleus-independent chemical shift tensor components of the principal axis perpendicular to the ring plane (NICS(1)_*zz*_)^51–54^ were computed at 1 Å above the centroids of Ring-1 (Fig. 7c, white triangles) and Ring-2 (Fig. 7c, black triangles) for each energy point along the MEP for the ESIPT reaction of 1.^53^ At A, Ring-1 (NICS(1)_*zz*_ = −11.5 ppm) is aromatic and Ring-2 (+99.5 ppm) is strongly antiaromatic (see also computed spin density for A in Fig. S53†). From there, Ring-1 remains relatively non-aromatic throughout the ESIPT process (B to D) ranging from −5.5 ppm to +1.3 ppm. Yet, Ring-2 alleviates antiaromaticity as A (+99.5 ppm) relaxes to B (+25.8 ppm), and re-gains aromaticity as C (+11.7 ppm) evolves to D (−21.7 ppm). Changes in the geometries from A to D also are consistent with the effects of antiaromaticity relief and aromaticity gain in Ring-2 (Fig. 7a). From A to B, the linker C–C bond shortens (from 1.486 Å to 1.435 Å) and the dihedral angle of the biphenyl linker tends towards planarization (*φ* = −53.1° to *φ* = −38.8°, Fig. 7a), indicating increased quinoidal character in both rings in B. From C to D, the incipient C–H bond forms, and the linker C–C bond lengthens (from 1.432 Å to 1.483 Å) as the dihedral angle of the biphenyl linker twists even more towards planarity (from *φ* = −34.1° to *φ* = −29.2°), resulting in rearomatization of Ring-2 in D. NICS(1)_*zz*_ values for all S_1_ state structures along the MEP were computed as triplet states at the CPCM(CH3CN)-PBE0-D3/def2-TZVPP level. In contrast to previous examples of ESPT reactions explored by us and by others,^23,25,54^ ESIPT in 1 is accompanied both by antiaromaticity relief (*i.e.*, from relaxation of the Franck–Condon structure) and by aromaticity gain (*i.e.*, proton transfer from the NH_2_ group C2′ in Ring-2).

### Antiaromaticity relief in the PCET reaction of 2

**Scheme 6:**
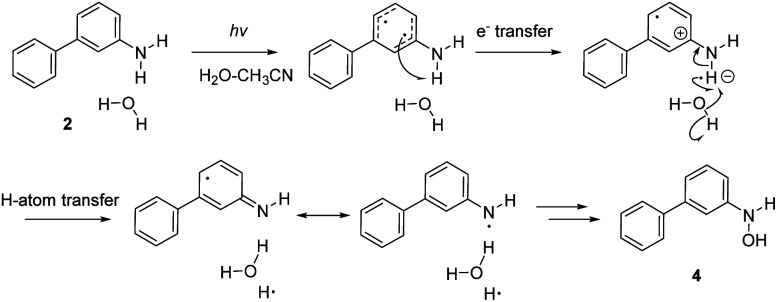
Proposed mechanism for the photoredox reaction of 2 leading to 4.

To investigate the excited-state PCET reaction of 2, we approximated the MEP with a linearly interpolated pathway in internal coordinates computed at the ADC(2)/cc-pVDZ level. We expect this to be the first step in the photoredox reaction of 2 leading to 4. Upon photoexcitation of 2, the aniline moiety undergoes a PCET reaction, and the resulting H atom splits water to produce an OH radical, which then forms a bond with the N atom, producing 4 (Scheme 6).

**Fig. 8:**
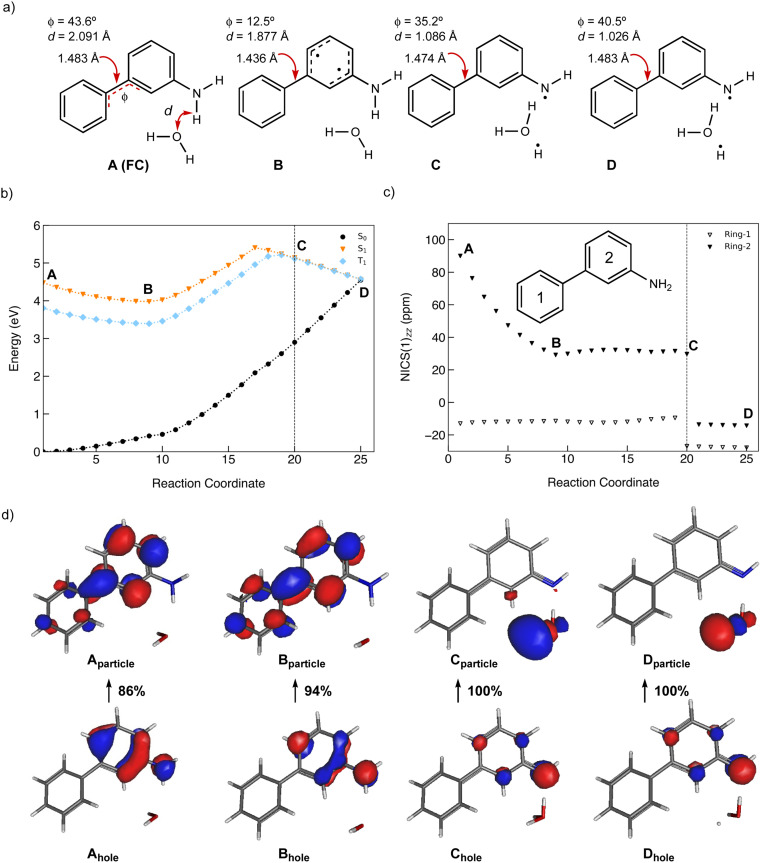
(a) Geometric features of the Franck–Condon state (A), the minimum of the S_1_ state the lowest energy point on the S_1_ state MEP (B), the highest energy point on the MEP (C), and a post-surface crossing structure (D). (b) Computed MEP plots at ADC(2)/cc-pVDZ for the PCET reaction of 2; the T_1_ and S_0_ state surfaces are computed single-point energies based on fully optimized S_1_ state geometries. *d* is the distance between the migrating H and the O atom on water. *Φ* is the CCCC dihedral angle across the central linker CC bond. (c) NICS(1)_*zz*_ values computed at CPCM(CH_3_CN)-PBE0-D3/Def2-TZVPP for structures along the MEP for the PCET reaction of 2. (d) Dominant natural transition orbital (NTO) pairs for structure A–D. % values are the contributions of an NTO pair to the excited-state wavefunction (S_1_ for A and B, T_1_ for C and D).

A scheme of the MEP for the PCET reaction of 2, including only the S_0_, S_1_, and T_1_ states, is shown in Fig. 8b. Plots of MEPs at the ADC(2) level suggest that the S_1_ is populated, and an intersystem crossing from S_1_ to T_2_ followed by internal conversion to T_1_ is involved in the PCET process (see relevant structures (A–D) along the MEP of the PCET reaction in the S_1_ and T_1_ states are shown in Fig. 8a and a plot of the MEP is shown in Fig. 8b). Single-point energies at the MP2/cc-pVDZ level were computed for the S_0_ states along the MEP. Structure A corresponds to the Franck–Condon point. Structure B is the lowest energy point on the MEP and corresponds to the minimum energy structure of the S_1_ state. Structure C is a post-crossing point where the S_1_ and T_1_ state surface cross. At structure D, the T_1_ and S_0_ surface cross, and 2 can return to the ground state by intersystem crossing. A few important observations emerge from Fig. 8: (1) the PCET reaction of 2 is a non-adiabatic process. The reaction likely begins in the S_1_ state and crosses to a triplet state (a more elaborate energy plot including computed energies in the S_2_, T_1_, and T_2_ states is included in the ESI Fig. S55†). (2) As 2 relaxes from the Franck–Condon point, there is significant electron delocalization across the biphenyl linker (note shortened linker C–C bond length in B). (3) Computed Natural Transition Orbitals (NTO's) for structures A and B show ππ* character, while structures C and D show πσ* state character, suggesting that the PCET reaction is a non-adiabatic process involving electron transfer from the excited-state π-system followed by N–H bond breaking. (4) At structure D, the T_1_ and S_0_ state surfaces cross and intersystem crossing brings the excited-state species back to the ground state.

Changes in the geometry of 2 along the MEP for an excited-state PCET reaction show the effects of antiaromaticity relief. The C–C bond linker in structure A (1.483 Å) shortens as the structure relaxes from the Franck–Condon region to B (1.436 Å), and the dihedral angle of the two phenyl rings tends towards planarization (from *φ* = 43.6° to *φ* = 12.5°). Planarization allows the triplet spin to delocalize which alleviates excited-state antiaromaticity in Ring-2 (*cf.*Fig. 8c). Already in B, the forming O⋯H bond shortens from 2.091 Å (in A) to 1.877 Å.

Upon electron transfer from the aniline ring to the amine H atom, the geometry at C suggests the O–H bond is almost fully formed (*d* = 1.121 Å) and the C–C bond linker length (1.474 Å) and dihedral angle (35.2°) is returned to a Franck–Condon-like geometry. Computed T_1_ NICS(1)_*zz*_ values for Ring-1 and Ring-2 along the MEP show that Ring-1 remains slightly aromatic during the PCET process (hovering around −12 ppm), and Ring-2 experiences a gradual decrease in antiaromaticity. At points 19 and 20, Ring-1 and Ring-2 undergo a drastic drop in NICS(1)_*zz*_ magnitude, corroborating a PCET process that restores aromaticity at these points in rings 1 and 2, respectively.

### Antiaromaticity relief in the water-assisted ESPT reaction of 3

**Fig. 9:**
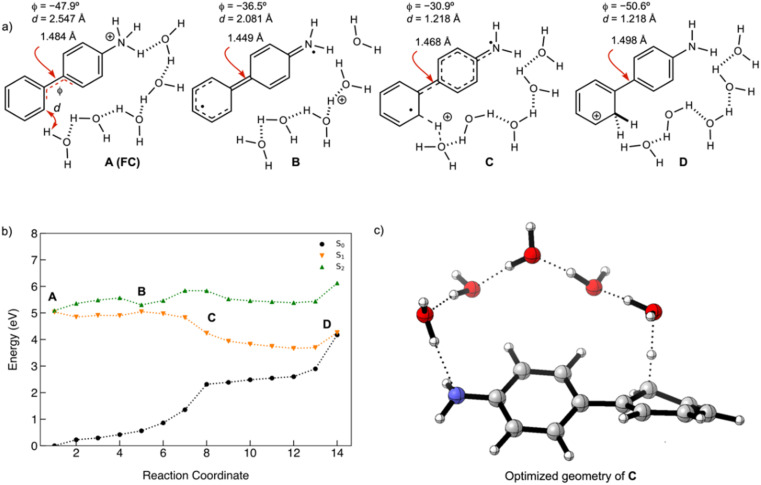
(a) Geometric features of the Franck–Condon state (A), S_2_–S_1_ near-crossing on MEP (B), ESPT to Ring-1 (C), and S_1_–S_0_ conical intersection (D). (b) Computed MEP at the ADC(2)/cc-pVDZ for the water-assisted ESPT reaction of 3 in the S_1_ state, and singlet-point energies in the S_0_ and S_2_ states. (c) Geometry for C showing proton transfer to the 2′ position in the benzene ring.

Irradiating 3 in CH_3_CN–D_2_O showed selective D-exchange at the C2′ position (Scheme 5). To investigate the origin of this D-exchange, a MEP of 3 in a micro-solvated environment of five water molecules was computed on the S_1_ with ADC(2)/cc-pVDZ (Fig. 9a and b). Note that it is only a model, and in a real system more H-bonded H_2_O molecules are involved. Furthermore, such a relay processes are entropically unfavoured, and therefore, the process takes place with a low quantum yield, as observed in the experiment. Relevant structures (A–D) for the MEP of 3 are shown in Fig. 9a. The Franck–Condon structure (A) shows the hydrogen-bonded water chain connecting the ammonium moiety to the C2′ position. Intermediate structures, B and C, show two points along the MEP where a proton is being transferred intermolecularly to another H_2_O and C2′ carbon, respectively. Structure D is the point at which the S_1_ and S_0_ surfaces cross, and where the structure is brought back to the ground state *via* a conical intersection. Geometric signatures along the MEP show notable C–C bond contracting and lengthening in the linker C–C, denoted by the single-headed red arrow, along with trends towards planarity between Ring-1 and Ring-2 (*φ* tends toward 0° from A to C). The energy landscape near the Franck–Condon (FC) region, A, and point B is complicated by near-crossing of the S_1_ and S_2_ state surfaces, and thus the NICS(1)_*zz*_ values for structures in this region cannot be properly described by the approach applied here. Optimized Cartesian coordinates for structures A–D are included to Tables S23–S26.†

## Conclusions

Excited-state antiaromaticity relief can play important roles in many light-driven proton and electron transfer reactions. Here, we investigate and contrast the photoprotonation mechanism of three aminobiphenyl isomers. In all three cases, a proton formally migrates from an NH_2_ group to an aromatic ring carbon atom. All three isomers, *ortho*-, *meta*- and *para*-, can undergo excited-state proton transfer involving water, as documented by deuterium exchange experiments. In addition, the *meta*-isomer can undergo a photoredox reaction, involving proton-coupled electron transfer and water-splitting. We note that, in the *meta*-isomer, excited-state antiaromatic character is localized in the aniline moiety while the benzene moiety remains weakly aromatic (Fig. 6), and argue that strong antiaromaticity in the aniline ring may be responsible for driving the observed photoredox reaction.

## Experimental and computational methods

### Preparative irradiation experiment in the presence of D_2_O

2-Aminobiphenyl (1) (100 mg, 0. 62 mmol) was dissolved in CH_3_CN–D_2_O (9 : 1, v/v, 100 mL) and transferred to a quartz Erlenmeyer flask with a rubber septum. The solution was purged with a stream of N_2_ for 30 min and irradiated in a Luzchem reactor equipped with 8 lamps (1 lamp ≈ 8 W) with the maximum output at 300 nm over 4 days. The flask equipped with a stirring bar was centered in the middle of the reactor, separated 15 cm from the lamps on each side. Information of the lamps irradiance can be found from the Luzchem supplier.^55^ After the irradiation, the solution was transferred to a separation funnel and water (100 mL) was added. An extraction with CH_2_Cl_2_ was carried out (3 × 75 mL). The extracts were dried over anhydrous MgSO_4_, filtered and the solvent was removed on a rotary evaporator. The residue was chromatographed on a column of silica gel by using CH_2_Cl_2_ as an eluent to remove high-weigh material formed in the photolysis. The residue (80 mg, 80%) was analysed by ^1^H NMR and MS. Since the ^1^H NMR indicated exchange of only one H by D, the sample was dissolved again in CH_3_CN–D_2_O (100 mL, 9 : 1), purged with N_2_ and irradiated in the Luzchem reactor over 4 days. After the same workup and chromatographic purification, 45 mg (45%) was obtained that was analyzed by ^1^H NMR and MS. Details of all photolyses conducted under different conditions for molecules 1–3 can be found in the ESI.†

### 2-Amino-2,2′-bisdeuteriobiphenyl (1-2D)

^1^H NMR (600 MHz, CD_3_OD): *δ*/ppm 7.43 (d, 2H, *J* = 7.8 Hz, H-3′), 7.33 (t, 1H, *J* = 7.8 Hz, H-4′), 7.10 (dt, *J* = 1.5 Hz, *J* = 7.9 Hz, H-4), 7.04 (dd, 1H, *J* = 1.5 Hz, *J* = 7.5 Hz, H-6), 6.83 (dd, 1H, *J* = 0.9 Hz, *J* = 7.9 Hz, H-3), 6.77 (dt, *J* = 1.5 Hz, *J* = 7.5 Hz, H-5). ^13^C {^1^H} NMR (150 MHz, CD_3_OD): *δ*/ppm 145.1, 141.1, 141.0, 131.2, 129.7, 129.4, 129.1 (t, *J* = 5.3 Hz), 128.0, 119.5, 117.1; MS (ESI+): *m*/*z* (%) 170 (0), 171 (23), 172 (100), 173 (13).

### Computational methods

Density functional theory (DFT) calculations were carried out in Gaussian 16 (revision C.01).^56^ Computed Natural Population Analysis (NPA) charges and dissected nucleus-independent chemical shifts, NICS(1)_*zz*_, were computed at the PBE0-D3/def2-TZVPP level of theory in a conductor-like polarizable continuum model (C-PCM).^57–61^ For each point of interest NICS(1)_*zz*_ points were manually created using the Molecule software program. Bq atoms were placed 1 Å above each ring. Time-dependent density functional theory (TD-DFT) calculations were also computed in Gaussian 16 using the Tamm–Dancoff approximation. All Cartesian coordinates of structures optimized with DFT or TD-DFT are included in the ESI, Tables S28–S36.†

Ground state geometries were optimized at MP2/aug-cc-pVDZ^62^ employing the resolution-of-identity (RI) approximation.^63^ Vertical excitation energies and oscillator strengths were computed using the algebraic diagrammatic construction to second order ADC(2) method^64–66^ as implemented in Turbomole 7.6.^67^ Calculations were performed with the cc-pVDZ and aug-cc-pVDZ basis sets both in the gas phase and in solution using the implicit solvation model COSMO^68,69^ with default parameters for acetonitrile (ACN). Excited states have been characterized in terms of natural transition orbitals^70,71^ computed by retaining only singly excited coefficients of the ADC(2) wave functions.^72^

Reaction pathways in the S_1_ state have been optimized using the double-ended reaction path optimization scheme woelfling^73^ of Turbomole. All reaction pathways start at the Franck–Condon geometry and end at the S_1_/S_0_ conical intersection (CI). CI geometries were optimized using the sequential penalty constrained optimization method of Levine *et al.*^74^ with default initial values of *α* = 0.025 Hartree and *σ* = 3.5.

## Data availability

Supporting data are available in the main text and (ESI)† document, and are readily accessible at Public Documents_HrZZ-IP-2019-04-8008 at: https://mojoblak.irb.hr/s/PXPWDXZ2QCp3daC.

## Author contributions

Conceptualization: N. D., J. W., N. B.; data curation and formal analysis: J. D., C. L.; funding acquisition: N. D., J. W., N. B.; investigation: J. D., C. L., N. D.; methodology: C. L., N. D., J. W., N. B.; project administration and resources: N. D., J. W., N. B.; software: N. D., J. W.; supervision: N. D., J. W., N. B.; visualization: J. D., C. L., N. D., J. W., N. B.; writing: N. D., J. W., N. B.; review and editing: all. All authors have given approval to the final version of the manuscript.

## Conflicts of interest

There are no conflicts to declare.

## Supplementary Material

SC-015-D4SC00642A-s001
