# Supplementary material for: Excited-state antiaromaticity relief drives facile photoprotonation of carbons in aminobiphenyls
Source: Chem Sci. 2024 Mar 4;15(14):5225–37. doi: 10.1039/d4sc00642a (PMC10988595; doi:10.1039/d4sc00642a)
Supplement: SC-015-D4SC00642A-s001 [file SC-015-D4SC00642A-s001.pdf]

Electronic Supplementary Material (ESI) for Chemical Science.
This journal is © The Royal Society of Chemistry 2024

Electronic Supplementary Information

For

Excited-State Antiaromaticity Relief Drives Facile Photoprotonation of Carbons in Aminobiphenyls

Josip Draženović,[a†] Croix J. Laconsay,[b†] Nađa Došlić,[c*] Judy I-Chia Wu,[b*] Nikola Basarić[a*]

[a] Department of Organic Chemistry and Biochemistry, Ruđer Bošković Institute, Bijenička cesta 54, 10000 Zagreb, e-mail: nbasaric@irb.hr

[b] Department of Chemistry, University of Houston, Houston, TX 77204, USA E-mail: jiwu@central.uh.edu

[c] Department of Physical Chemistry, Ruđer Bošković Institute, Bijenička cesta 54, 10000 Zagreb, Croatia e-mail: nadja.doslic@irb.hr

Content:

1. Photophysical characterization (Figures S1-S11 and Tables S1- S3) S2
2. Acid-base properties (Figures S12-S18) S9
3. Synthetic procedures S14
4. Irradiation experiments and product characterization (Figures S19-S34, Tables S4-S9) S15
5. Laser Flash Photolysis (Figures S35-S42) S32
6. Computations (Figures S43-S56 and Tables S10-S36) S36
7. NMR and MS spectra S73
8. References S90

1. Photophysical characterization

Absorption spectra were recorded on a PG T80/T80+ or a Varian Cary 100 Bio spectrophotometer at rt. Fluorescence measurements were performed on a FS5 Edinburgh Instruments spectrometer by use of slits corresponding to the bandpass of 1.00 nm for the excitation and 1.50 nm for the emission. The fluorescence spectra were corrected with respect to the fluctuations in lamp intensity and detection optics. The samples were dissolved in acetonitrile and the concentrations were adjusted to absorbances of less than 0.1 at the excitation wavelengths 280, 290, and 300 nm. The solutions were purged by N_2 for 20 min prior to the measurements. The measurements were conducted at 25 °C.

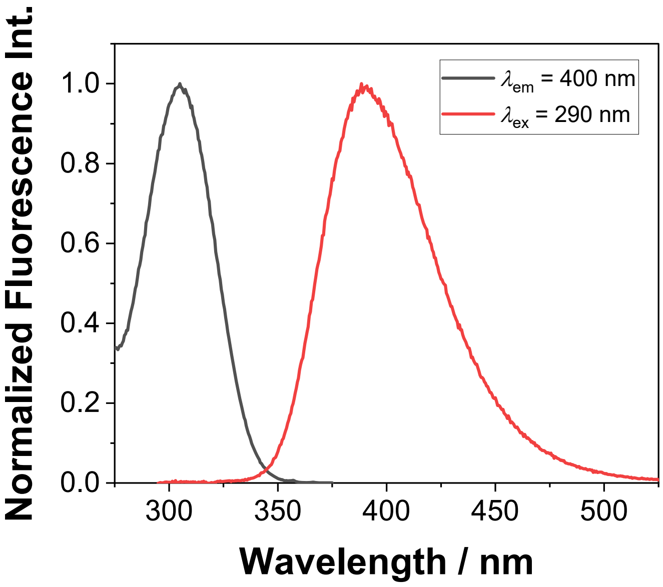

Figure S1. Normalized excitation (λ_{em} = 400 nm) and emission (λ_{ex} = 290 nm) spectra of 2-aminobiphenyl (**1**) in CH_3CN.

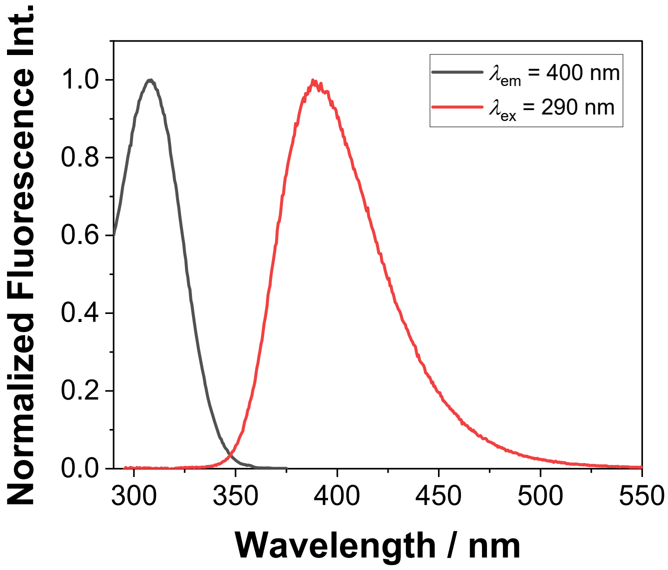

Figure S2. Normalized excitation (λ_{em} = 400 nm) and emission (λ_{ex} = 290 nm) spectra of 3-aminobiphenyl (**2**) in CH_3CN.

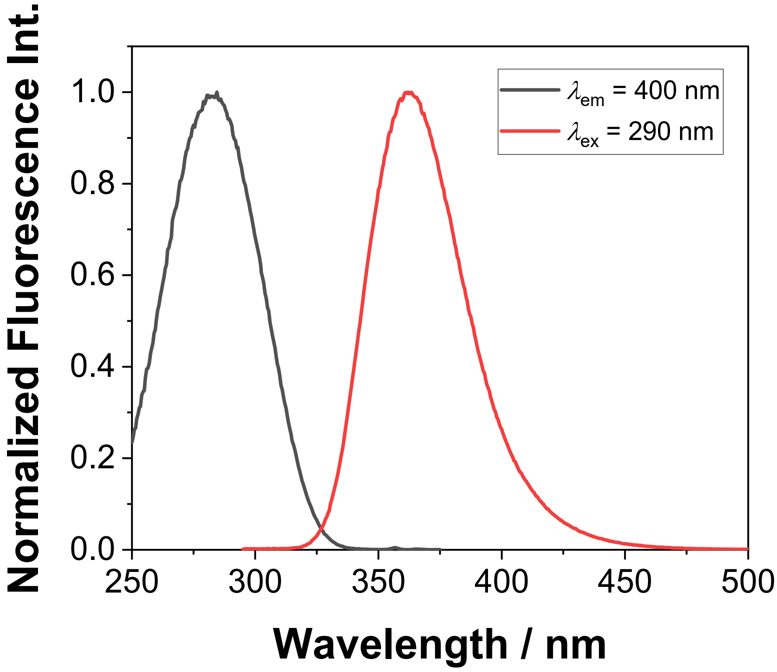

Figure S3. Normalized excitation (λ_{em} = 400 nm) and emission (λ_{ex} = 290 nm) spectra of 4-aminobiphenyl (**3**) in CH_3CN.

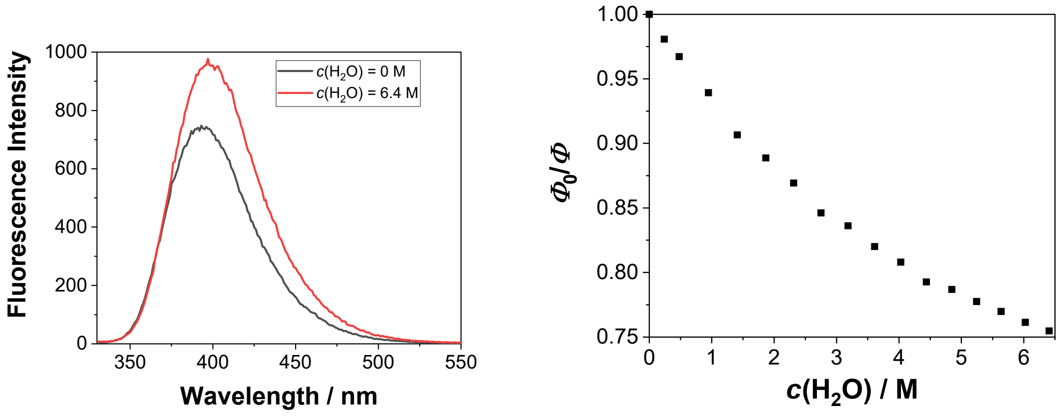

Figure S4. Fluorescence spectra of 2-aminobiphenyl (**1**) in CH_3CN at different water concentration (left) and the corresponding Stern-Volmer plot (right).

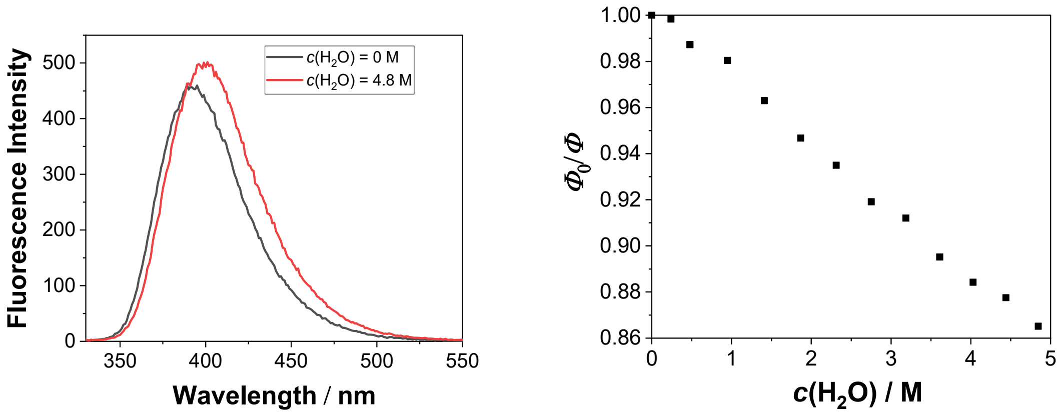

Figure S5. Fluorescence spectra of 3-aminobiphenyl (**2**) in CH_3CN at different water concentration (left) and the corresponding Stern-Volmer plot (right).

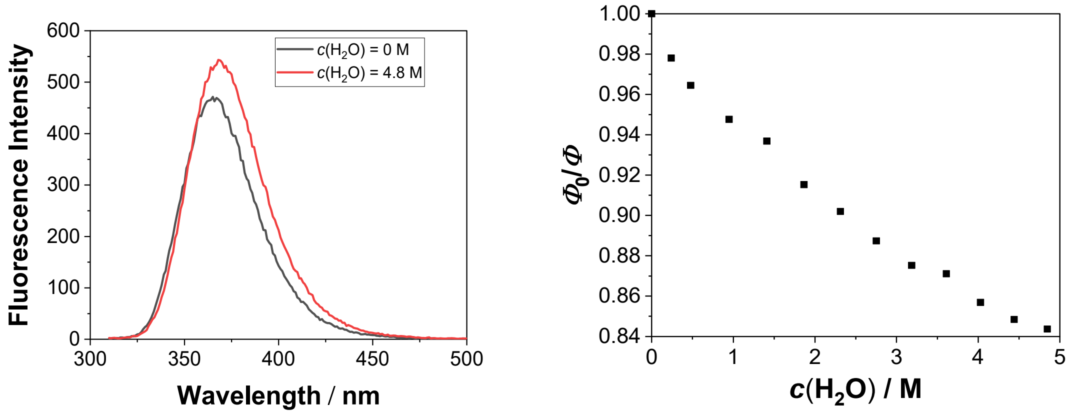

Figure S6. Fluorescence spectra of 4-aminobiphenyl (**3**) in CH_3CN at different water concentration (left) and the corresponding Stern-Volmer plot (right).

The following equation was used for the determination of fluorescence quantum yields:

$$\Phi = \Phi_R \frac{I}{I_R} \frac{A_R}{A} \left(\frac{n_D}{n_D^R}\right)^2 \qquad \text{(Eq. S1)}$$

wherein

Φ_f - quantum yield of fluorescence

Φ_R - quantum yield of fluorescence of reference compound, *N*-acetyltryptophanamide in water (0.12)

I - intensity of fluorescence (integral of the corrected emission spectrum)

I_R - intensity of fluorescence (integral of the corrected emission spectrum) for the reference compound

A - absorbance of the solution at the excitation wavelength

A_R - absorbance of the solution of the reference compound at the excitation wavelength

n_D - refractive index of the solvent (acetonitrile)

n_D^R - refractive index of the solvent use to dissolve the reference compound (water)

Table S1. Stokes shifts and quantum yields of fluorescence for CH_3CN solutions of **1**-**3**.

**Compound**	**1**	**2**	**3**
**Stokes shift / cm^{-1}**	7047	6642	7525
$\boldsymbol{\Phi_f}$ [a]	0.21±0.04	0.26±0.03	0.55±0.05

[a] Measured by use of *N*-acetyltryptophanamide in water as a reference (Φ_f = 0.12).

Time-correlated single photon counting (TC-SPC) measurements were performed on an Edinburgh Instruments FS5 spectrometer equipped with a pulsed laser at 280 nm. The duration of the pulse was ≈ 800 ps. The fluorescence signals at 370 or 390 nm were monitored over 1023 channels with the time increment of ≈ 20 ps/channel. The decays were collected until they reached 3000 counts in the peak channel. A suspension of silica gel in H_2O was used as a scattering solution to obtain instrument response function (IRF). Absorbances at the excitation wavelength were <0.1. Prior to the measurements the solutions were purged with a stream of nitrogen for 20 min. The measurement was performed at rt (25 °C). Decays of fluorescence were fit to a sum of exponentials according to the following expression:

$$F(t) = A + \alpha_1 \exp\left(-\frac{t}{\tau_1}\right) + \alpha_2 \exp\left(-\frac{t}{\tau_2}\right) + \alpha_3 \exp\left(-\frac{t}{\tau_3}\right) + \ldots \qquad \text{(Eq. S2)}$$

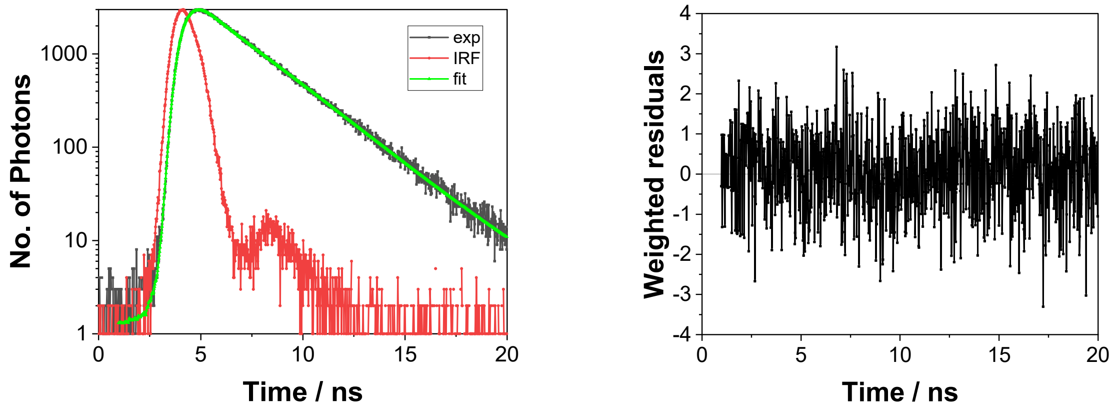

Figure S7. Decay of fluorescence of 2-aminobiphenyl (**1**) in CH_3CN (left) at 390 nm (λ_{ex} = 282 nm) and weighted residuals between the fitted and experimental values (right).

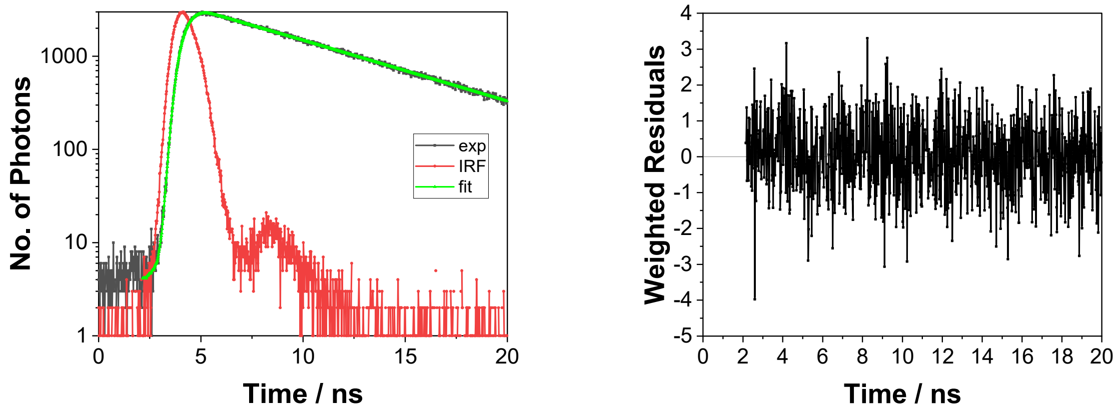

Figure S8. Decay of fluorescence of 3-aminobiphenyl (**2**) in CH_3CN (left) at 390 nm (λ_{ex} = 282 nm) and weighted residuals between the fitted and experimental values (right).

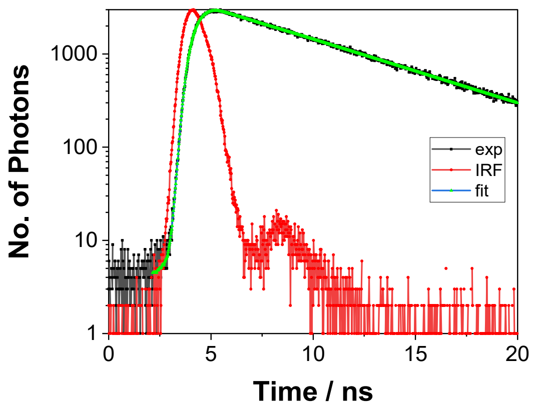

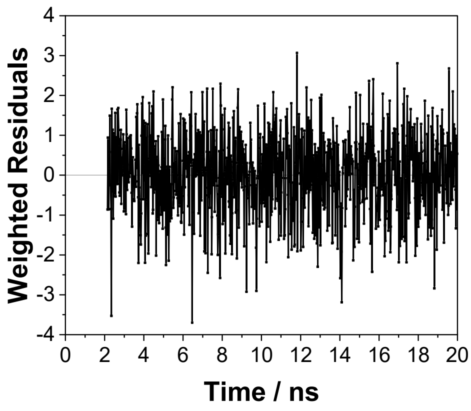

Figure S9. Decay of fluorescence of 4-aminobiphenyl (**3**) in CH_3CN (left) at 370 nm (λ_{ex} = 282 nm) and weighted residuals between the fitted and experimental values (right).

Table S2. Decay times from singlet excited state for CH_3CN solutions of **1-3** measured by TC-SPC.

	**1**	**2**	**3**
τ_1 / ps	70±20 (0.07)[a]	70±60 (0.02)	40±30 (0.03)
τ_2 / ns	2.54±0.01 (0.93)	6.59±0.01 (0.98)	6.28±0.01 (0.97)

[a] The values in the parenthesis correspond to contribution of decay component obtained from the pre-exponential factors.

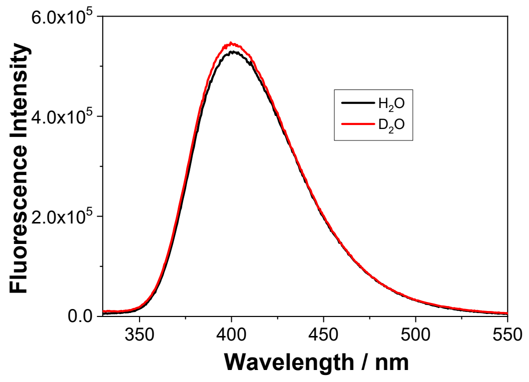

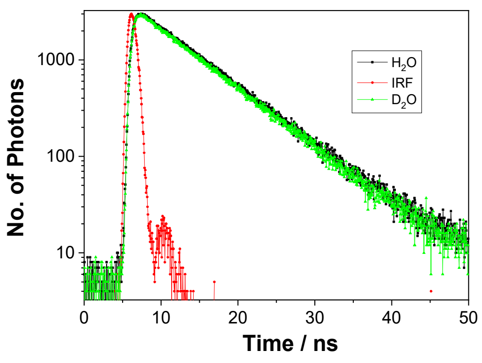

Figure S10. Fluorescence spectra of **2** (λ_{ex}= 280 nm) in CH_3CN-H_2O (1:1) or CH_3CN-D_2O (1:1), left: and the corresponding decays of fluorescence at 400 nm (right). The fitting data can be found in Table S3.

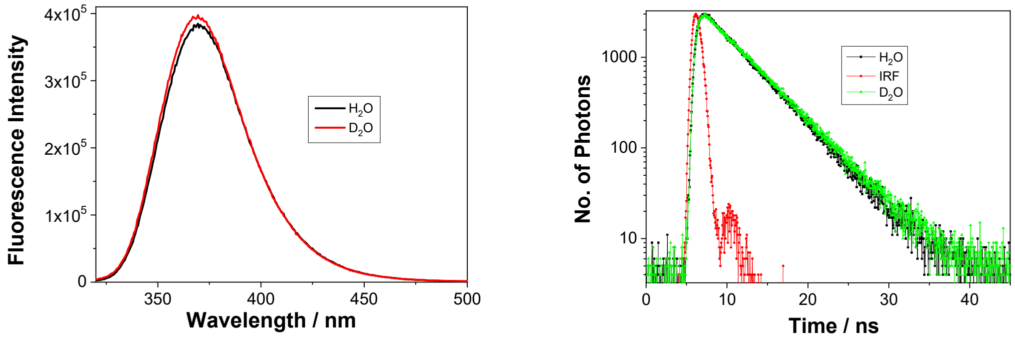

Figure S11. Fluorescence spectra of **3** (λ_{ex}= 280 nm) in CH_3CN-H_2O (1:1) or CH_3CN-D_2O (1:1), left: and the corresponding decays of fluorescence at 380 nm (right). The fitting data can be found in Table S3.

Table S3. Decay times (τ_1 / ps, τ_2 / ns) from singlet excited state for CH_3CN-H_2O (1:1) or CH_3CN- (1:1) solutions of **1-3** measured by TC-SPC.[a]

	**1**	**2**	**3**
H_2O	30±30 (0.05)[b]<br>3.58±0.01 (0.95)	≈50 (0.02)<br>7.12±0.01 (0.98)	80±30 (0.02)<br>4.42±0.01 (0.93)
D_2O	170±50 (0.03)<br>4.70±0.01 (0.97)	80±50 (0.02)[2]<br>6.98±0.01 (0.98)	20±20 (0.02)<br>4.63±0.01 (0.93)

[a] The solutions were not purged by N_2 prior to the measurement. [b] The values in parenthesis correspond to the decay contributions obtained from the pre-exponential factors.

2. Acid-base properties

Determination of pK_a, UV-vis titration

A stock solution of 4-aminobiphenyl (**3**, 3.88 mg) was prepared in CH_3CN (25 mL). The prepared stock solution (5 mL) was diluted to 25 mL with CH_3CN and 5 mL of this solution was diluted to 25 mL with water. The diluted solution (c = 3.67 × 10^{-5} M) was titrated with a solution of sulfuric acid (three different solution of sulfuric acid were used: c_1 = 1 × 10^{-3} M, c_2 = 1 × 10^{-2} M, c_3 = 1 × 10^{-1} M). After each addition of the acid, the pH-values were measured by a Mettler Toledo SevenMulti pH-meter and UV-vis spectra were recorded. The measurements were performed at 25 °C and the resulting spectra were processed by multivariate nonlinear regression analysis using the SPECFIT program.[1] In the analysis surface was defined by all UV-vis spectra from 211 nm to 320 nm at different pH values.

A stock solution of 3-aminobiphenyl (**2**, 8.85 mg) was prepared in CH_3CN (25 mL). The prepared stock solution (5 mL) was diluted to 10 mL with water and 5 mL of this solution was diluted to 25 mL with water. The diluted solution (c = 2.09 × 10^{-4} M) was titrated with a solution of sulfuric acid. In the analysis surface was defined by all UV-vis spectra from 281 nm to 330 nm at different pH values.

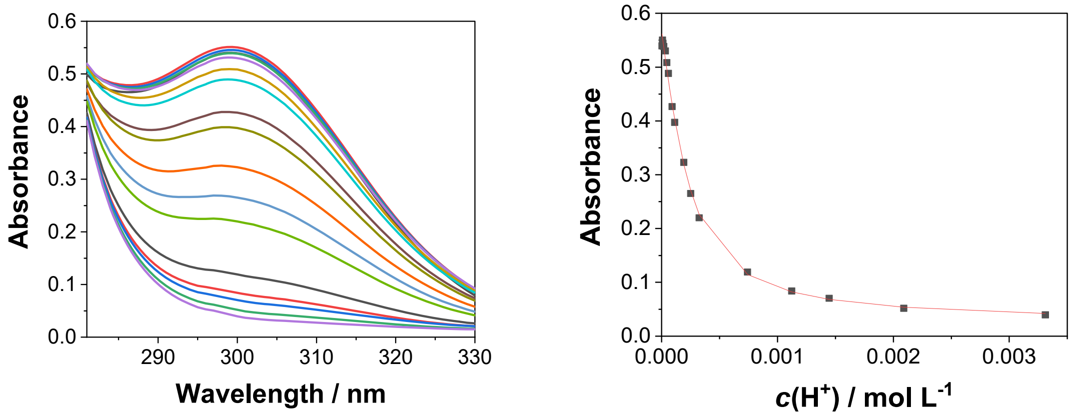

Figure S12. Absorption spectra of **2** (c_0 = 2.09 × 10^{-4} M) in CH_3CN-H_2O (1:4) at different pH values (left), and dependence of the absorbance at 300 nm on pH (right). The black dots are the experimental values and the red line correspond to the model involving one step protonation equilibrium, the pK_a value of the ground state determined by multivariate nonlinear regression analysis is 3.94 ± 0.03.

A stock solution of 2-aminobiphenyl (**1**, 4.23 mg) was prepared in CH_3CN (25 mL). The prepared stock solution (5 mL) was diluted to 25 mL with water. The diluted solution ($c = 2.00 \times 10^{-4}$ M) was titrated with a solution of sulfuric acid. In the analysis surface was defined by all UV-vis spectra from 251 nm to 325 nm at different pH values.

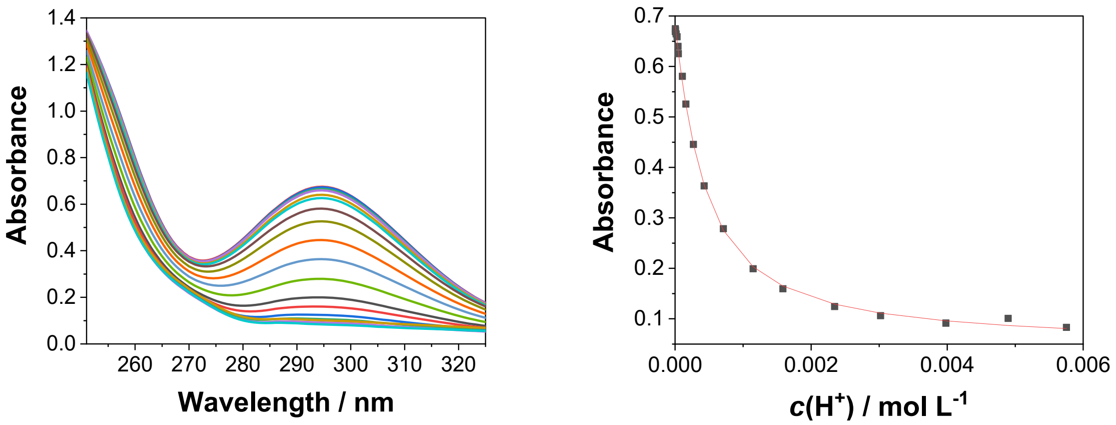

Figure S13. Absorption spectra of **1** ($c_0 = 2.00 \times 10^{-4}$ M) in CH_3CN-H_2O (1:4) at different pH values (left), and dependence of the absorbance at 295 nm on pH (right). The black dots are the experimental values and the red line correspond to the model involving one step protonation equilibrium, the pK_a value of the ground state determined by multivariate nonlinear regression analysis is 3.48 ± 0.02.

Determination of pK_a and pK_a*, fluorescence titration

A stock solution of 4-aminobiphenyl (**3**, 3.88 mg) was prepared in CH_3CN (25 mL). The prepared stock solution (0.246 mL) was diluted to 10 mL with CH_3CN and 5 mL of this solution was diluted to 25 mL with water. The diluted solution ($c = 4.51 \times 10^{-6}$ M) was titrated with a solution of sulfuric acid (four different solution of sulfuric acid were used: $c_1 = 1 \times 10^{-3}$ M, $c_2 = 1 \times 10^{-2}$ M, $c_3 = 1 \times 10^{-1}$ M, and the fourth solution was concentrated sulfuric acid). After each addition of the acid, the pH-values were measured by a Mettler Toledo SevenMulti pH-meter and fluorescence spectra were recorded by exciting sample at 260 nm and at 280 nm, by use of slits corresponding to the bandpass of 2.5 nm for the excitation and 5.0 nm for the emission. For the measurement of pK_a, the pH values were varied in the range 5.38-2.78, and for the pK_a* in the range 2.64-0.59. The measurements were performed at 25 °C and the resulting spectra were processed by multivariate nonlinear regression analysis using the

SPECFIT program. In the analysis surface was defined by all fluorescence spectra from 340 nm to 450 nm at different pH values.

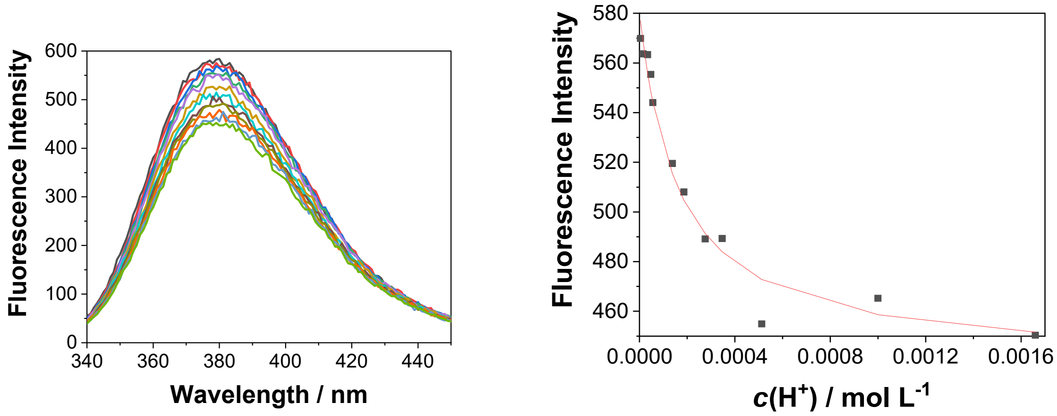

Figure S14. Fluorescence spectra (λ_{ex} = 260 nm) of **3** (c_0 = 4.51×10^{-6} M) in CH_3CN-H_2O (1:4) at different pH values (left), and dependence of the fluorescence intensity at 382 nm on pH (right). The black dots are the experimental values and the red line correspond to the model involving one step protonation equilibrium, the pK_a value of the ground state determined by multivariate nonlinear regression analysis is is 3.80 ± 0.04.

A stock solution of 3-aminobiphenyl (**2**, 8.85 mg) was prepared in CH_3CN (25 mL). The prepared stock solution (0.85 mL) was diluted to 25 mL with CH_3CN and 5 mL of this solution was diluted to 25 mL with water. The diluted solution (c = 1.42×10^{-5} M) was titrated with a solution of sulfuric acid (four different solution of sulfuric acid were used: c_1 = 1 × 10^{-3} M, c_2 = 1 × 10^{-2} M, c_3 = 1 × 10^{-1} M, and the fourth solution was concentrated sulfuric acid). After each addition of the acid, the pH-values were measured by a Mettler Toledo SevenMulti pH-meter and fluorescence spectra were recorded by exciting sample at 280 nm and at 300 nm, by use of slits corresponding to the bandpass of 10 nm for the excitation and 2.5 nm for the emission. For the measurement of pK_a, the pH values were varied in the range 4.70-2.16, and for the pK_a* in the range 1.42-0.08. The measurements were performed at 25 °C and the resulting spectra were processed by multivariate nonlinear regression analysis using the SPECFIT program. In the analysis surface was defined by all fluorescence spectra from 375 nm to 475 nm at different pH values.

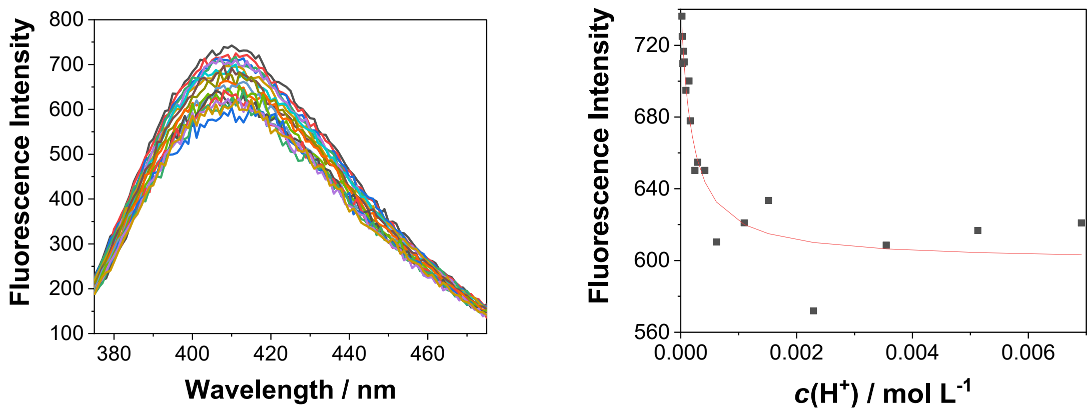

Figure S15. Fluorescence spectra (λ_{ex} = 280 nm) of **2** (c_0 = 1.42×10^{-5} M) in CH_3CN-H_2O (1:4) at different pH values (left), and dependence of the fluorescence intensity at 411 nm on pH (right). The black dots are the experimental values and the red line correspond to the model involving one step protonation equilibrium, the pK_a value of the ground state determined by multivariate nonlinear regression analysis is 3.74 ± 0.06.

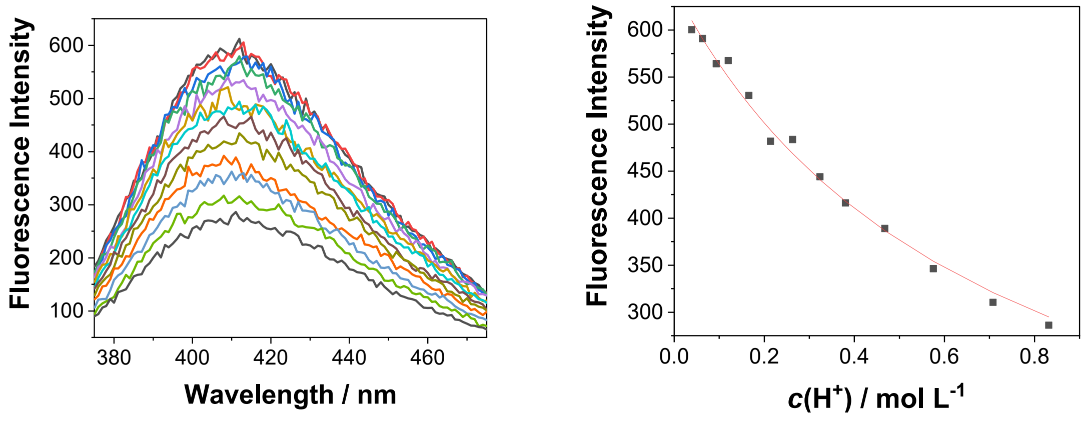

Figure S16. Fluorescence spectra (λ_{ex} = 280 nm) of **2** (c_0 = 1.42×10^{-5} M) at different pH values (left), and dependence of the fluorescence intensity at 411 nm on pH (right). The black dots are the experimental values and the red line correspond to the model involving one step protonation equilibrium, the pK_a* value of the excited state determined by multivariate nonlinear regression analysis is 0.15 ± 0.03.

A stock solution of 2-aminobiphenyl (**1**, 4.08 mg) was prepared in CH_3CN (25 mL). The prepared stock solution (0.87 mL) was diluted to 10 mL with CH_3CN and 5 mL of this solution was diluted to 25 mL with water. The diluted solution (c = 1.67×10^{-5} M) was titrated with a solution of sulfuric acid (four different solution of sulfuric acid were used: c_1 = 1 × 10^{-3} M, c_2 = 1 × 10^{-2} M, c_3 = 1 × 10^{-1} M, and the fourth solution was concentrated sulfuric acid). After

each addition of the acid, the pH-values were measured by a Mettler Toledo SevenMulti pH-meter and fluorescence spectra were recorded by exciting sample at 260 nm and at 280 nm, by use of slits corresponding to the bandpass of 10 nm for the excitation and 2.5 nm for the emission. For the measurement of pK_a, the pH values were varied in the range 5.38-2.34, and for the pK_a* in the range 1.56-0.09. The measurements were performed at 25 °C and the resulting spectra were processed by multivariate nonlinear regression analysis using the SPECFIT program. In the analysis surface was defined by all fluorescence spectra from 370 nm to 470 nm at different pH values.

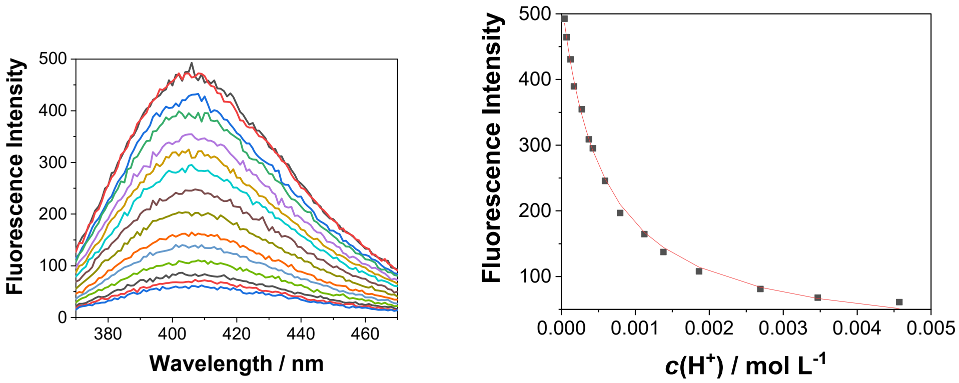

Figure S17. Fluorescence spectra (λ_{ex} = 280 nm) of **1** (c_0 = 1.67×10^{-5} M) in CH_3CN-H_2O (1:4) at different pH values (left), and dependence of the fluorescence intensity at 406 nm on pH (right). The black dots are the experimental values and the red line correspond to the model involving one step protonation equilibrium, the pK_a value of the ground state determined by multivariate nonlinear regression analysis is 3.27 ± 0.02.

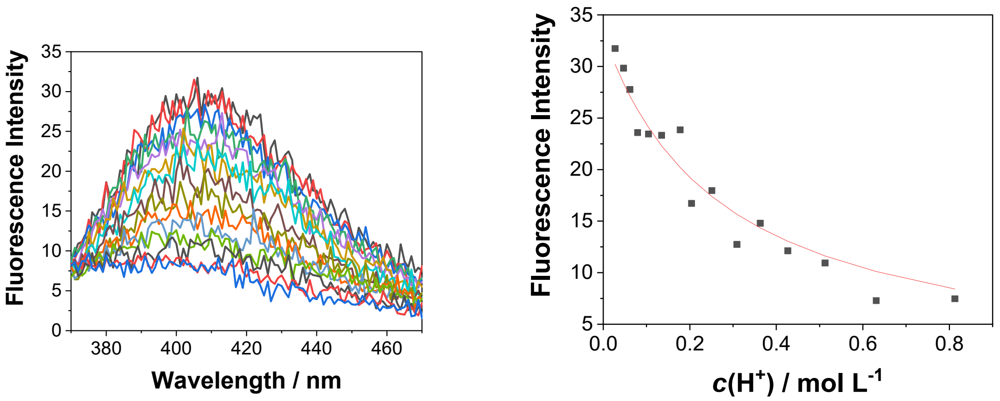

Figure S18. Fluorescence spectra (λ_{ex} = 280 nm) of **1** (c_0 = 1.67×10^{-5} M) at different pH values (left), and dependence of the fluorescence intensity at 406 nm on pH (right). The black dots are the experimental values and the red line correspond to the model involving one step protonation equilibrium, the pK_a* value of the excited state determined by multivariate nonlinear regression analysis is 0.56 ± 0.04.

3. Synthetic procedures

***N,N*-dimethyl-2-aminobiphenyl**

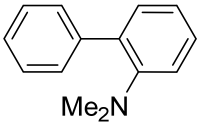

The compound was prepared according to the modification of the published procedure for the methylation of arylamines.[2] In a flask (100 mL), 2-aminobiphenyl (2.0 g, 12 mmol) and sodium hydrogen phosphate (4.3 g, 30 mmol) were suspended in dry DMF (25 mL) and methyl iodide (4.5 mL, 72 mmol) was added. The reaction mixture was stirred at rt for 24 h. The reaction mixture was diluted with water (50 mL) and extracted with ethyl acetate (3×25 mL). The organic layers were combined, washed with brine (2×25 mL) and distilled water (3×25 mL). The organic layer was dried over magnesium sulfate and concentrated in vacuum to afford 1.3 g (55 %) of the product in the form of a yellowish oil.

^{1}H NMR ($CDCl_3$, 300 MHz) δ/ppm: 7.53-7.51 (m, 2H) 7.36 (tt, 2H, J = 7.7 Hz, J = 1.6 Hz) 7.26 (tt, 1H, J = 7.3 Hz, J = 1.4 Hz), 7.23 (ddd, 1H, J = 8.1 Hz, J = 7.4 Hz, J = 1.8 Hz), 7.15 (dd, 1H, J = 7.5 Hz, J = 1.7 Hz), 7.05 (dd, 1H, J = 8.1 Hz, J = 1.1 Hz), 6.98 (dt, 1H, J = 7.4 Hz, J = 1.2 Hz), 2.49 (s, 6H); ^{13}C {^{1}H} NMR ($CDCl_3$, 75 MHz) δ/ppm: 151.1, 142.0, 134.4, 131.1, 128.3, 127.9, 127.8, 126.1, 121.4, 117.4, 42.4.

4. Irradiation experiments

Time dependence

2-aminobiphenyl (**1**, 35 mg) was dissolved in CH_3CN (70 mL). An aliquot (10 mL) of the prepared solution was added to a quartz test tube and the prepared sample was diluted by solution of D_2SO_4 in D_2O (5 mL, pD = 1.94). In this way, seven samples were prepared. Each sample was purged with nitrogen for 15 min and irradiated in a Luzchem LZC-4X photochemical reactor (8 lamps, 300 nm) over different time. After the irradiations, the samples were diluted with a saturated aqueous solution of $NaHCO_3$ (50 mL) and extracted with dichloromethane (3×25 mL). The combined organic layers were dried over sodium sulfate and concentrated in vacuum. Deuterium incorporation was determined by ^{1}H NMR and MS.

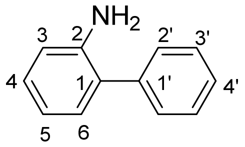

2-aminobiphenyl (**1**) ^{1}H NMR (600 MHz, CD_3OD): δ/ppm 7.43 (dd, 2H, J = 6.9 Hz, J = 8.2 Hz, H-3'), 7.40 (d, 2H, J = 8.2 Hz, H-2'), 7.33 (t, 1H, J = 6.9 Hz, H-4'), 7.10 (dt, J = 1.5 Hz, J = 7.9 Hz, H-4), 7.04 (dd, 1H, J = 1.5 Hz, J = 7.5 Hz, H-6), 6.83 (dd, 1H, J = 0.9 Hz, J = 7.9 Hz, H-3), 6.77 (dt, J = 1.5 Hz, J = 7.5 Hz, H-5).

Upon irradiation, the intensity of the signal at δ 7.40 ppm corresponding to H-2' is reduced (see Fig S20). Upon longer irradiation time, higher extend of D-exchange can be achieved, see NMR spectra at the end of this SI.

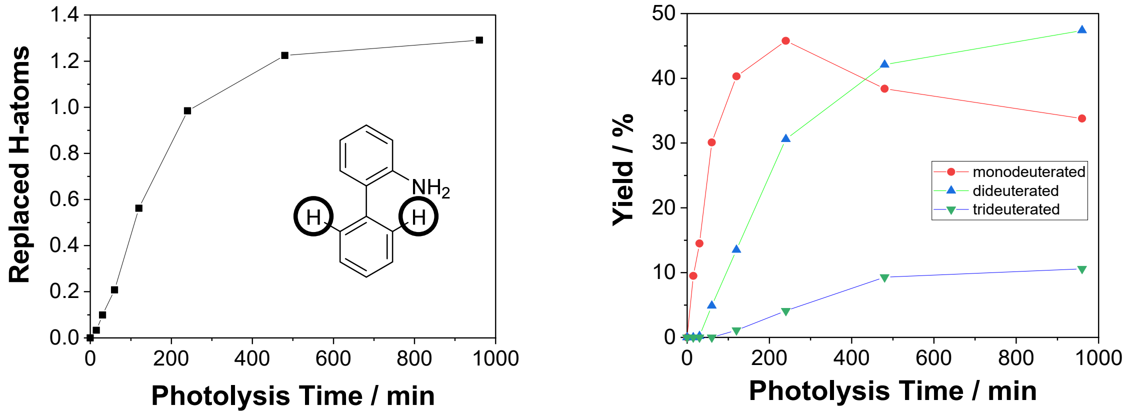

Figure S19. The dependence of the deuterium exchange at the positions 2 and 2' (marked positions) of the phenyl group in 2-aminobiphenyl (**1**) on photolysis time, determined by ^{1}H NMR (left) and the yield of mono- and dideuterated compound determined by MS (right, for MS data see Figure S21). The irradiation was performed in D_2O-CH_3CN (1:2) at pD≈2.

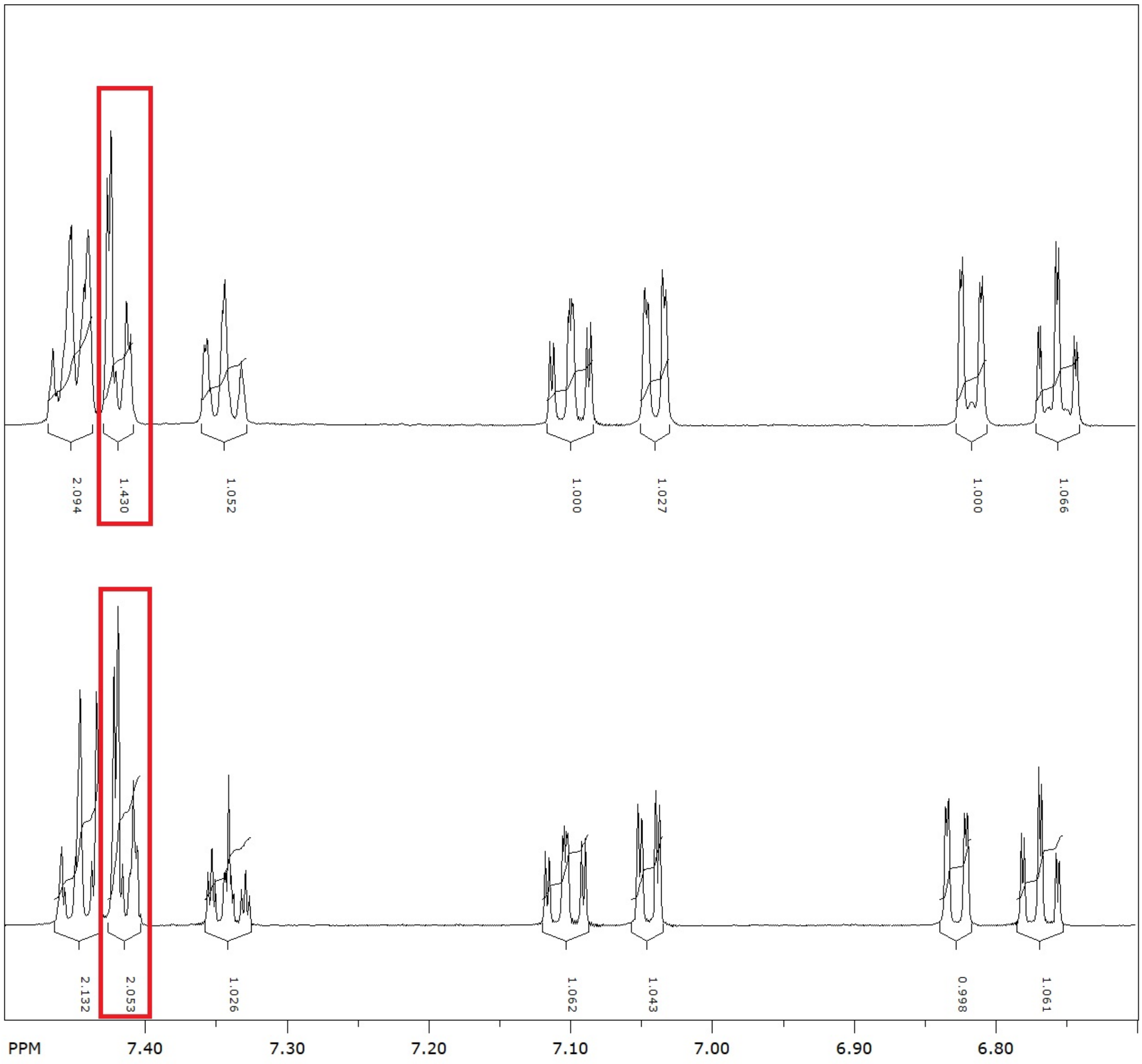

Figure S20. Expanded aromatic region of the ^{1}H NMR (CD_3OD, 600 MHz) spectra of **1** before (bottom) and after (top) the photolysis (2 h, 300 nm) in D_2O-CH_3CN (1:2).

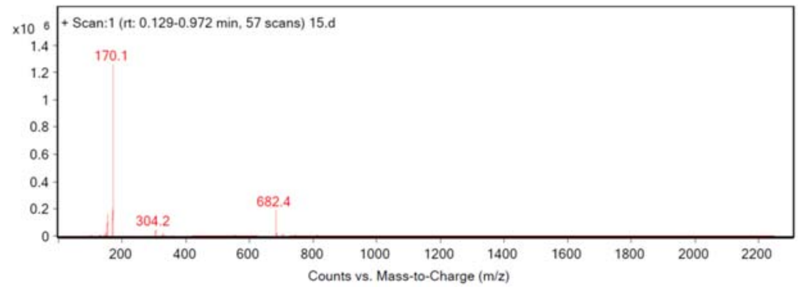

170.1		1257549
171.1	1	294099.34
172.1	1	32035.7

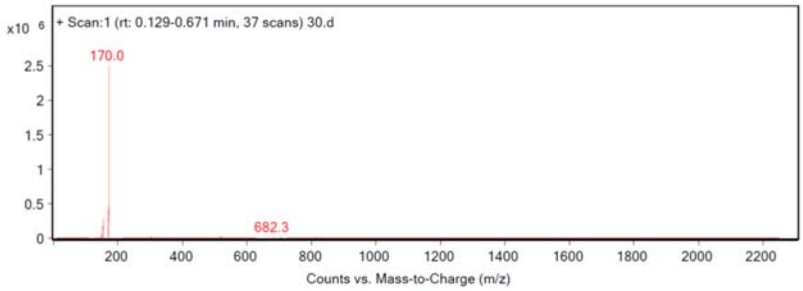

169		40914.48
170		2515595.75
171	1	752816.56
172.1	1	104845.41

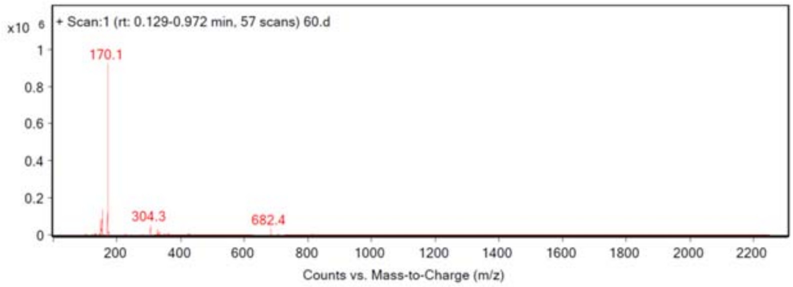

170.1		925038.25
171.1		549233.06
172.1	1	140486.77

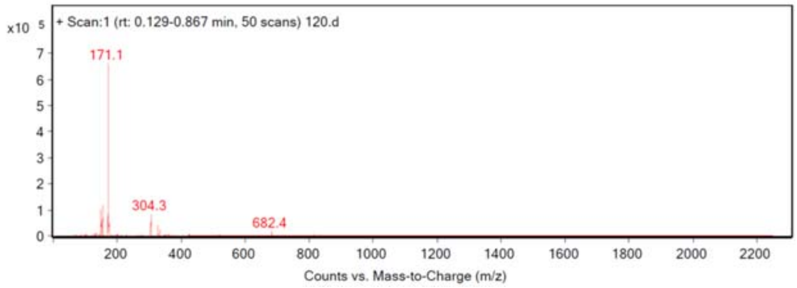

170.1		644811.88
171.1		660241.44
172.1	1	278381.06
173.1	1	51888.98
[illegible]	[illegible]	[illegible]

Figure S21. MS (ESI+) data after irradiation (300 nm) of **1** in D_2O-CH_3CN (1:2) at pD≈2, after 15 min, 30 min, 60 min and 120 min (from top to bottom); the right panels show ion abundances. The D-exchange content was plotted vs time and shown in Figure S19.

Quantum yield of deuterium exchange in 2-aminobiphenyl

2-Aminobiphenyl (**1**) (3 mg) was dissolved in CH_3CN (2 mL) and a solution of D_2SO_4 in D_2O (pD = 2.05, 1 mL) was added. The solution was purged with nitrogen for 15 min and irradiated 12 h at 254 nm (1 lamp) in a quartz cell for fluorescence measurements. After the irradiation, the samples were diluted with a saturated solution of $NaHCO_3$ (20 mL) and extracted with dichloromethane (3×20 mL). The combined organic layers were dried over sodium sulfate and concetrated in vacuum. The deuterium incorporation was determined by ^{1}H NMR and MS. As

a primary actinometer a solution of potassium iodide/iodate (Φ_{254} = 0.74)[3] was used, and as a secondary actinometer a solution of 2-phenylphenol (3 mg) in a mixture of solvents (2.25 mL of CH_3CN and 0.75 mL of D_2O) was used (Φ_{254} = 0.041).[4] The measurement was performed in triplicate (Table S3).

The number of the absorbed photons for the KIO_3/KI was calculated from:

$$n(\text{absorbed photons}) = \frac{\Delta A_{352} \cdot V_{\text{irr}}}{\varepsilon_{352} \cdot l \cdot \Phi_{\text{lit.}}} \qquad \text{(Eq. S3)}$$

where:

ΔA_{352} - absorbance difference at 352 nm for the irradiated and non-irradiated sample

V_{irr} - volume of the solution which was irradiated

ε_{352} - molar absorption coefficient for I_3^- in solution which contains iodides and iodates, 27600 M^{-1} cm^{-1}

l - length of the optical path (1 cm in all experiments)

$\Phi_{lit.}$ - quantum yield (Φ_{254} = 0.74), the precise value was calculated from:

$$c(\mathrm{I}^-) = \frac{A_{300}}{1.061} \quad [\mathrm{M}] \qquad \text{(Eq. S4)}$$

$$\Phi = 0.75 \cdot \left[1 + 0.02\left(\frac{T}{°\mathrm{C}} - 20.7\right)\right] \cdot \left[1 + 0.23\left(\frac{c(\mathrm{I}^-)}{\mathrm{M}} - 0.577\right)\right] \qquad \text{(Eq. S5)}$$

Determination of quantum yield with the solution of 2-phenylphenol as a secondary actinometer:

$$\Phi = \frac{\Delta I}{\Delta I_{\text{ref}}} \Phi_{\text{ref}} \qquad \text{(Eq. S6)}$$

where:

Φ_{ref} - quantum yield for 2-phenylphenol(Φ_{254} = 0.041)

ΔI - integral that corresponds to exchanged H-atoms at the position 2' (marked position) of the phenyl group

ΔI_{ref} - integral that corresponds to exchanged H-atoms at the position 2' (marked position) of the reference compound

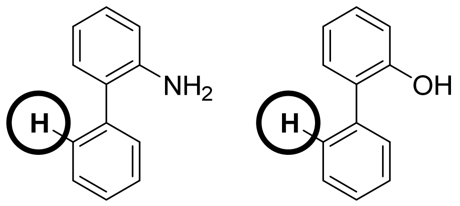

reference compound

Structure of the studied compound (left) and the reference compound (right).

Table S4. Quantum yields of deuterium exchange in 2-aminobiphenyl (**1**) measured by two different methods.

No. of Exp.	Primary Actinometer	Secondary Actinometer
1	0.0144±0.0003	0.014±0.005
2	0.023	0.018
3	0.012	0.016

Photolysis of 2-aminobiphenyl, dependence on water concentration.

2-Aminobiphenyl (**1**) (25 mg) was dissolved in CH_3CN (50 mL). Aliquot (10 mL) of the prepared solution was added to a quartz test tube (5 samples were prepared). To each of the test tubes, CH_3CN and solution of D_2SO_4 in D_2O (pD = 2.04) were added as it is shown in the Table below. The samples were purged with nitrogen for 15 min and irradiated in a Luzchem LZC-4X photochemical reactor (8 lamps, 300 nm) over 4 h. After the irradiation, the samples were diluted with saturated solution of $NaHCO_3$ (50 mL) and extracted with dichloromethane (3×25 mL). The combined organic layers were dried over sodium sulfate and concentrated in vacuum. The deuterium incorporation was determined by ^1H NMR and MS.

Table S5. Content of CH_3CN and D_2SO_4 in D_2O in the irradiated solutions of 2-aminobiphenyl

**Volume ratio (CH_3CN : D_2O)**	***V*(CH_3CN) / mL**	***V*(D_2SO_4/D_2O) / mL**
999:1	4.985	0.015
99:1	4.850	0.150
95:5	4.250	0.750
9:1	3.500	1.500
4:1	2.000	3.000

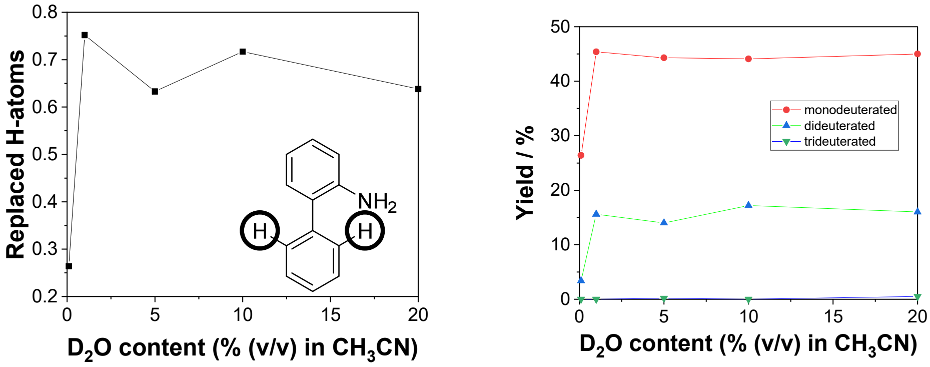

Figure S22. Dependence of the deuterium exchange at the position 2 of the phenyl group in **1** on D_2O content, determined by ^1H NMR (left) and the yield of mono- and dideuterated compound determined by MS (right). The irradiation was performed over 4 h, pD = 2.04.

Preparation and photolysis of deuterated 2-aminobiphenyl

2-Aminobiphenyl (**1**) (212 mg, 1.25 mmol) was dissolved in dry diethyl ether (10 mL). The solution was cooled to 0 °C and *n*-buthyllithium solution in hexane was added (V = 1 mL, c = 2.5 M, 2.50 mmol). The mixture was stirred for 30 min and after that D_2O was added (22.6 µL, 1.25 mmol). This process (addition of *n*-buthyllithium and deuterated water) was repeated two times. After the third addition of water, the mixture was concentrated in vacuum. The crude product was dissolved in dry dichloromethane and allowed to crystalize. The precipitate was filtered off. Again, the solvent was removed on a rotary evaporator and the crude product was dissolved in dry CH_3CN (20 mL). An aliquot (1 mL) of the prepared solution was added to a quartz test tube and diluted to 15 mL with dry CH_3CN. The solution was purged with nitrogen for 15 min and irradiated in a Luzchem LZC-4X photochemical reactor (8 lamps 300 nm lamps) over 16 h. After the photolysis, the sample was concentrated in vacuum and purified by chromatography on silica gel using dichloromethane as an eluent.

No deuterium incorporation could be observed by ^1H NMR and MS.

Photolysis of 2, dependence on water solution pD.

2-Aminobiphenyl (**1**) (45 mg) was dissolved in CH_3CN (90 mL). An aliquot (10 mL) of the prepared solution was added to a quartz test tube (9 samples were prepared). Each of the sample was prepared by addition of 5 mL of the following solutions in Table S5. The samples were purged with nitrogen for 15 min and irradiated in a Luzchem LZC-4X photochemical reactor (8 lamps, 300 nm) over 4 h. After the irradiation, the samples were diluted with saturated

solution of $NaHCO_3$ (50 mL) and extracted with dichloromethane (3×25 mL). The combined organic layers were dried over sodium sulfate and concentrated in vacuum. The deuterium incorporation was determined by ^{1}H NMR and MS.

Table S6. pD values and content of the irradiated solutions of 2-aminobiphenyl

Sample	pD
**1.**	0.96 (solution of D_3PO_4 in D_2O)
**2.**	1.68 (solution of D_3PO_4 in D_2O)
**3.**	2.24 (solution of D_3PO_4 in D_2O)
**4.**	3.02 (deuterated phosphate buffer)
**5.**	3.95 (deuterated phosphate buffer)
**6.**	6.15 (deuterated phosphate buffer)
**7.**	7.00 (deuterated phosphate buffer)
**8.**	8.08 (deuterated phosphate buffer)
**9.**	10.05 (deuterated phosphate buffer)

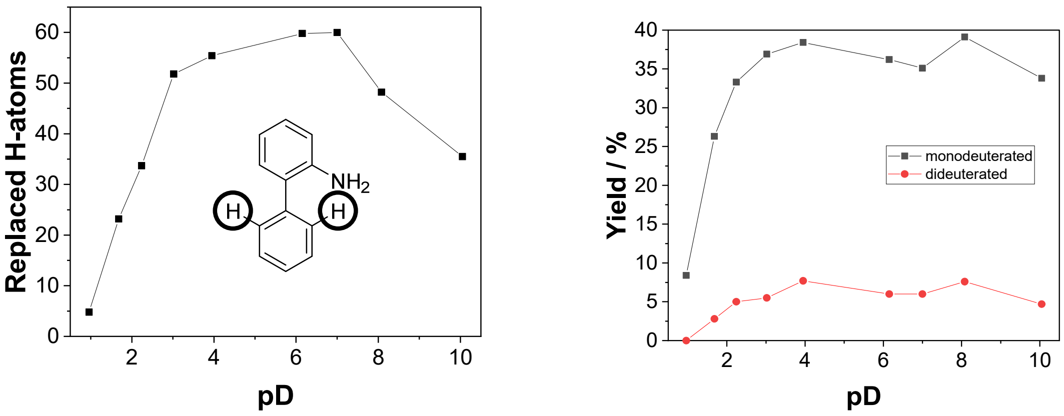

Figure S23. Dependence of the deuterium exchange at the position 2' (marked position) of the phenyl group in **1** on pD, determined by ^{1}H NMR (left) and the yield of mono- and dideuterated compound determined by MS (right). The irradiation was performed over 4 h.

Photolysis of *N*,*N*-dimethyl-2-aminobiphenyl.

N,*N*-dimethyl-2-aminobiphenyl (5 mg) was dissolved in CH_3CN (10 mL). The solution was transferred to a quartz test tube and the sample was diluted by a solution of D_2SO_4 in D_2O (5 mL, pD = 2.05). The sample was purged with nitrogen for 15 min and irradiated in a Luzchem LZC-4X photochemical reactor (8 lamps, 300 nm) over 4 h. After the irradiations, the sample was diluted with a saturated solution of $NaHCO_3$ (50 mL) and extracted with dichloromethane

(3×25 mL). The combined organic layers were dried over sodium sulfate and concentrated in vacuum. Deuterium incorporation was determined by ^{1}H NMR. Under the same condition, irradiation of nonmethylated 2-aminobiphenyl (5 mg) was performed. The control experiment in the dark was performed with both compounds (methylated and nonmethylated) at the same concentrations and in the presence of the sulfuric acid, pD = 2.

Table S7. Deuterium exchange (replaced H-atoms) at the position 2' of the phenyl group for the methylated and nonmethylated 2-aminobiphenyl, determined by ^{1}H NMR. The irradiation was performed over 4 h.

Compound	Irradiation	Control exp.
**2-aminobiphenyl (1)**	1.166	0
***N*,*N*-dimethyl-2-aminobiphenyl 1(NMe$_2$)**	0.075	0.007

Time dependence

3-aminobiphenyl (**2**) (25 mg) was dissolved in CH_3CN (50 mL). An aliquot (10 mL) of the prepared solution was added to a quartz test tube and the prepared sample was diluted by a solution of D_2SO_4 in D_2O (5 mL, pD = 2.03). By this way 5 samples were prepared. Each sample was purged with nitrogen for 15 min and irradiated in a Luzchem LZC-4X photochemical reactor (8 lamps, 300 nm) over different time. After the irradiations, the samples were diluted with a saturated aqueous solution of $NaHCO_3$ (50 mL) and extracted with dichloromethane (3×25 mL). The combined organic layers were dried over sodium sulfate and concentrated in vacuum. Deuterium incorporation was determined by ^{1}H NMR.

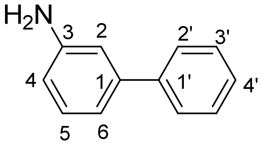

3-aminobiphenyl (**2**) ^{1}H NMR (600 MHz, $(CD_3)_2CO$): δ/ppm 7.57 (dd, 2H, J = 1.2 Hz, J = 7.5 Hz, H-2'), 7.41 (dd (t), 2H, J = 7.5 Hz, H-3'), 7.31 (tt, 1H, J = 1.2 Hz, J = 7.5 Hz, H-4'), 7.13 (dd, 1H, J = 7.3 Hz, J = 7.8 Hz, H-5), 6.95 (dd (t), 1H, J = 1.8 Hz, H-2), 6.86 (dd, 1H, J = 1.8 Hz, J = 7.5 Hz, H-6), 6.66 (dd, 1H, J = 1.8 Hz, J = 7.5 Hz, H-4).

Upon irradiation, the intensity of the signal at δ 6.66 ppm corresponding to H-4 is reduced (see Fig S24).

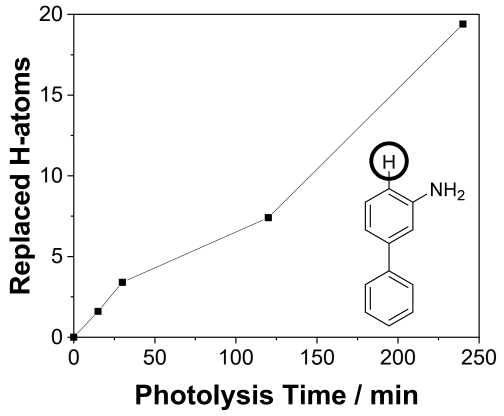

Figure S24. Dependence of the deuterium exchange at the position 4 of the aniline in 3-aminobiphenyl (**2**) on photolysis time, determined by ^{1}H NMR. The irradiation was performed in D_2O-CH_3CN (1:2).

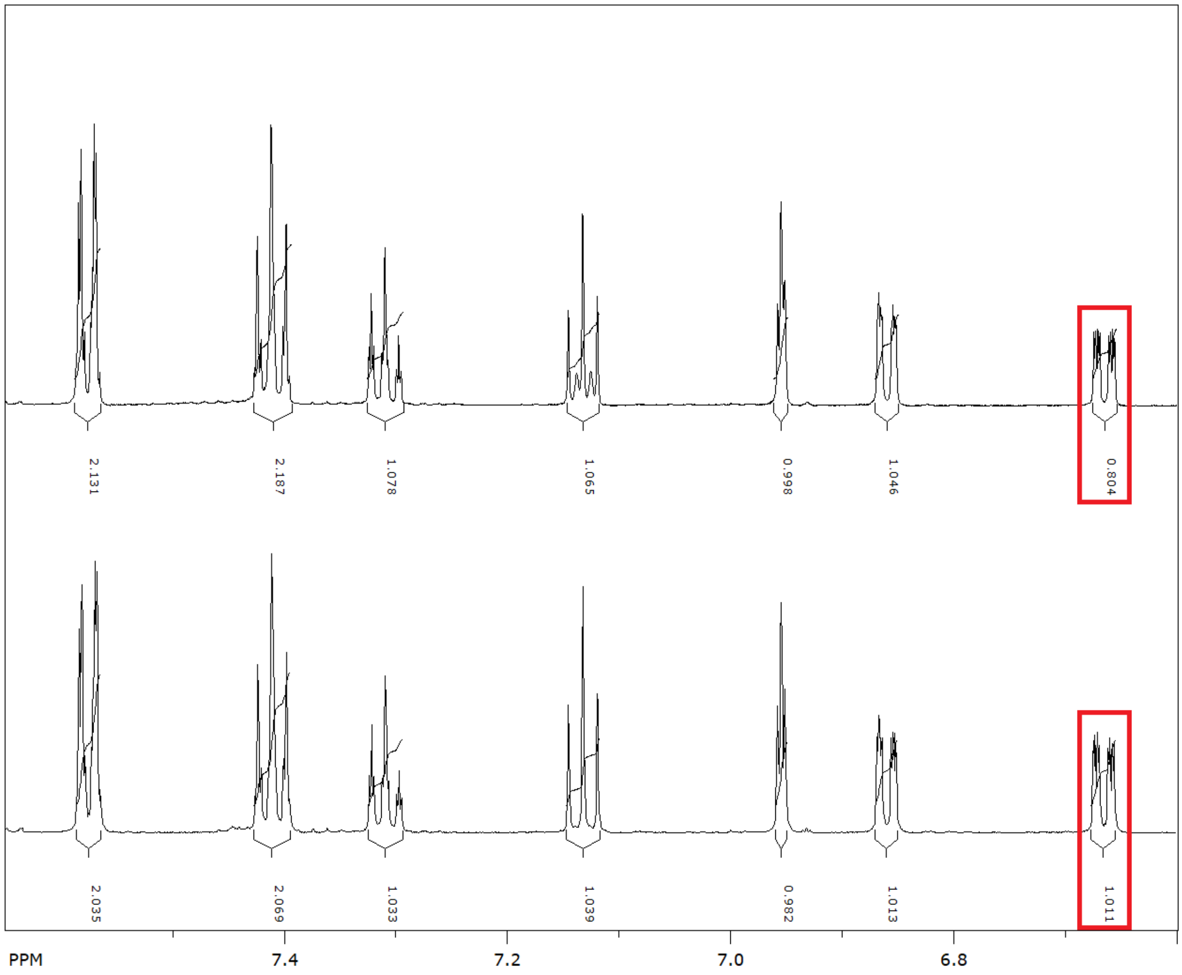

Figure S25. Expanded aromatic region of the ^{1}H NMR ($(CD_3)_2CO$, 600 MHz) spectra of 3-aminobiphenyl (**2**) before (bottom) and after (top) the photolysis (4 h, 300 nm) in D_2O-CH_3CN (1:2).

Preparative irradiation of 3-aminobiphenyl.

A solution of 3-aminobiphenyl (**2**) (100 mg) in a mixture of acetonitrile (80 mL) and a solution of H_2SO_4 in water (20 mL) was transferred to a quartz vessel equipped with a stirring magnet. The sample was purged with nitrogen for 30 min and irradiated in a Luzchem LZC-4X photochemical reactor (8 lamps, 300 nm) over 12 h. After the photolysis, the sample was concentrated in vacuum and purified by chromatography on silica gel using dichloromethane as an eluent. According to TLC, only one product was obtained in this reaction. ^{1}H NMR spectrum of this product is given below:

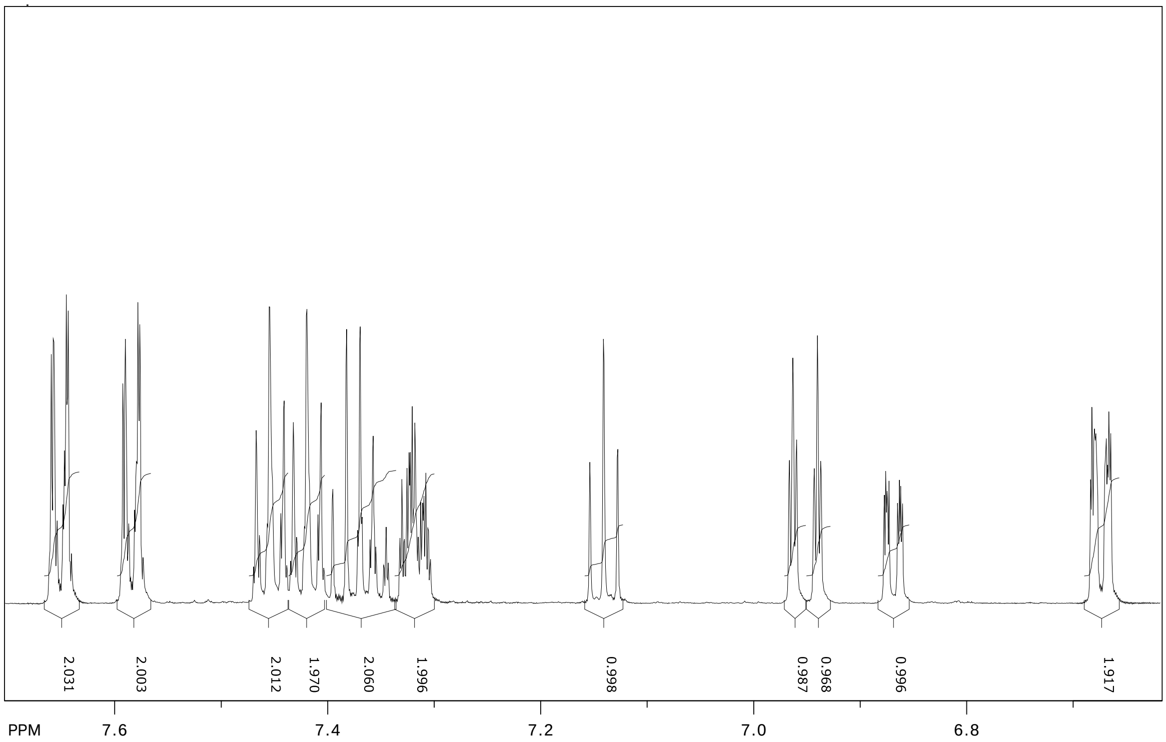

Figure S26. Expanded aromatic region of the ^{1}H NMR spectrum ($(CD_3)_2CO$, 600 MHz) of **2** after the photolysis (pD = 2, 12 h, 300 nm) in H_2O-CH_3CN (1:4) and chromatography. Photoproduct and reactant are present in a ratio of 1: 1.

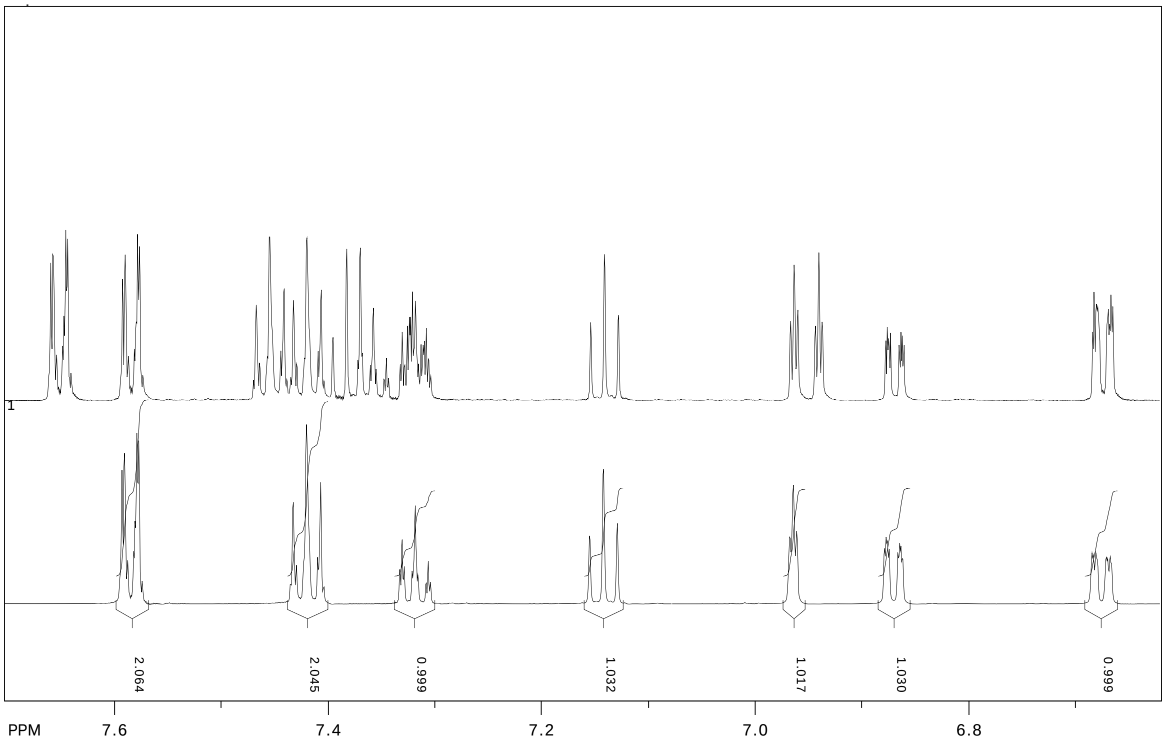

Figure S27. Expanded aromatic region of the ^{1}H NMR ($(CD_3)_2CO$, 600 MHz) spectra of **2** before (bottom) and after (top) the photolysis (pD = 2, 12 h, 300 nm) in H_2O-CH_3CN (1:4).

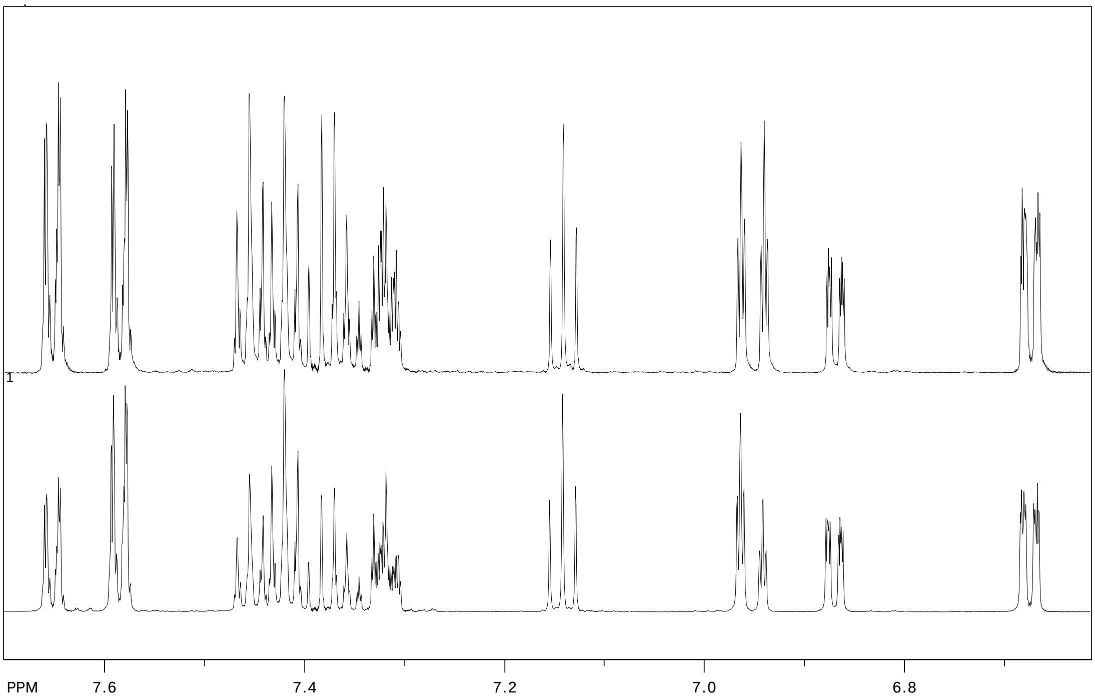

Figure S28. Expanded aromatic region of the ^{1}H NMR spectra ($(CD_3)_2CO$, 600 MHz) after the photolysis of **2** (top) and after reduction of 3-nitrobiphenyl with hydrazine monohydrate and activated Raney nickel (bottom).

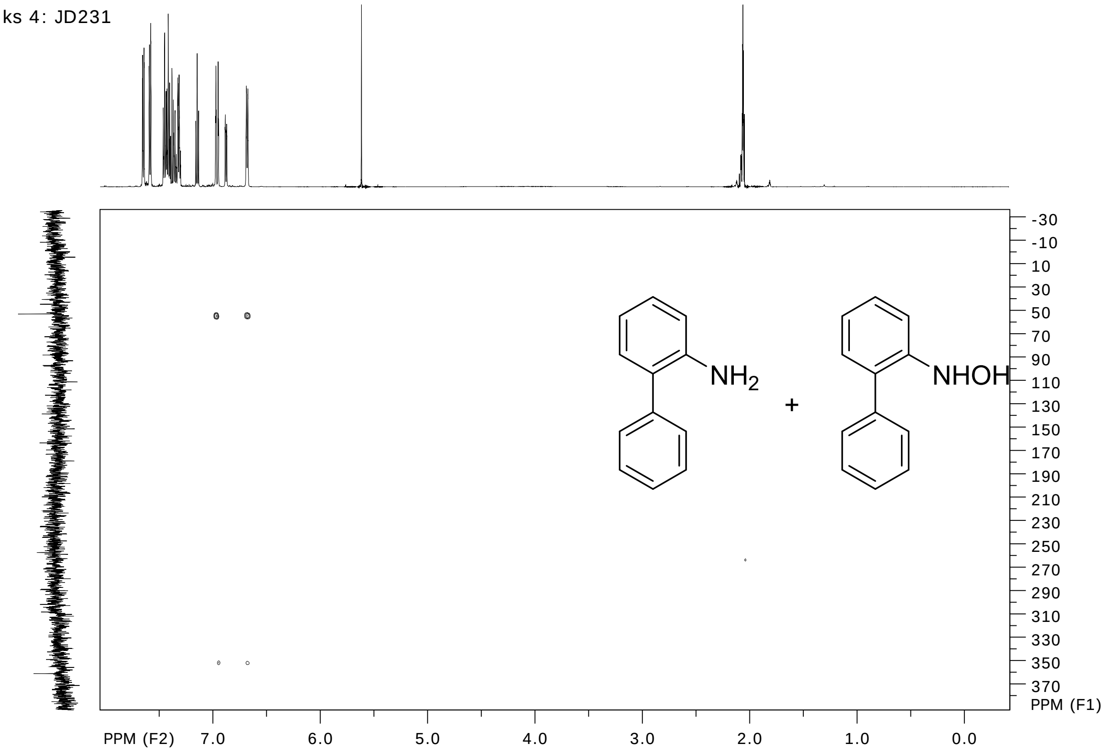

Figure S29. ^{1}H-^{15}N HMBC NMR spectrum ($(CD_3)_2CO$, ^{1}H NMR 600 MHz, ^{15}N NMR 61 MHz) of **2** after the photolysis (pD = 2, 12 h, 300 nm) in H_2O-CH_3CN (1:4) and chromatography.

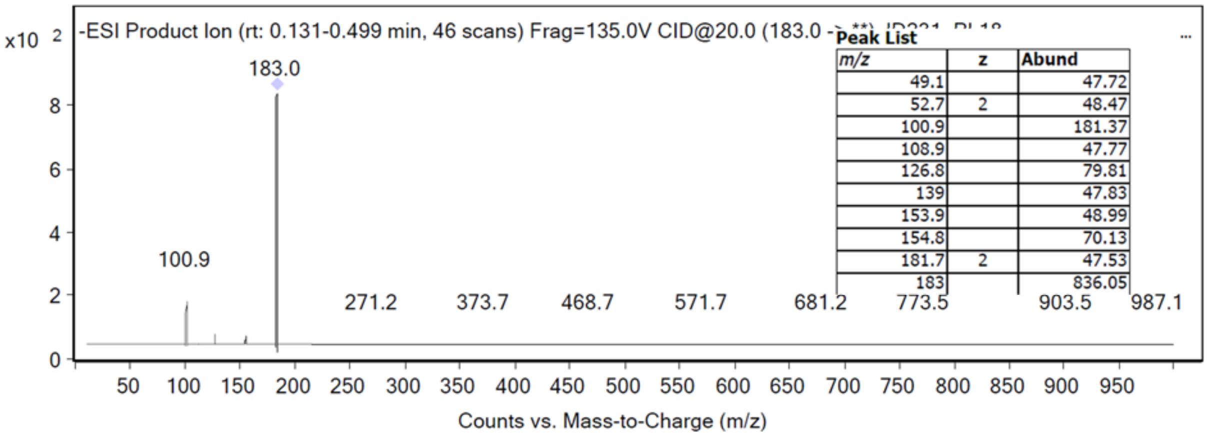

Figure S30. ESI-MS-MS spectrum (peak 183 in negative mode) of **2** after the photolysis (pD = 2, 12 h, 300 nm) in H_2O-CH_3CN (1:4) and chromatography.

Peak at 155 indicates that water could be one of the fragmentation products.

Reduction of 3-nitrobiphenyl

To the solution of 3-nitrobiphenyl (100 mg, 0.502 mmol) in a mixture of ethanol (1 mL) and dichloroethane (1 mL) suspension of activated Raney nickel (100 mg) in ethanol (1.5 mL) was

added at 0 °C under an argon atmosphere. After that, a mixture of hydrazine monohydrate and water ($w(N_2H_4 \times H_2O)$ = 80 %, 200 μL) was added dropwise (at 0 °C). The mixture was stirred until the TLC indicated the complete consumption of the starting material. The reaction mixture was filtered and the solvent was removed on a rotary evaporator. According to TLC, only one product was obtained in this reaction. ^{1}H NMR spectrum of this crude product is given below:

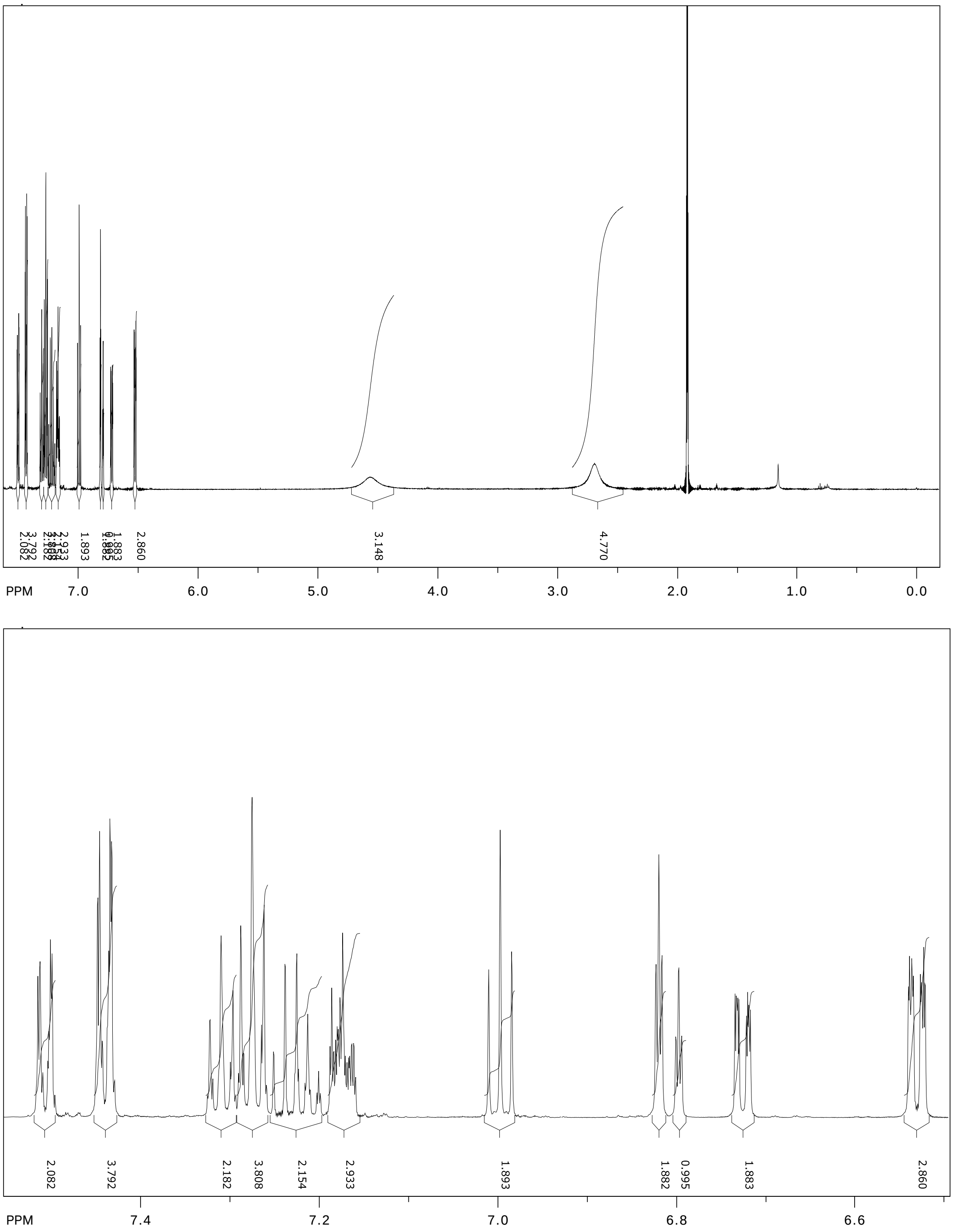

Figure S31. ^{1}H NMR ($(CD_3)_2CO$, 600 MHz) of a mixture containing **2** and **4** after the reduction of nitrobiphenyl; bottom panel is enlarged aromatic region of the spectrum.

Time dependence

4-aminobiphenyl (**3**) (35 mg) was dissolved in CH_3CN (70 mL). An aliquot (10 mL) of the prepared solution was added to a quartz test tube and the prepared sample was diluted by a solution of D_2SO_4 in D_2O (5 mL, pD = 1.99). By this way 7 samples were prepared. Each sample was purged with nitrogen for 15 min and irradiated in a Luzchem LZC-4X photochemical reactor (8 lamps, 300 nm) over different time. After the irradiations, the samples were diluted with a saturated solution of $NaHCO_3$ (50 mL) and extracted with dichloromethane (3×25 mL). The combined organic layers were dried over sodium sulfate and concentrated in vacuum. Deuterium incorporation was determined by MS.

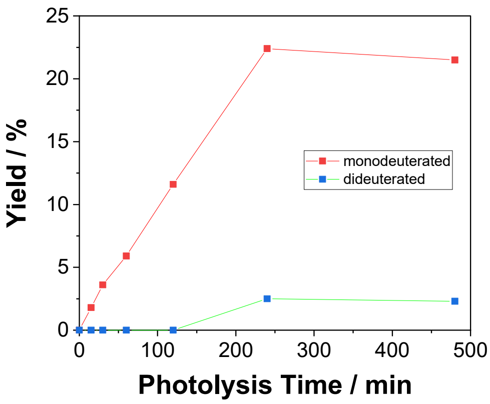

Figure S32. Dependence of the yield of mono- and dideuterated **3** on photolysis time, determined by MS. The irradiation was performed in D_2O-CH_3CN (1:2).

Photolysis of 4-aminobiphenyl, dependence on water solution pD.

4-Aminobiphenyl (**3**) (30 mg) was dissolved in CH_3CN (60 mL). An aliquot (10 mL) of the prepared solution was added to a quartz test tube (6 samples were prepared). Each of the sample was prepared by addition of 5 mL of the following solutions in Table S6. The samples were purged with nitrogen for 15 min and irradiated in a Luzchem LZC-4X photochemical reactor (8 lamps, 300 nm) over 4 h. After the irradiation, the samples were diluted with saturated solution of $NaHCO_3$ (50 mL) and extracted with dichloromethane (3×25 mL). The combined organic layers were dried over sodium sulfate and concentrated *in vacuum*. The deuterium incorporation was determined by ^{1}H NMR and MS.

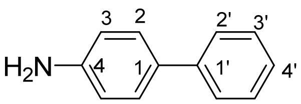

4-aminobiphenyl (**3**) ^{1}H NMR (600 MHz, CD_3OD): δ/ppm 7.51 (dd, 2H, J = 1.2 Hz, J = 8.3 Hz, H-2'), 7.37 (d, 2H, J = 8.6 Hz, H-2), 7.35 (dd, 2H, J = 7.3 Hz, J = 8.3 Hz, H-3'), 7.21 (tt, 1H, J = 1.1 Hz, J = 7.3 Hz, H-4'), 6.79 (d, 2H, J = 8.6 Hz, H-3).

Upon irradiation, the intensity of the signal at δ 7.51 ppm corresponding to H-2' is reduced (see Fig S33).

Table S8. pD values and content of the irradiated solutions of **3**.

Sample	pD
**1.**	0.80 (solution of D_3PO_4 in D_2O)
**2.**	1.96 (solution of D_3PO_4 in D_2O)
**3.**	2.98 (deuterated phosphate buffer)
**4.**	5.00 (deuterated phosphate buffer)
**5.**	7.07 (deuterated phosphate buffer)
**6.**	9.04 (deuterated phosphate buffer)

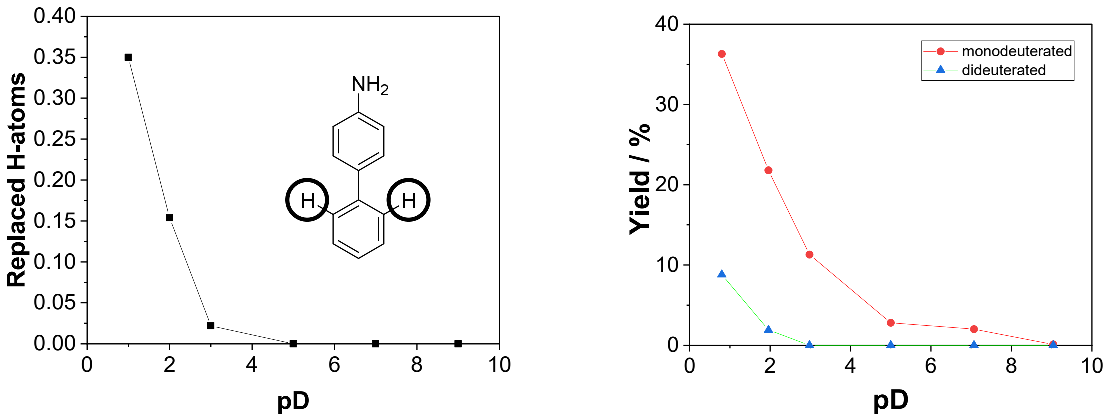

Figure S33. Dependence of the deuterium exchange at the position 2' (marked position) of the phenyl group **3** on pD, determined by ^{1}H NMR (left) and the yield of mono- and dideuterated compound determined by MS (right). The irradiation was performed over 4 h.

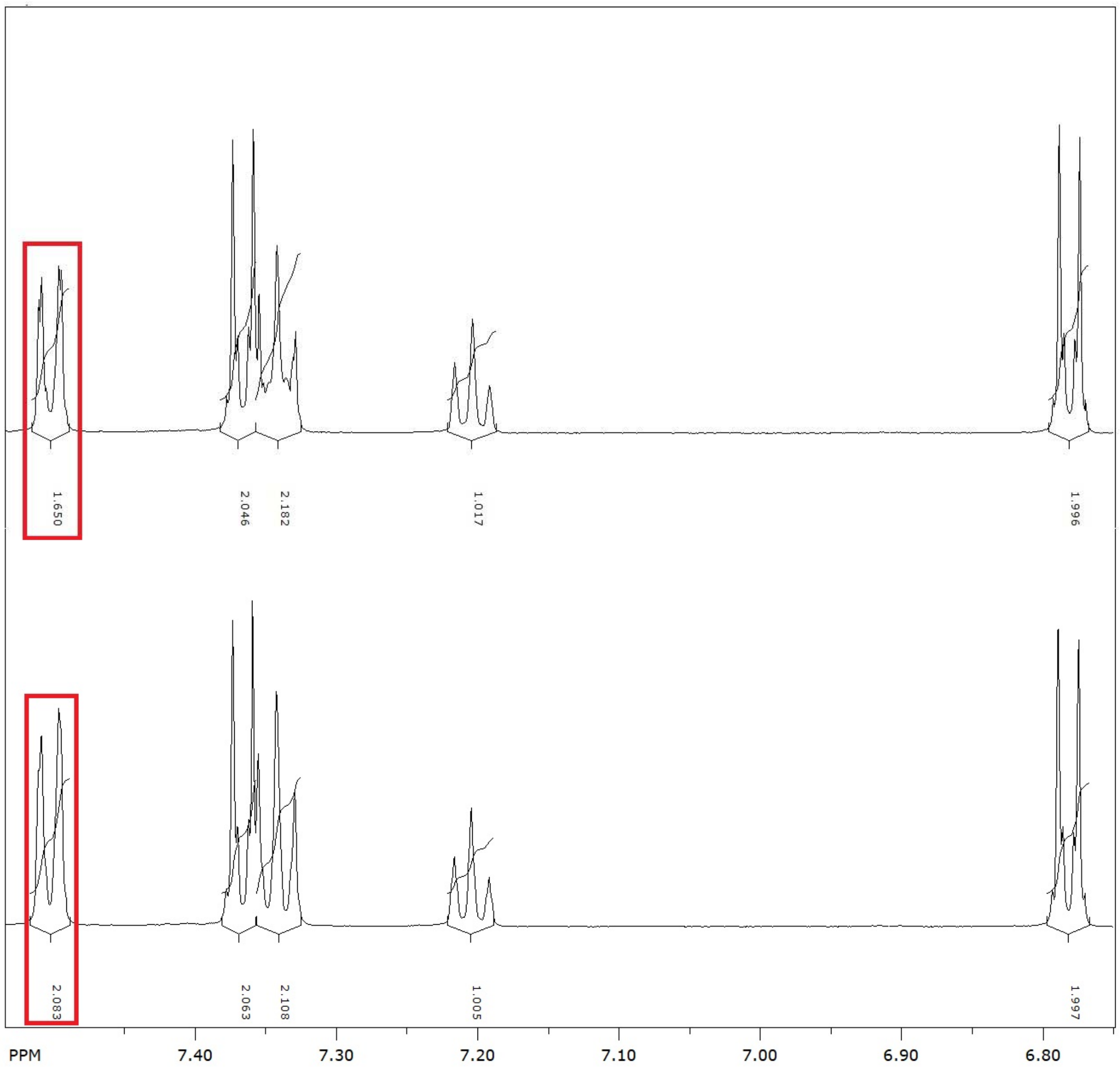

Figure S34. Expanded aromatic region of the ^{1}H NMR (CD_3OD, 600 MHz) spectra of **3** before (bottom) and after (top) the photolysis (pD = 1, 4 h, 300 nm) in D_2O-CH_3CN (1:2).

Irradiation under N_2 and O_2

Aminobiphenyl 1, **2**, or **3** (5 mg) was dissolved in CH_3CN (10 mL) and poured to a quartz test tube, to which a solution of D_2SO_4 in D_2O (5 mL, pD = 1.94) was added. In this way 6 solutions were prepared, two per compound. Three solutions were purged with nitrogen for 15 min and the other three were purged with O_2. The test tubes were irradiated at the same time in a Luzchem LZC-4X photochemical reactor (8 lamps, 300 nm) over 3h. After the irradiations, the samples were diluted with a saturated aqueous solution of $NaHCO_3$ (50 mL) and extracted with dichloromethane (3×25 mL). The combined organic layers were dried over sodium sulfate and

concentrated in vacuum. Deuterium incorporation was determined by ^{1}H NMR. The extent of D-incorporation and yield of the photo-redox product are given in Table S9.

Table S9. Content of D-exchange and photo-redox yield.[a]

	N_2-purged	O_2-purged
**1**	52% D	12% D
**2**	19% D, 55% photo-redox	11% D, 0% photo-redox
**3**	5% D	5% D

[a] Determined from ^{1}H NMR.

5. Laser Flash Photolysis

Stock solutions of 2-aminobiphenyl (**1**, 2.81 mg) and 3-aminobiphenyl (**2**, 0.78 mg) were prepared in CH_3CN (25 mL). An aliquot (5 mL) of the prepared solution was added to a volumetric flask (2 samples). 2 samples were prepared: in the first flask previously added solution was diluted with CH_3CN to 25 mL; the second sample was prepared by addition of pure water (6.25 mL) and after that diluted to 25 mL with CH_3CN. The final concentration for all samples was 1.33×10^{-4} M, corresponding to the absorbances at 266 nm A = 0.30. The measurements were performed on a LP980 Edinburgh Instruments spectrophotometer. For the excitation the fourth harmonic of a Q-smart Q450 Quantel YAG laser at 266 nm was used. The energy of the laser pulse at 266 nm was set to ≈ 20 mJ and the pulse duration was 7 ns. Absorbances at the excitation wavelength were set to 0.3. The static cells were used and they were frequently exchanged to assure no absorption of light by photoproducts. The solutions were purged by Ar or O_2 for 15 min prior to the measurements, which were conducted at 25 °C.

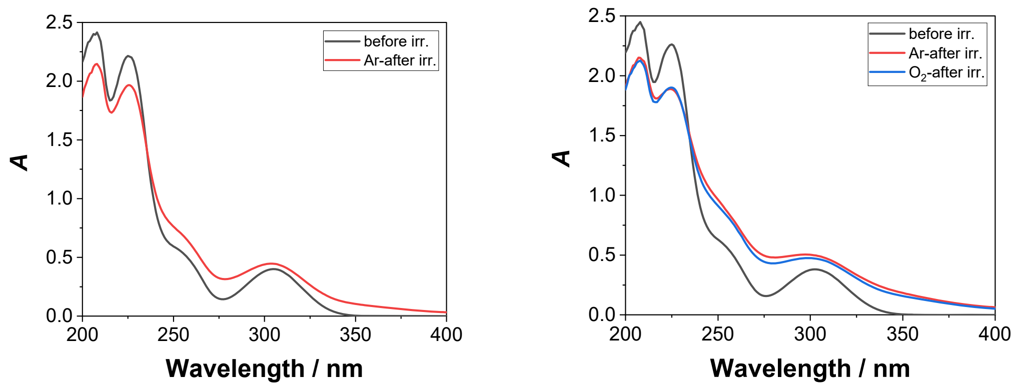

Figure S35. Absorption spectrum of 2-aminobiphenyl (**1**) in CH_3CN (left) and in CH_3CN-H_2O (4:1, right) before and after the LFP experiment.

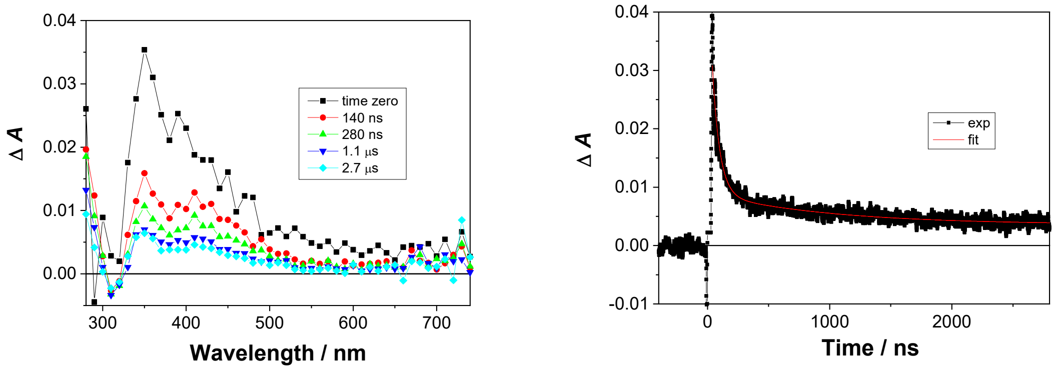

Figure S36. Transient absorption spectra of 2-aminobiphenyl (**1**, *c* = 1.33×10^{-4} M) in Ar-purged CH_3CN, A_{266} = 0.3, laser pulse 20 mJ, and decay of the transient at 420 nm.

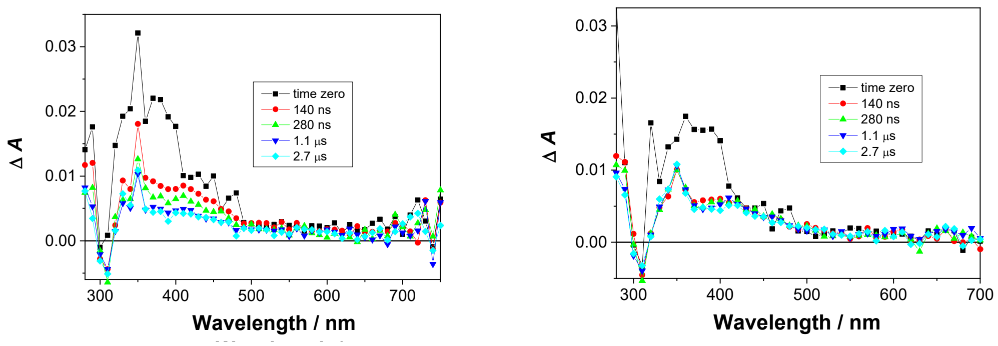

Figure S37. Transient absorption spectra of 2-aminobiphenyl (**1**, *c* = 1.33×10^{-4} M) in Ar-purged (left) and O_2-purged (right) CH_3CN-H_2O (4:1), A_{266} = 0.3, laser pulse 20 mJ.

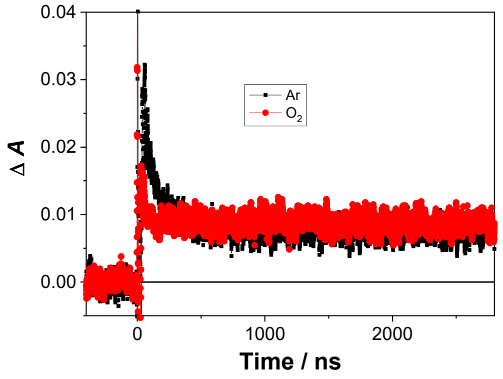

Figure S38. Decay of transient absorption at 360 nm for Ar- and O_2-purged CH_3CN-H_2O (4:1) solution of **1** (*c* = 1.33×10^{-4} M).

In the Ar-purged CH_3CN solution of **1**, a transient was detected that absorbed at 300-500nm, with a maximum at ≈ 350 nm. It decayed with the lifetime of τ = 130 ± 30 ns. In addition, a

long-lived transient was detected that absorbed also at 300-500 nm, whose intensity was too low to get its decay kinetics precisely.

In the Ar-purged CH_3CN-H_2O (4:1), a similar transient was detected that decayed with the lifetime of τ = 110 ± 10 ns, and a long-lived transient that decayed in the millisecond time-scale whose decay kinetics could not be precisely determined. In the O_2-purged solution, the short-lived transient was quenched and its decay was at the limits of detection, comparable to the duration of the laser pulse. The long-lived is probably not quenched by O_2.

Based on the quenching by O_2 and, the short-lived transient was assigned to the triplet-triplet absorption. The assignment is in accord with the published triplet-triplet absorption spectra of aniline[5] or *p*-phenylenediamine.[6] The long-lived transient was tentatively assigned to N-centred radical formed by H-atom detachment, which is an ubiquitous process in the photochemistry of triplet aniline derivatives.[7] The assignment is based on the published spectra of similar N-radical species.[8,9,10] The anticipated short-lived aza-QM was not detected, due to its low quantum yield for the formation. Note that it is anticipated to have a short lifetime, in accord with similar aza-quinone methide[11] and quinone methide species formed in the ESIPT to carbon.[12]

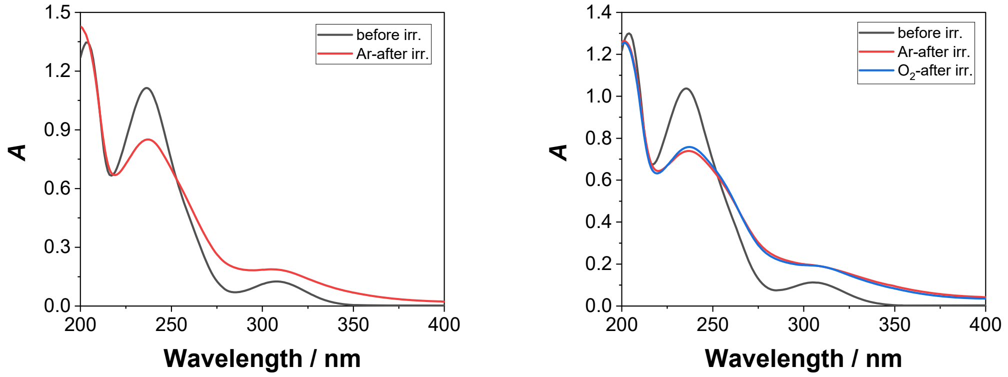

Figure S39. Absorption spectrum of 3-aminobiphenyl (**2**) in CH_3CN (left) and in CH_3CN-H_2O (4:1, right) before and after the LFP experiment.

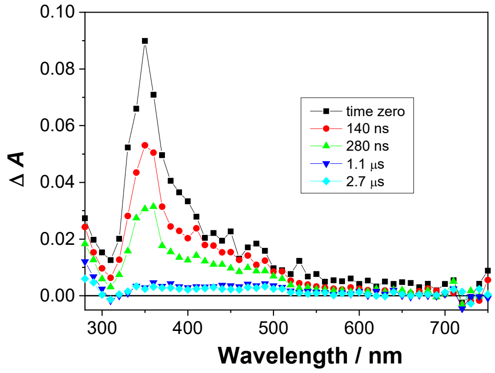

Figure S40. Transient absorption spectra of 3-aminobiphenyl (**2**, $c = 3.68\times10^{-5}$ M) in Ar-purged CH_3CN, $A_{266} = 0.3$, laser pulse 20 mJ.

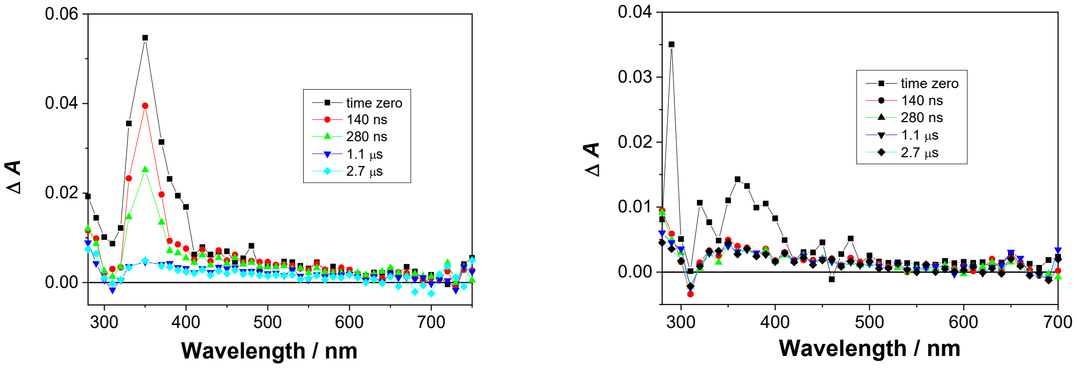

Figure S41. Transient absorption spectra of 3-aminobiphenyl (**2**, $c = 3.68\times10^{-5}$ M) in Ar-purged (left) and O_2-purged (right) CH_3CN-H_2O (4:1), $A_{266} = 0.3$, laser pulse 20 mJ.

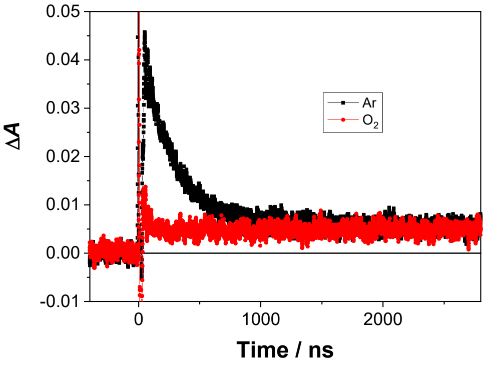

Figure S42. Decay of transient absorption of **2** at 360 nm for Ar- and O_2-purged CH_3CN-H_2O (4:1) solution of 3-aminobiphenyl ($c = 3.68\times10^{-5}$ M).

In the Ar-purged CH_3CN solution of the *m*-compound, a transient was detected that absorbed strongly at 300-500 nm, with a maximum at ≈ 350 nm, and decayed with the lifetime of τ = 120 ± 10 ns. In addition, a long-lived transient was detected that absorbed at 350-550 nm, whose intensity was too low to get its decay kinetics precisely.

In the Ar-purged CH_3CN-H_2O (4:1), a similar transient was detected that decayed somewhat slower with the lifetime of τ = 250 ± 20 ns, and a long-lived transient that decayed in the millisecond time-scale whose decay kinetics could not be precisely determined. In the O_2-purged solution, the short-lived transient was quenched and it decayed with τ = 30 ± 10 ns. The long-lived is probably not quenched by O_2.

Based on the quenching by O_2 and comparison with some published spectra of aniline triplet excited states,[5,6] the short-lived transient was assigned to the triplet-triplet absorption. The long-lived transient was tentatively assigned to a N-centred radical, based on the comparison with the published spectra,[8,9,10] but due to its low intensity of the transient absorption further studies were precluded. The anticipated radical-cation and solvated electrons were not detected.

6. Computations

Table S10. ADC(2) vertical excitation energies and oscillator strengths (in parentheses) of the four lowest excited states of 2-aminobiphenyl (**1**) in vacuo and CH_3CN solution approximated with the COSMO solvation model.

	In vacuo		COSMO	
	cc-pVDZ	aug-cc-pVDZ	cc-pVDZ	aug-cc-pVDZ
S_1	4.64 (0.0752)	4.44 (0.0740)	4.60 (0.1010)	4.45 (0.0940)
S_2	5.11 (0.0024)	4.73 (0.0042)	5.10 (0.0020)	4.97 (0.0041)
S_3	5.47 (0.0575)	4.96 (0.0056)	5.44 (0.0946)	5.03 (0.0034)
S_4	5.84 (0.0239)	5.13 (0.0285)	5.96 (0.1198)	5.18 (0.0775)
S_4	6.01 (0.1485)	5.32 (0.0288)	6.05 (0.4455)	5.48 (0.0399)

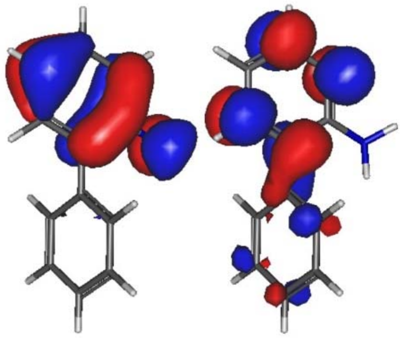

$S_0 \leftarrow S_1$ (87%)

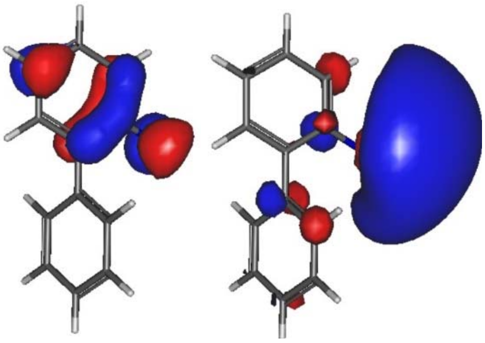

$S_0 \leftarrow S_2$ (99%)

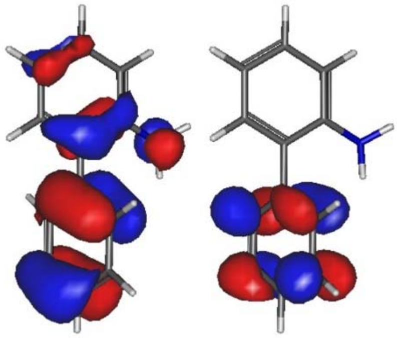

$S_0 \leftarrow S_3$ (62%)

Figure S43. NTO orbitals for the first three singlet excited states for **1** computed at the ADC(2)/aug-cc-pVDZ level of theory. The dominant hole-particle pairs are shown only, the contribution is given in parenthesis. Note that at the ADC(2)/aug-cc-pVDZ level, the S_3 state becomes the S_2 state.

Table S11. ADC(2) vertical excitation energies and oscillator strengths (in parentheses) of the four lowest excited states of 3-aminobiphenyl (**2**) in vacuo and CH_3CN solution approximated with the COSMO solvation model.

	In vacuo		COSMO	
	cc-pVDZ	aug-cc-pVDZ	cc-pVDZ	aug-cc-pVDZ
S_1	4.60<br>(0.0642)	4.40<br>(0.0671)	4.56<br>(0.0825)	4.36<br>(0.0880)
S_2	5.05<br>(0.0001)	4.78<br>(0.0042)	5.06<br>(0.0001)	4.91<br>(0.0002)
S_3	5.51<br>(0.2606)	4.92<br>(0.0002)	5.46<br>(0.2816)	5.05<br>(0.0069)
S_4	5.92<br>(0.7342)	5.24<br>(0.1720)	5.81<br>(0.8706)	5.15<br>(0.1915)
S_4	6.32<br>(0.0322)	5.39<br>(0.0036)	6.34<br>(0.0967)	5.47<br>(0.8864)

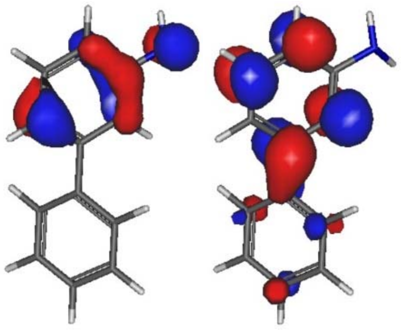

$S_0 \leftarrow S_1$ (85%)

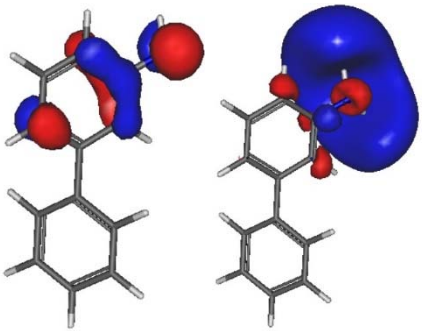

$S_0 \leftarrow S_2$ (99%)

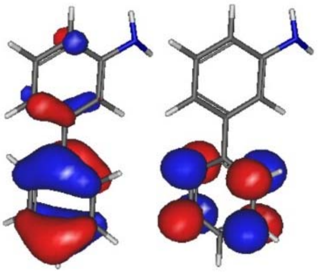

$S_0 \leftarrow S_3$ (55%)

Figure S44. NTO orbitals for the first three singlet excited states for **2** computed with ADC(2)/aug-cc-pVDZ. The dominant hole-particle pairs are shown only, the contribution is given in parenthesis. As in **1**, at the ADC(2)/cc-pVDZ level, the S_1 state is unaffected, the Rydberg state is destabilized, while the S_3 state at the ADC(2)/aug-cc-pVDZ level becomes the S_2 state.

Table S12. ADC(2) vertical excitation energies and oscillator strengths (in parentheses) of the four lowest excited states of 4-aminobiphenyl (**3**) in vacuo and CH_3CN solution approximated with the COSMO solvation model.

	In vacuo		COSMO	
	cc-pVDZ	aug-cc-pVDZ	cc-pVDZ	aug-cc-pVDZ
S_1	4.67 (0.0230)	4.44 (0.0210)	4.64 (0.0289)	4.42 (0.0263)
S_2	5.00 (0.0014)	4.63 (0.0342)	4.94 (0.8198)	4.70 (0.7410)
S_3	5.09 (0.7312)	4.83 (0.3028)	4.99 (0.0024)	4.79 (0.0048)
S_4	6.28 (0.0583)	4.85 (0.3005)	6.20 (0.0710)	4.93 (0.0008)
S_4	6.36 (0.0030)	5.19 (0.0045)	6.32 (0.0102)	5.36 (0.0036)

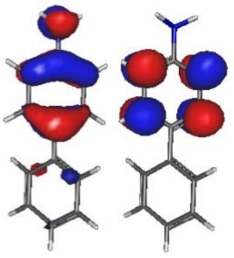

$S_0 \leftarrow S_1$ (84%)

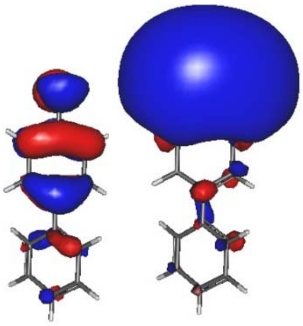

$S_0 \leftarrow S_2$ (98%)

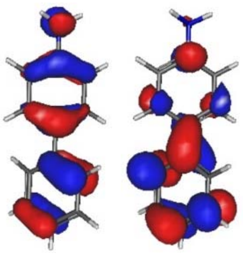

$S_0 \leftarrow S_3$ (81%)

Figure S45. NTO orbitals for the first three singlet excited states for **3** computed at the ADC(2)/aug-cc-pVDZ level of theory. The dominant hole-particle pairs are shown only, the contribution is given in parenthesis. As in **1** and **2**, at the ADC(2)/cc-pVDZ level, the S_1 state is unaffected, the Rydberg state is destabilized, while the S_3 state at the ADC(2)/aug-cc-pVDZ level becomes the S_2 state.

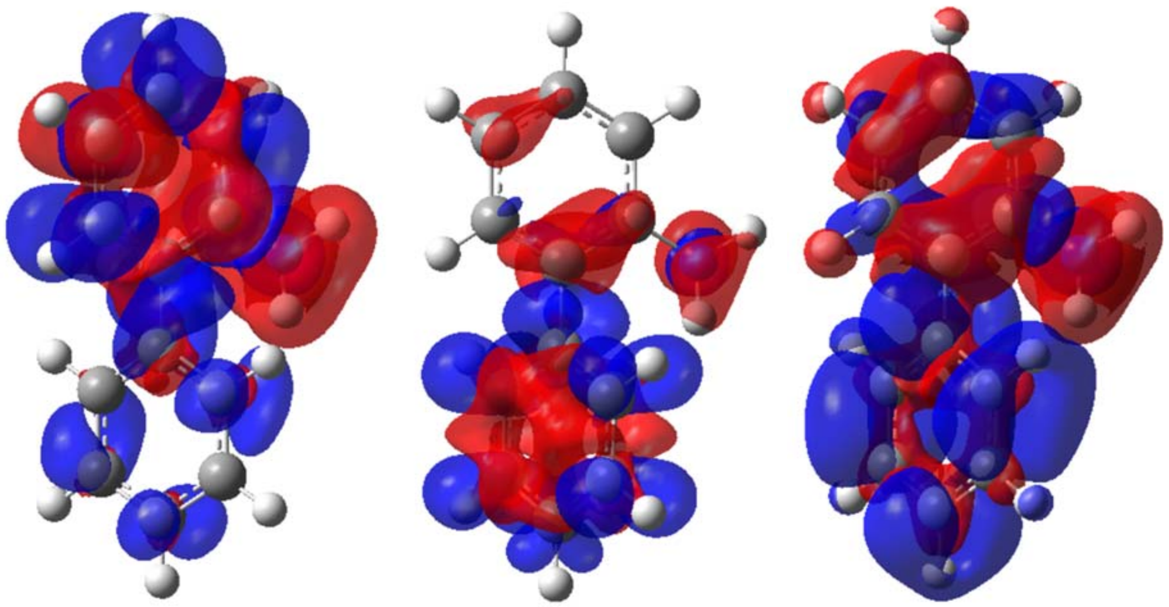

Figure S46. Projection of the electron density difference of the S_1 state (left), S_2 state (middle) and S_3 state (right) on the electron isodensity surface of the ground state, S_0 for **1**. Areas of increase (depletion) of electron density in the excited states are shown in blue (red). The calculations were performed with the ADC(2)/cc-pVDZ method.

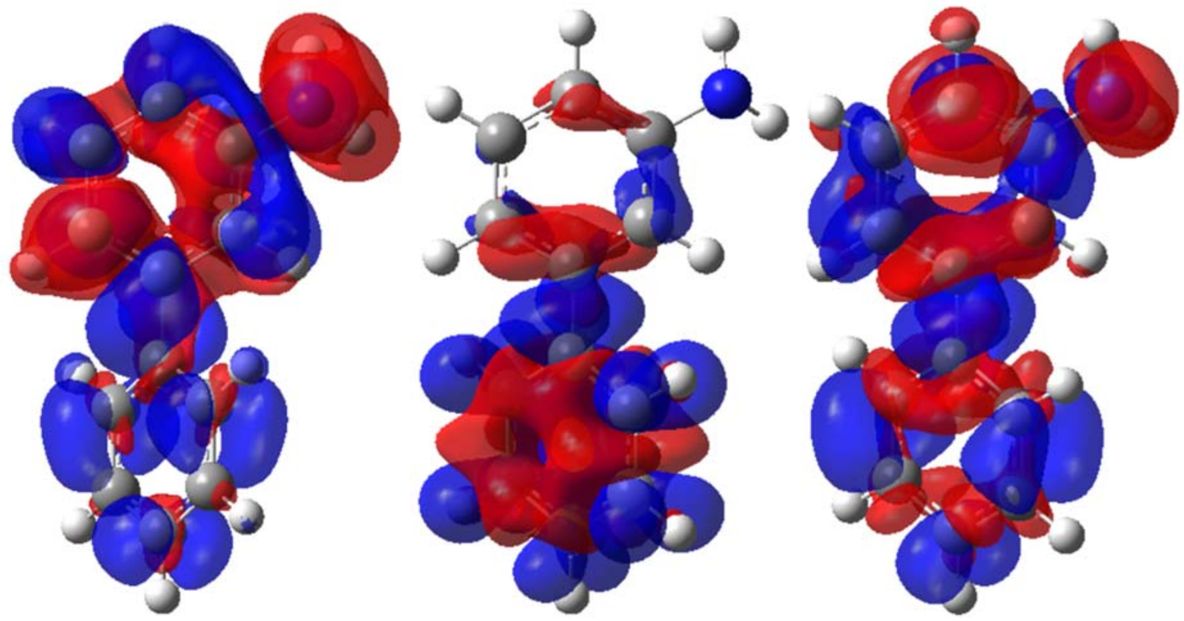

Figure S47. Projection of the electron density difference of the S_1 state (left), S_2 state (middle) and S_3 state (right) on the electron isodensity surface of the ground state, S_0 for **2**. Areas of increase (depletion) of electron density in the excited states are shown in blue (red). The calculations were performed with the ADC(2)/cc-pVDZ method.

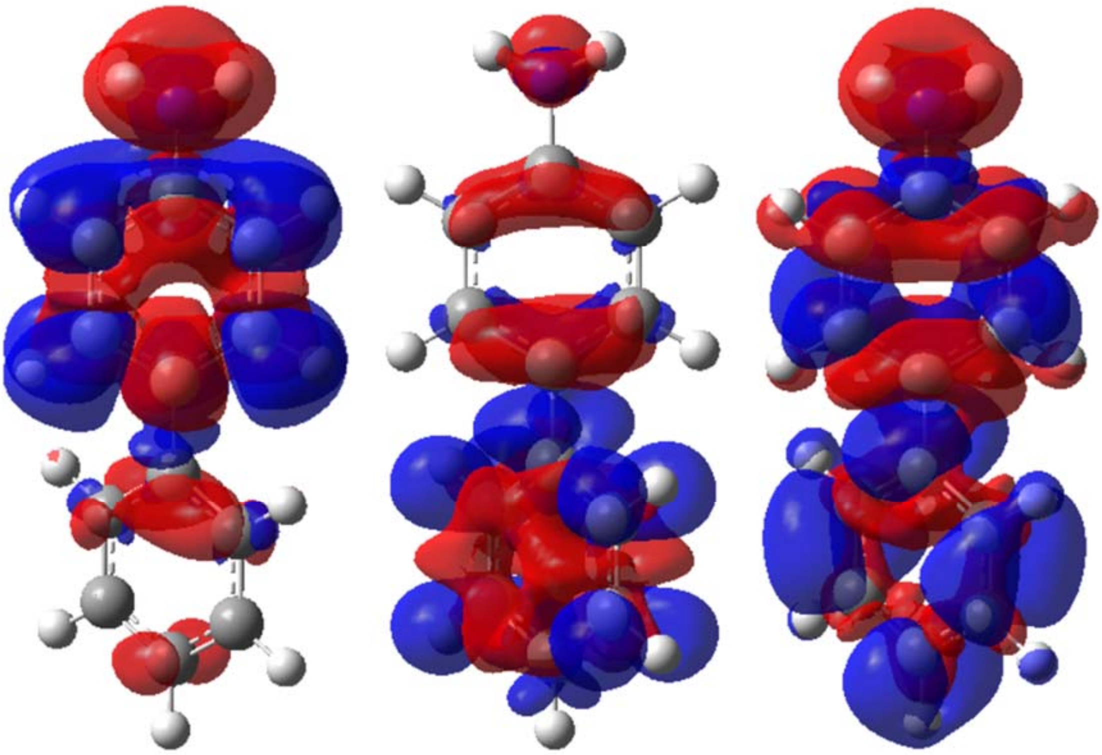

Figure S48. Projection of the electron density difference of the S_1 state (left), S_2 state (middle) and S_3 state (right) on the electron isodensity surface of the ground state, S_0 for **3**. Areas of increase (depletion) of electron density in the excited states are shown in blue (red). The calculations were performed with the ADC(2)/cc-pVDZ method.

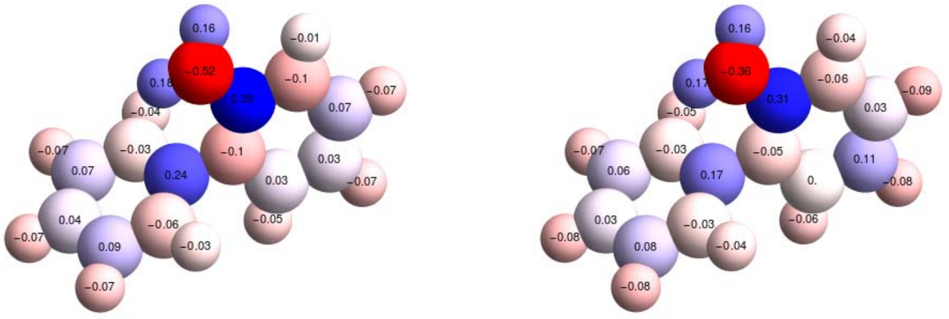

Figure S49. The computed charges (a.u.) at MP2/cc-pVDZ and ADC(2)/cc-pVDZ levels of theory for **1** in S_0 (left) and optimized S_1 (right), respectively.

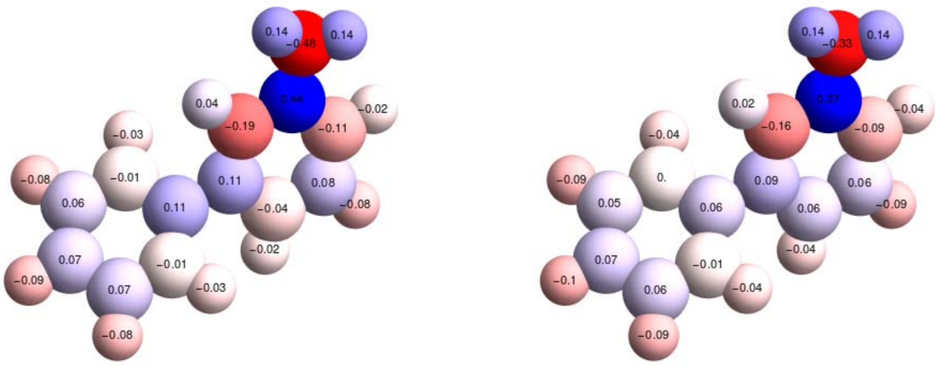

Figure S50. The computed charges (a.u.) at MP2/cc-pVDZ and ADC(2)/cc-pVDZ levels of theory for **2** in S_0 (left) and optimized S_1 (right), respectively.

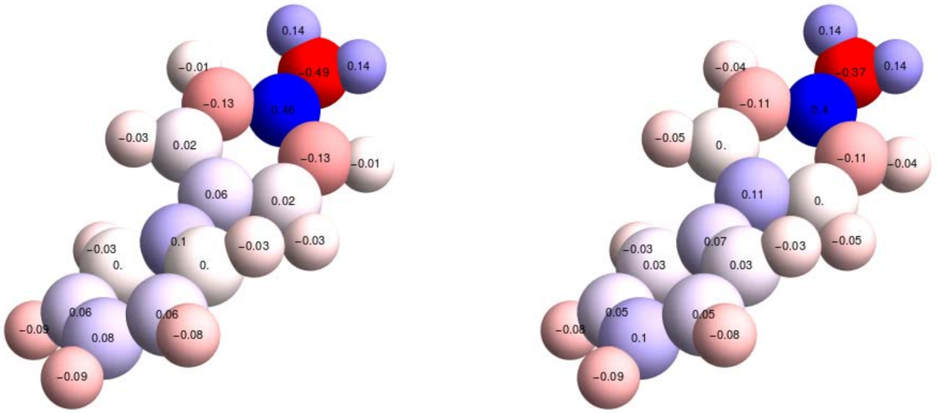

Figure S51. The computed charges (a.u.) at MP2/cc-pVDZ and ADC(2)/cc-pVDZ levels of theory for **3** in S_0 (left) and optimized S_1 (right), respectively.

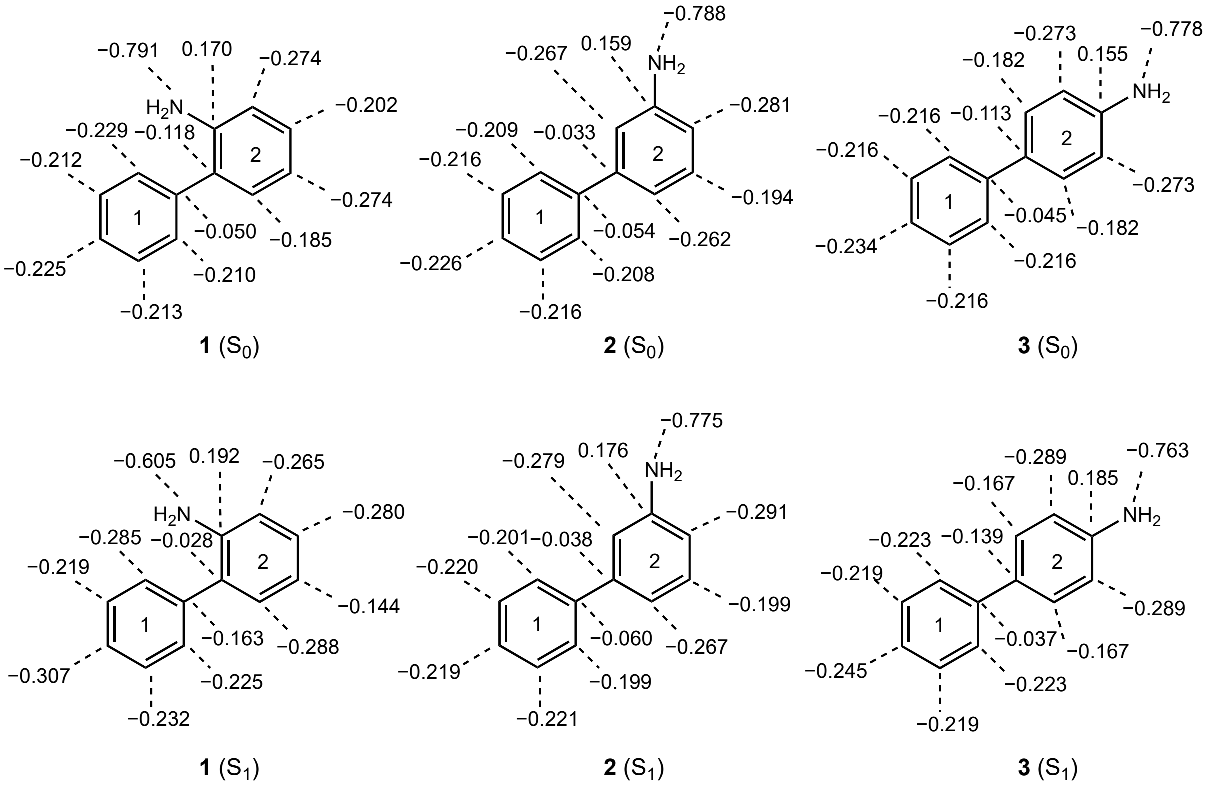

Figure S52. Computed NPA (CPCM(CH_3CN)-PBE0-D3/Def2-TZVPP) charges (a.u.) for **1-3** in the S_0 (top) and S_1 (bottom) states.

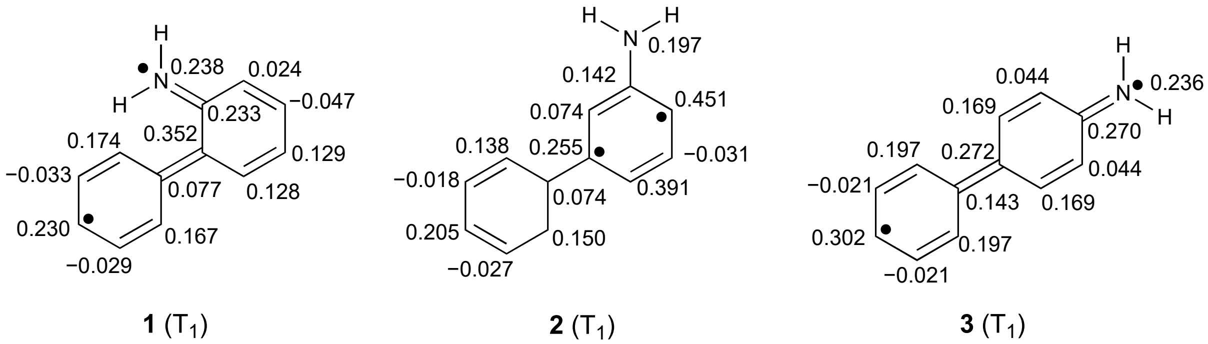

Figure S53. Computed triplet spin densities (CPCM(CH_3CN)-PBE0-D3/Def2-TZVPP) for **1-3** based on Hirshfeld population analyses.

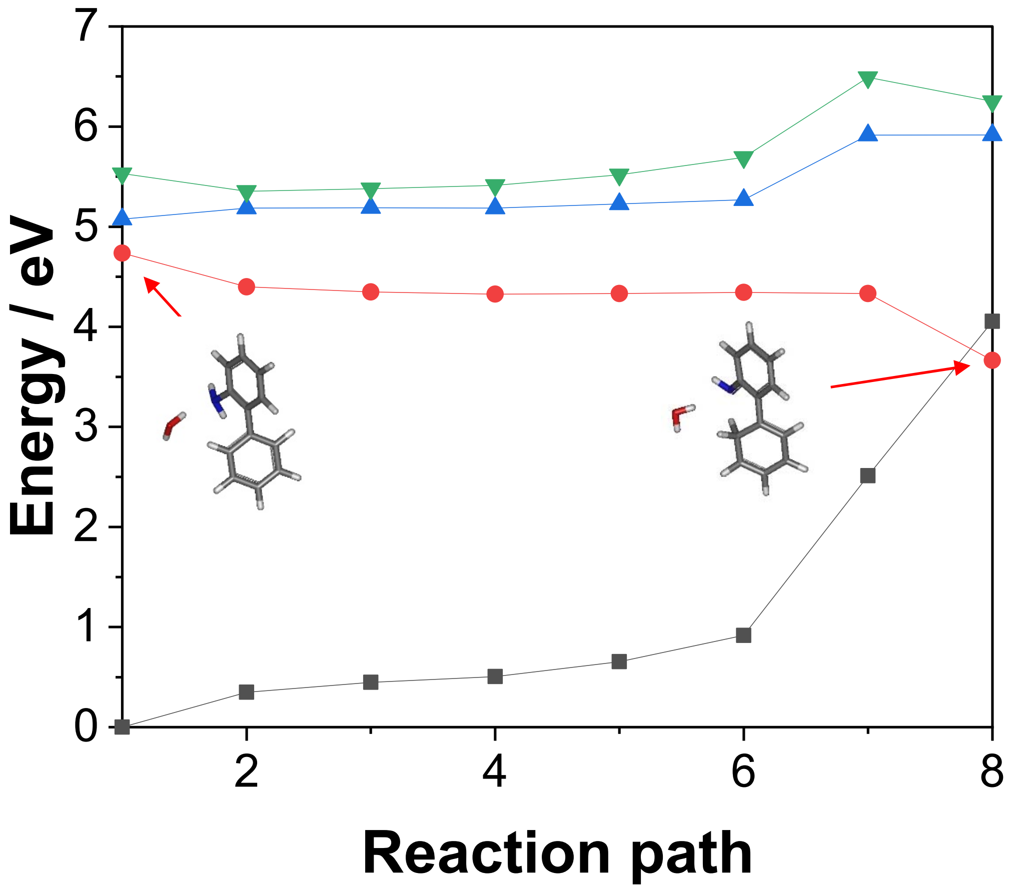

Figure S54. Potential energy profiles of the three lowest singlet excited states (S_1 red, S_2 blue, S_3 green) and the ground state (S_0 grey) along the reaction path from the FC geometry of **1·H_2O** to the nearest S1/S0 conical intersection. For the Cartesian coordinates of the Franck-Condon geometry and the minimum energy S_1/S_0 conical intersection see Tables S17 and S18.

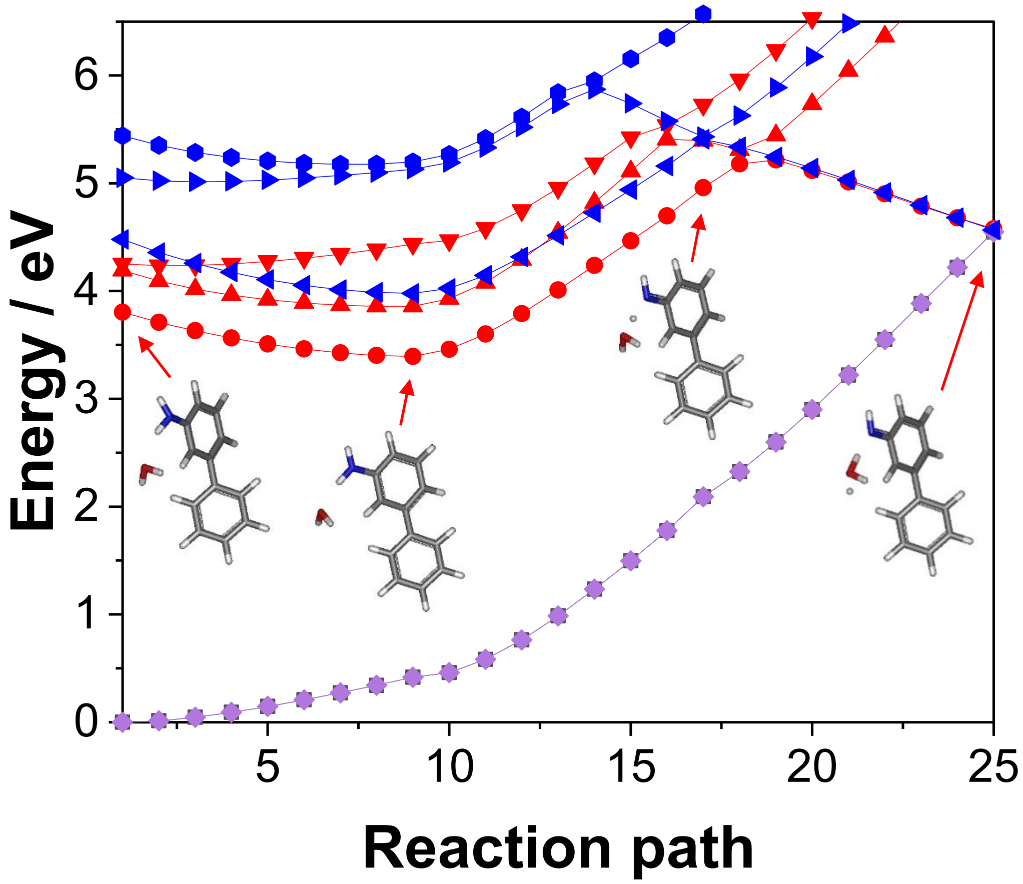

Figure S55. Potential energy profiles of the ground state (S_0 magenta), the three lowest singlet excited states (S_1-S_3 blue) and the three lowest triplet states (T_1-T_3 red) along the reaction path from the FC geometry of **2** to the minimum energy T_1/S_0 conical intersection (step 25). For Cartesian coordinates of the relevant structures (**A**-**D** in Figure 8) see Tables S19-S22.

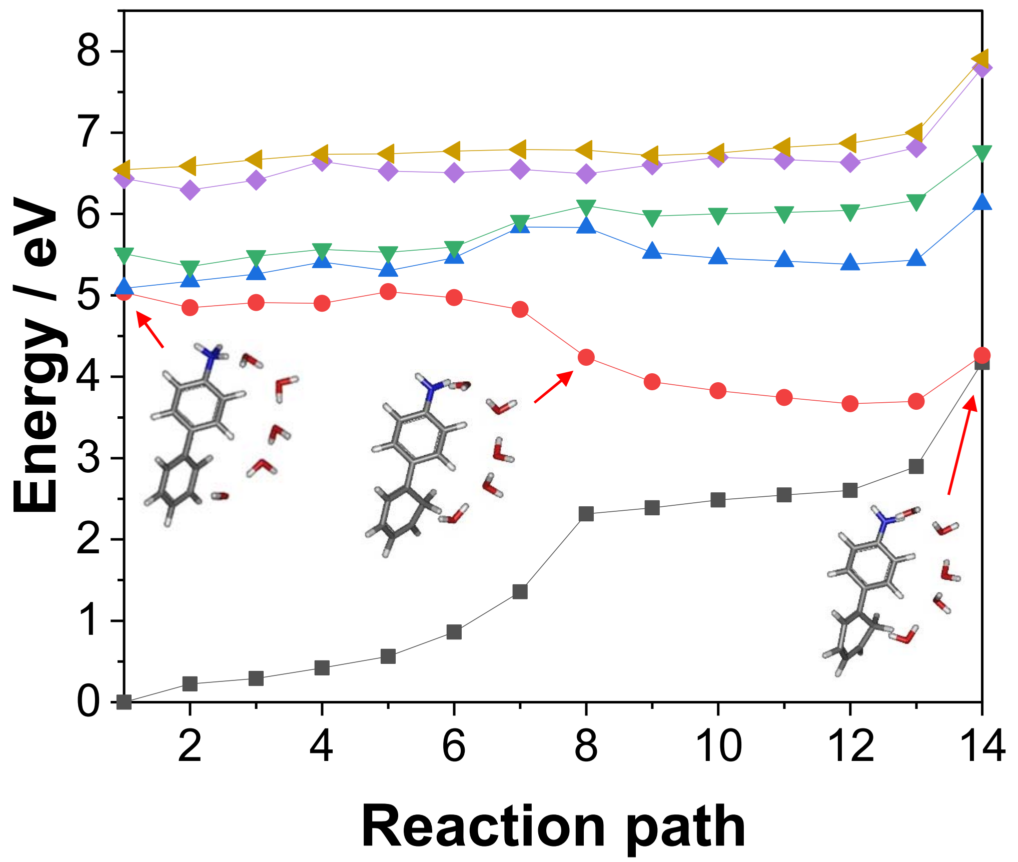

Figure S56. Potential energy profiles of the ground state (S_0 grey) and the five lowest singlet excited states (S_1-S_5 red, blue, green, magenta, orange) along the reaction path from the FC geometry of **3** to the minimum energy S_1/S_0 conical intersection. For Cartesian coordinates of the relevant structures (**A-D** in Figure 9) see Tables S23-S26.

Table S13

MP2/cc-pvdz minimum of **1** optimized in the gas phase (**A** in Figure 7)

C	-1.6036105	0.7016508	0.0305986
C	-0.1833455	0.7014278	0.0517732
C	0.5027399	1.9329360	0.0899605
C	-0.1837018	3.1544201	0.1223345
C	-1.5876915	3.1498609	0.1113351
C	-2.2848883	1.9355548	0.0664564
C	0.5884845	-0.5674846	0.0115489
C	1.6229538	-0.7396179	-0.9339337
C	2.3862144	-1.9169553	-0.9555581
C	2.1254259	-2.9436064	-0.0330380
C	1.0968486	-2.7846499	0.9105052
C	0.3336731	-1.6070901	0.9340223
N	-2.3314881	-0.5042714	0.0415306
H	1.8156722	0.0547800	-1.6622439
H	3.1824380	-2.0350139	-1.6969469
H	2.7197228	-3.8620212	-0.0494402
H	0.8924809	-3.5774766	1.6367221
H	-0.4604198	-1.4803193	1.6757098
H	1.5972560	1.9161420	0.1164695
H	0.3697727	4.0966095	0.1614093
H	-2.1449974	4.0911153	0.1375092
H	-3.3806033	1.9356350	0.0529808
H	-3.2457474	-0.3936693	-0.3943504
H	-1.8271890	-1.2579564	-0.4253549

Table S14

Geometry of the lowest energy point of **1** on the S_1 state MEP optimized with ADC(2)/cc-pVDZ (**B** in Figure 7)

C	-1.5416848	0.7715686	0.1060358
C	-0.0767627	0.7224640	0.0461459
C	0.5579822	1.9988650	-0.0990473
C	-0.1906904	3.1969421	-0.0191634
C	-1.5990564	3.2201104	0.1403228
C	-2.2796388	1.9753775	0.1849047
C	0.6137614	-0.5352512	0.0334333
C	1.7843829	-0.7204051	-0.7817890
C	2.4232269	-1.9529536	-0.8570342
C	1.9650853	-3.0647967	-0.0996295
C	0.8599865	-2.8935384	0.7506831
C	0.1873815	-1.6643848	0.8307568
N	-2.1954249	-0.4050957	-0.0819201
H	2.1259932	0.1106066	-1.4089975
H	3.2851123	-2.0688416	-1.5235337
H	2.4835698	-4.0264562	-0.1544862
H	0.5364154	-3.7192456	1.3951131
H	-0.5630931	-1.5168255	1.6165773
H	1.6487106	2.0445348	-0.1592053
H	0.3491448	4.1484835	-0.0731927
H	-2.1467958	4.1633714	0.1908747
H	-3.3748223	1.9286279	0.1885471
H	-3.2106528	-0.4471088	-0.0861675
H	-1.6421349	-1.2638196	-0.1805912

Table S15

Geometry of the highest energy point of **1** after **B** on the S_1 state MEP optimized with ADC(2)/cc-pVDZ (**C** in Figure 7)

C	-1.4911359	0.7763464	0.0859706
C	-0.0227189	0.7652697	0.0404225
C	0.5893375	2.0476471	-0.0787749
C	-0.1819493	3.2257406	-0.0273120
C	-1.5954517	3.2152614	0.1055078
C	-2.2496133	1.9675414	0.1568142
C	0.6624900	-0.4922054	0.0703064
C	1.8187928	-0.7278970	-0.7481972
C	2.3622287	-1.9978636	-0.8756705
C	1.8007683	-3.1101957	-0.1692975
C	0.7287280	-2.8958345	0.6974764
C	0.1351968	-1.6192575	0.8354805
N	-2.0854183	-0.4243955	-0.1310277
H	2.2288484	0.1033229	-1.3340775
H	3.2191272	-2.1532772	-1.5401037
H	2.2400410	-4.1069317	-0.2707588
H	0.3484522	-3.7250427	1.3065394
H	-0.4693454	-1.4139799	1.7299431
H	1.6801095	2.1131026	-0.1225544
H	0.3356614	4.1898519	-0.0709709
H	-2.1623733	4.1477408	0.1479324
H	-3.3436998	1.9032987	0.1767891
H	-3.0936482	-0.5217739	-0.0286297

Table S16

Post conical intersection structure of **1** on the S_1 state MEP optimized with ADC(2)/cc-pVDZ (**D** in Figure 7)

C	-1.5118923	0.7760514	0.0280954
C	-0.0744362	0.7986227	0.0039871
C	0.5654981	2.0407386	-0.0347949
C	-0.1865890	3.2403456	-0.0132939
C	-1.5767398	3.2221364	0.1054137
C	-2.2359720	1.9776298	0.1427767
C	0.6621434	-0.4878521	0.0304300
C	1.7675394	-0.7140219	-0.7655828
C	2.3580053	-2.0027666	-0.8791244
C	1.7899624	-3.0848025	-0.1514527
C	0.6894591	-2.9045310	0.6687259
C	-0.0283367	-1.6155144	0.7339966
N	-2.1525330	-0.4176002	-0.2655251
H	2.1957571	0.1146554	-1.3438434
H	3.2249196	-2.1531011	-1.5307227
H	2.2566957	-4.0754753	-0.2182129
H	0.3314676	-3.7387000	1.2836869
H	-0.3750882	-1.3697216	1.7602949
H	1.6590707	2.0873454	-0.0827482
H	0.3424628	4.1961887	-0.0636748
H	-2.1510257	4.1513082	0.1545783
H	-3.3308049	1.9277784	0.1871713
H	-3.1449282	-0.4156766	0.0099456
H	-1.0746417	-1.5494925	0.1740884

Table S17

MP2/cc-pvdz minimum of the **1** monohydrate optimized in the gas phase (**1·H_2O** S_{0min})

C	-1.3364513	0.8808715	-0.1836421
C	0.0817463	0.8814551	-0.2164351
C	0.7600580	2.1198697	-0.2020005
C	0.0641842	3.3360773	-0.1380212
C	-1.3400969	3.3243845	-0.0914037
C	-2.0306896	2.1042293	-0.1106083
C	0.8608802	-0.3842486	-0.2938682
C	0.6791833	-1.4161194	0.6569174
C	1.4588775	-2.5834335	0.5879097
C	2.4254773	-2.7386778	-0.4210250
C	2.6096385	-1.7178739	-1.3700647
C	1.8332649	-0.5493603	-1.3061180
N	-2.0681774	-0.3401981	-0.1558680
O	-2.1117545	-1.2939552	2.6378241
H	0.6119331	4.2834242	-0.1173414
H	1.8557575	2.1122264	-0.2188243
H	-3.1266085	2.0939508	-0.0742848
H	-1.9009079	4.2629690	-0.0340726
H	1.9672768	0.2427890	-2.0514982
H	3.3577059	-1.8324164	-2.1619102
H	3.0318404	-3.6494482	-0.4676885
H	1.3177650	-3.3710406	1.3364574
H	-0.0626158	-1.3000678	1.4547852
H	-2.9675080	-0.2283660	-0.6282438
H	-1.5495570	-1.0822374	-0.6330411
H	-2.2268472	-0.9157519	1.7449938
H	-2.1943746	-2.2390517	2.4570722

Table S18

Geometry of the S_1/S_0 conical intersection of **1·H_2O** optimized with ADC(2)/cc-pVDZ in the gas phase (**1·H_2O** S_1/S_0 CI)

C	-1.2812346	0.9158404	-0.2226219
C	0.1531549	0.9759360	-0.2626528
C	0.7632107	2.2306952	-0.2906654
C	-0.0152407	3.4158876	-0.2729532
C	-1.4022222	3.3635575	-0.1528034
C	-2.0303678	2.1019185	-0.1048493
C	0.9317105	-0.2912174	-0.2777455
C	0.3140910	-1.4488731	0.4410860
C	1.0589458	-2.7161692	0.3097520
C	2.1395709	-2.8422956	-0.5484664
C	2.6529300	-1.7283259	-1.2612503
C	2.0162872	-0.4618229	-1.1080732
N	-1.9013792	-0.3009674	-0.4841883
O	-2.3983688	-1.3731602	2.6473813
H	0.4915590	4.3834336	-0.3332459
H	1.8551290	2.3005280	-0.3404549
H	-3.1227097	2.0283032	-0.0461127
H	-1.9995753	4.2781851	-0.1069125
H	2.3973602	0.3946343	-1.6782683
H	3.5103817	-1.8334624	-1.9336114
H	2.6313141	-3.8167743	-0.6537287
H	0.7421321	-3.5798061	0.9067537
H	0.0413675	-1.2287078	1.4947291
H	-2.8935726	-0.2967122	-0.1992078
H	-0.7597009	-1.4209653	-0.0546035
H	-1.9311385	-0.8232579	2.0087500
H	-1.9636410	-2.2264002	2.5239657

Table S19

MP2/cc-pvdz minimum of **2** monohydrate optimized in the gas phase (**A** in Figure 8)

C	0.0000000	0.0000000	0.0000000
C	0.0000000	0.0000000	1.4116780
C	1.2414350	0.0000000	2.0844042
C	2.4478927	0.0050037	1.3680968
C	2.4346848	0.0061223	-0.0365157
C	1.2058819	0.0017890	-0.7173111
C	-1.2737811	-0.0005780	2.1719987
C	-2.3412871	-0.8404121	1.7927196
C	-3.5282262	-0.8299568	2.5409480
C	-3.6684737	0.0105500	3.6538682
C	-2.6118624	0.8599996	4.0487977
C	-1.4228008	0.8406187	3.2905155
N	-2.7669522	1.7699916	5.1013863
O	-0.0074381	2.9934948	5.6597329
H	-4.3552831	-1.4890768	2.2589928
H	-2.2256543	-1.5193767	0.9429857
H	-4.6037668	0.0157367	4.2239792
H	1.2538264	-0.0376164	3.1786037
H	3.4007809	-0.0052055	1.9067624
H	3.3746817	0.0078941	-0.5961931
H	1.1862309	0.0092085	-1.8115716
H	-0.9553404	0.0228809	-0.5337028
H	-0.6046360	1.5048768	3.5816512
H	-1.8763098	2.0686251	5.5012471
H	-3.3926908	1.4305268	5.8278559
H	0.7861828	2.4961332	5.4221246
H	-0.0759115	3.6314356	4.9364969

Table S20

Geometry of the lowest energy point of **2** monohydrate on the S_1 state MEP optimized with ADC(2)/cc-pVDZ (**B** in Figure 8)

C	0.0000000	0.0000000	0.0000000
C	0.0000000	0.0000000	1.4345240
C	1.2868912	0.0000000	2.0713839
C	2.4702285	-0.0060308	1.3275610
C	2.4446137	-0.0001952	-0.0809440
C	1.1894072	-0.0012040	-0.7294127
C	-1.2097762	0.0287635	2.2083673
C	-2.5229245	-0.2409598	1.6366873
C	-3.7134771	-0.2671967	2.3843838
C	-3.7111353	-0.0129502	3.7755255
C	-2.4441071	0.2882885	4.3610282
C	-1.2276540	0.3210233	3.6199373
N	-2.3797837	0.5764357	5.6889044
O	0.2880034	1.2708910	6.5615127
H	-4.6517269	-0.5019095	1.8726977
H	-2.5881738	-0.4649261	0.5684825
H	-4.6164686	-0.0602445	4.3864652
H	1.3484984	-0.0608374	3.1633436
H	3.4318577	-0.0236797	1.8536547
H	3.3736874	-0.0004132	-0.6587288
H	1.1420815	0.0082911	-1.8241721
H	-0.9481712	0.0320836	-0.5463061
H	-0.3214604	0.6312682	4.1413900
H	-1.4805848	0.8220389	6.1226305
H	-3.2293373	0.5920855	6.2449538
H	0.9124831	0.5572398	6.3679704
H	0.6277965	1.9967948	6.0187071

Table S21

Geometry of the S_1/T_1 crossing of **2** monohydrate on the S_1 state MEP optimized with ADC(2)/cc-pVDZ (**C** in Figure 8)

C	0.0000000	0.0000000	0.0000000
C	0.0000000	0.0000000	1.4163540
C	1.2463640	0.0000000	2.0920573
C	2.4488272	-0.0184562	1.3715157
C	2.4357023	-0.0239422	-0.0332452
C	1.2055819	-0.0067359	-0.7152962
C	-1.2536980	0.0476938	2.1900988
C	-2.4285360	-0.6490349	1.7889800
C	-3.6057274	-0.6128210	2.5563864
C	-3.6470852	0.1225322	3.7491237
C	-2.4933582	0.8372220	4.1744328
C	-1.3121068	0.7919925	3.3835358
N	-2.4797343	1.5834490	5.3124076
O	-0.3094420	2.8090814	5.9618412
H	-4.4882039	-1.1755822	2.2371093
H	-2.4001615	-1.2602936	0.8815539
H	-4.5513164	0.1486497	4.3675406
H	1.2662305	0.0087446	3.1878413
H	3.4004824	-0.0328819	1.9113512
H	3.3757421	-0.0307353	-0.5918045
H	1.1877348	0.0259751	-1.8090997
H	-0.9510995	0.0469845	-0.5420858
H	-0.4351818	1.3642502	3.7110846
H	-1.2389878	2.2987198	5.7285836
H	-3.3815893	1.5549445	5.8025161
H	0.5080808	2.0418762	5.5747885
H	-0.2356936	3.4840598	5.2406553

Table S22

Geometry of the T_1/S_0 crossing of **2** monohydrate on the T_1 state MEP optimized with ADC(2)/cc-pVDZ (**D** in Figure 8)

C	0.0000000	0.0000000	0.0000000
C	0.0000000	0.0000000	1.4120130
C	1.2380175	0.0000000	2.0923938
C	2.4438300	-0.0019397	1.3766634
C	2.4333438	-0.0036317	-0.0270024
C	1.2084580	0.0016451	-0.7132011
C	-1.2675869	0.0215283	2.1822231
C	-2.3872864	-0.7581462	1.8039265
C	-3.5728369	-0.7305513	2.5615575
C	-3.6634199	0.0719047	3.7012987
C	-2.5545143	0.8667240	4.1152268
C	-1.3721952	0.8299506	3.3232522
N	-2.5686179	1.6559969	5.2202965
O	-0.3539528	2.9993478	5.8189580
H	-4.4239495	-1.3537671	2.2695340
H	-2.3200511	-1.4171365	0.9323278
H	-4.5848608	0.0879542	4.2952257
H	1.2497233	0.0039812	3.1878681
H	3.3916664	-0.0031695	1.9205293
H	3.3747789	-0.0009993	-0.5820223
H	1.1932106	0.0279624	-1.8059940
H	-0.9525456	0.0347618	-0.5410414
H	-0.5267156	1.4534883	3.6279164
H	-1.2177960	2.4725921	5.6469675
H	-3.4794915	1.6065104	5.6925147
H	0.6028736	2.0954817	5.3144798
H	-0.3249579	3.6154935	5.0651704

Table S23

MP2/cc-PVDZ minimum of **3** pentahydrate optimized in the gas phase (**A** in Figure 9)

C	-2.3694646	-1.0637998	-0.1441668
C	-0.9831083	-1.0723468	-0.4122657
C	-0.3136805	0.1543380	-0.6237402
C	-1.0096926	1.3713664	-0.5675218
C	-2.3858443	1.3432432	-0.3022354
C	-3.0802611	0.1457815	-0.0904900
C	-0.2287764	-2.3497420	-0.4603695
C	0.6571330	-2.6130016	-1.5291781
C	1.3768928	-3.8201209	-1.5741489
C	1.2240822	-4.7735332	-0.5489403
C	0.3419277	-4.5177807	0.5170087
C	-0.3839592	-3.3150763	0.5590929
N	-3.1003559	2.6284170	-0.2357952
O	2.9061794	0.6132115	-0.1345923
O	2.1144483	2.7917561	1.2903415
O	0.4664629	4.3206157	-0.0966860
O	2.8861241	-1.9295799	0.8595818
O	-1.8003358	4.3302056	1.1939470
H	-0.4813608	2.3213167	-0.7036465
H	0.7694497	0.1685664	-0.7826482
H	-1.0592837	-3.1123527	1.3980961
H	0.2180192	-5.2572115	1.3149699
H	1.7832974	-5.7137888	-0.5859580
H	2.0479174	-4.0231610	-2.4152822
H	0.7631666	-1.8799498	-2.3365201
H	-2.9017123	-2.0095291	0.0005508
H	-4.1586065	0.1442790	0.1062256
H	-4.0561751	2.5066394	0.1192275
H	-2.5629012	3.3445514	0.4218236
H	-3.1802311	3.0592843	-1.1667989
H	0.9346119	5.0742637	-0.4801852
H	1.1626287	3.8139677	0.4031862
H	-0.9064455	4.4560951	0.7618279
H	-1.5951471	4.1358268	2.1200140
H	2.8811389	3.1070957	1.7860263
H	2.4546438	2.0040222	0.8062132
H	2.9186008	-0.2768758	0.2881671
H	3.7076149	0.6134894	-0.6754618
H	2.4460562	-2.1620862	1.6885586
H	2.4969461	-2.5583966	0.2317725

Table S24

Post S_2/S_1 conical intersection geometry of **3** pentahydrate on the S_1 state MEP optimized with ADC(2)/cc-pVDZ (**B** in Figure 9)

C	-2.3323819	-1.1547437	-0.1131700
C	-0.9437866	-1.1900755	-0.4288468
C	-0.2908310	0.0419928	-0.7254215
C	-0.9842588	1.2518765	-0.6536001
C	-2.3643279	1.2696980	-0.3551358
C	-3.0342086	0.0544301	-0.0958654
C	-0.1957068	-2.4307783	-0.4548838
C	0.8565906	-2.6424513	-1.4459045
C	1.4181972	-3.9726082	-1.5339086
C	1.2298523	-4.9164454	-0.4803680
C	0.3708190	-4.6203041	0.6265611
C	-0.4159272	-3.4222437	0.5709062
N	-3.0420977	2.5278131	-0.2708663
O	2.8713058	0.8903408	-0.0422146
O	1.9506366	2.9188124	1.2217406
O	0.3531737	4.2934737	0.0708974
O	2.8203634	-1.6768485	0.6319548
O	-1.7819879	4.4059975	1.2914511
H	-0.4580790	2.1950831	-0.8322900
H	0.7862716	0.0466813	-0.9145421
H	-1.1367621	-3.2012391	1.3663956
H	0.2509685	-5.3269634	1.4517140
H	1.7389548	-5.8845110	-0.5313073
H	2.0082354	-4.2545758	-2.4124848
H	0.9002409	-1.9730261	-2.3136615
H	-2.8704207	-2.0962702	0.0413271
H	-4.1149308	0.0563122	0.0889777
H	-4.0538555	2.3929172	-0.1905593
H	-2.3447633	3.6901589	0.8791686
H	-2.8905556	3.0685635	-1.1296972
H	0.6475051	5.1819218	-0.1788084
H	1.1603299	3.6933190	0.6016425
H	-0.5279145	4.3737141	0.6548262
H	-1.8272629	4.2663777	2.2484182
H	2.6981125	3.3283799	1.6797632
H	2.3337402	2.1241035	0.7169213
H	2.8458319	-0.0384549	0.3176055
H	3.6491745	0.8948996	-0.6168335
H	2.3471171	-2.0591459	1.3856250
H	2.3726357	-2.1061922	-0.1255126

Table S25

Characteristic ESPT to Ring-1 geometry of **3** pentahydrate on the S_1 state MEP optimized with ADC(2)/cc-pVDZ (**C** in Figure 9)

C	-2.3388279	-1.1869566	-0.1549691
C	-0.9324386	-1.1818968	-0.4190762
C	-0.2886406	0.0565366	-0.7283958
C	-0.9959862	1.2506242	-0.6942210
C	-2.3935368	1.2375094	-0.4096666
C	-3.0499704	0.0042685	-0.1162620
C	-0.1386326	-2.4154684	-0.3584838
C	1.0766914	-2.5312971	-1.2526784
C	1.4554826	-3.9310099	-1.4698253
C	1.2233003	-4.8896026	-0.4697607
C	0.4031510	-4.5948010	0.6405992
C	-0.3725773	-3.3765458	0.6039994
N	-3.0962154	2.4141934	-0.3868611
O	2.7438704	0.9065348	-0.1072811
O	1.9874485	2.8586754	1.3518674
O	0.3082353	4.3039275	-0.1005226
O	2.8820683	-1.5228329	0.5739078
O	-1.8911620	4.4692990	1.4195176
H	-0.4861429	2.2076326	-0.8587856
H	0.7866862	0.0754131	-0.9251508
H	-1.1597617	-3.2223983	1.3535014
H	0.2444089	-5.3213430	1.4444096
H	1.6576538	-5.8896694	-0.5754947
H	2.0029487	-4.2150021	-2.3775769
H	0.9989158	-1.9422452	-2.1881824
H	-2.8528837	-2.1375691	0.0203377
H	-4.1279911	0.0019441	0.0829244
H	-4.1138638	2.3543462	-0.3542142
H	-2.4253172	3.7056607	1.1504616
H	-2.7465362	3.1732041	-0.9741263
H	0.5471897	5.1930047	-0.3957694
H	1.4459497	3.4552173	0.7808228
H	-0.4322381	4.4526753	0.5322784
H	-1.8855173	4.4194429	2.3851986
H	2.6909607	3.4208851	1.6980648
H	2.5276539	1.6783647	0.4916493
H	2.7901088	-0.5261701	0.4658813
H	3.6169150	1.0800250	-0.4820792
H	2.3831341	-1.7799420	1.3615479
H	1.9554653	-2.0546443	-0.5575709

Table S26

S_1/S_0 conical intersection geometry of **3** pentahydrate optimized with ADC(2)/cc-pVDZ (**D** in Figure 9)

C	-2.2983337	-1.2947310	-0.4617157
C	-0.8715257	-1.2947310	-0.4617157
C	-0.1531030	-0.0726469	-0.4617157
C	-0.8213642	1.1362226	-0.5401775
C	-2.2496259	1.1421051	-0.4770314
C	-2.9813928	-0.0859599	-0.3648002
C	-0.0669139	-2.5579514	-0.4290424
C	1.0635139	-2.7276313	-1.4373800
C	1.4236873	-4.1792081	-1.4471120
C	1.3202581	-5.0169802	-0.3693078
C	0.7054773	-4.5831039	0.8397772
C	-0.1800511	-3.3634145	0.6541574
N	-2.8677465	2.3163404	-0.5773111
O	2.5212655	1.2936983	-0.4401712
O	1.8554376	3.1311019	1.5507165
O	-0.1184779	4.3961815	-0.2196368
O	3.0769321	-1.2901502	0.9903861
O	-2.1239665	4.5673420	1.7197182
H	-0.2717254	2.0698192	-0.6631083
H	0.9325226	-0.0876891	-0.4832869
H	-1.0294507	-3.2222636	1.3455950
H	0.3954675	-5.3060080	1.6176568
H	1.6695920	-6.0552184	-0.4574432
H	1.6541982	-4.6581400	-2.4384225
H	0.7467722	-2.4136552	-2.4383816
H	-2.8519233	-2.2364305	-0.4210541
H	-4.0602751	-0.0607962	-0.1964803
H	-3.8889292	2.3746593	-0.6119862
H	-2.5072125	3.7114130	1.9862537
H	-2.3214456	3.1843718	-0.6570322
H	0.1807636	5.2441321	-0.5816069
H	1.3618125	3.6778686	0.9132132
H	-0.6932758	4.6512794	0.5300683
H	-2.0061792	5.0072909	2.5835742
H	2.5315425	3.7325537	1.8908701
H	2.4483562	1.8518712	0.3668022
H	2.8502891	-0.4666538	0.5142519
H	3.4669436	1.3201782	-0.6403138
H	2.2251048	-1.7766729	0.9594689
H	1.9329735	-2.0584216	-1.1862423

Table S27. Energies and lowest frequencies for structures computed with density functional theory (CPCM(CH_3CN)-PBE0-D3/def2-TZVPP) and time-dependent density functional theory (TD-CPCM(CH_3CN)-PBE0-D3/def2-TZVPP).

Structure	Electronic State	E (Hartree)	Lowest frequency (cm^{-1})	Oscillator Strength
1 (S_0)	S_0	−518.249820973	54.7180	N/A
2 (S_0)	S_0	−518.252473842	58.8191	N/A
3 (S_0)	S_0	−518.252775589	70.2348	N/A
1 (S_1)*	S_1	−518.115935698	69.2880	0.3913
2 (S_1)*	S_1	−518.116042331	32.0661	0.2338
3 (S_1)*	S_1	−518.114080020	15.8123	1.1427
1 (T_1)	T_1	−518.144970898	56.3583	N/A
2 (T_1)	T_1	−518.148449963	44.8309	N/A
3 (T_1)	T_1	−518.150733522	40.4575	N/A

*Computed at TD-CPCM(CH_3CN)-PBE0-D3/def2-TZVPP.

Table S28

1 (S_0) optimized at CPCM(CH_3CN)-PBE0-D3/def2-TZVPP

C	2.93666100	-1.00483600	-0.76262300
C	1.54940300	-1.03062500	-0.76393900
C	0.81666800	-0.15663000	0.03961200
C	1.51443300	0.74201300	0.84990900
C	2.90132100	0.77101600	0.85007900
C	3.61784200	-0.10156400	0.04185200
H	3.48716700	-1.69060200	-1.39588100
H	1.02109300	-1.73223400	-1.39926600
H	0.96357200	1.40951200	1.50392000
H	3.42348300	1.47151300	1.49105000
H	4.70095100	-0.07949700	0.04157100
C	-0.66276100	-0.21334100	0.05379000
C	-1.44871100	0.91581000	-0.25210100
C	-1.29854900	-1.41336800	0.36454000
C	-2.84251700	0.79845100	-0.19724300
C	-2.67951600	-1.52354600	0.40889500
H	-0.68100300	-2.27492800	0.59409000
C	-3.44865600	-0.40059800	0.13098700
H	-3.44964900	1.66636000	-0.43173100
H	-3.14639100	-2.46657100	0.66383900
H	-4.53052400	-0.45690600	0.16661000
N	-0.88070500	2.09792100	-0.68454200
H	-1.44404900	2.92373000	-0.57069100
H	0.08659100	2.24748300	-0.45427700

Table S29

2 (S_0) optimized at CPCM(CH_3CN)-PBE0-D3/def2-TZVPP

C	3.01744100	-1.30444200	-0.47247900
C	1.64553100	-1.10633500	-0.50950600
C	1.07737700	0.07362700	-0.02437700
C	1.92838400	1.04964300	0.49877700
C	3.30034000	0.85224500	0.53729200
C	3.85161200	-0.32604300	0.05169500
H	3.43713700	-2.22454500	-0.86191200
H	1.00680000	-1.86864800	-0.93998300
H	1.50802900	1.96495600	0.89852100
H	3.94101000	1.61974600	0.95546700
H	4.92350400	-0.48059100	0.08138600
C	-0.38455600	0.28627100	-0.06398800
C	-0.91367900	1.54866900	-0.34150800
C	-1.25381600	-0.77496700	0.17602900
C	-2.28918300	1.72408700	-0.37433100
C	-2.63974700	-0.60422600	0.14382400
H	-0.85184700	-1.75332200	0.41649400
C	-3.15130900	0.66722100	-0.13572600
H	-2.69965400	2.70268000	-0.59571100
H	-0.25390800	2.38081800	-0.55241100
H	-4.22470700	0.81902500	-0.16412500
N	-3.48650700	-1.68104200	0.33605900
H	-3.09548000	-2.45554700	0.84703700
H	-4.41570000	-1.45176800	0.64862200

Table S30

3 (S_0) optimized at CPCM(CH_3CN)-PBE0-D3/def2-TZVPP

C	-2.39136900	-1.14699500	0.34830800
C	-1.00896000	-1.14023400	0.34605900
C	-0.28002700	-0.00041400	-0.00189800
C	-1.00919300	1.13930900	-0.34967700
C	-2.39154200	1.14466700	-0.35530100
C	-3.11292600	-0.00190400	-0.00569800
C	1.19401600	0.00020000	-0.00001200
C	1.91472400	1.14261600	0.36050600
C	3.30121700	1.14439100	0.35940000
C	4.00377300	0.00087200	0.00283200
C	3.30252200	-1.14300700	-0.35506100
C	1.91601500	-1.14188500	-0.35896500
H	-2.92514000	-2.04646900	0.63555700
H	-0.48410600	-2.03956100	0.64759800
H	1.38213900	2.03564900	0.66616000
H	3.83564600	2.04181300	0.64880200
H	5.08713100	0.00117700	0.00383300
H	3.83797000	-2.04015300	-0.64342800
H	1.38437800	-2.03509600	-0.66576500
H	-0.48458900	2.04108100	-0.64423000
H	-2.92571100	2.04390100	-0.64244300
N	-4.49207800	-0.01942800	-0.06404800
H	-4.94611500	-0.72774800	0.48863500
H	-4.94655800	0.87570800	0.01066000

Table S31

1 (S_1) optimized at TD-CPCM(CH_3CN)-PBE0-D3/def2-TZVPP

C	2.98260400	-1.19542000	-0.39769400
C	1.61400100	-1.28140200	-0.38686400
C	0.79859700	-0.18262200	0.03578700
C	1.49884100	0.96053600	0.56141900
C	2.87594400	1.02339000	0.53997200
C	3.64443700	-0.03240200	0.04390800
H	3.56238000	-2.03761400	-0.75995400
H	1.13849800	-2.17863800	-0.76513100
H	0.94851900	1.74250000	1.07050400
H	3.36763200	1.89677500	0.95570800
H	4.72573100	0.02261100	0.04195800
C	-0.62227100	-0.26978500	-0.00242800
C	-1.45829700	0.91547100	-0.17278300
C	-1.33603500	-1.48719400	0.10672600
C	-2.84898000	0.87674300	0.00234600
C	-2.71536100	-1.51080200	0.23741500
H	-0.78592600	-2.41581100	0.18018300
C	-3.48660600	-0.33556000	0.22633500
H	-3.41613500	1.79114400	-0.13189900
H	-3.21063400	-2.46548900	0.37276800
H	-4.56142000	-0.38135100	0.33825300
N	-0.87734600	2.03264900	-0.63093200
H	0.10749500	2.04588300	-0.84821700
H	-1.41594500	2.86572800	-0.80247200

Table S32

2 (S_1) optimized at TD-CPCM(CH_3CN)-PBE0-D3/def2-TZVPP

C	3.02865800	-1.40819400	0.00006000
C	1.66296800	-1.23846600	0.00009500
C	1.06633100	0.06168800	0.00002800
C	1.97888300	1.16162200	-0.00021800
C	3.34296900	0.97281500	-0.00028500
C	3.90151300	-0.31202200	-0.00007800
H	3.43233700	-2.41563400	0.00016300
H	1.03874400	-2.12287700	0.00011300
H	1.61047900	2.17923700	-0.00041700
H	3.99257300	1.84202000	-0.00052600
H	4.97468500	-0.45402100	-0.00008300
C	-0.35149800	0.23864700	0.00021700
C	-0.95136100	1.55173400	0.00044300
C	-1.26820200	-0.83094100	0.00035200
C	-2.32296800	1.76670500	0.00004600
C	-2.65587300	-0.60414000	-0.00008200
H	-0.93349700	-1.85950300	0.00068000
C	-3.21347600	0.70681300	-0.00037200
H	-2.69304600	2.78456100	0.00017500
H	-0.31216900	2.42367000	0.00115900
H	-4.28663100	0.84507400	-0.00046300
N	-3.49716400	-1.64551900	-0.00022000
H	-3.15693300	-2.59300900	0.00008400
H	-4.49405400	-1.50844400	-0.00057700

Table S33

3 (S_1) optimized at TD-CPCM(CH_3CN)-PBE0-D3/def2-TZVPP

C	-2.38915600	-1.21615500	-0.00246200
C	-1.02532600	-1.22028100	-0.00583300
C	-0.25317700	0.00000300	-0.00480600
C	-1.02533500	1.22028000	-0.00688200
C	-2.38916500	1.21615900	-0.00354000
C	-3.11829500	-0.00000400	0.00145800
C	1.15960700	0.00000500	-0.00139500
C	1.92672100	1.22283600	0.00095900
C	3.29759700	1.20946500	0.00339200
C	4.01699300	-0.00000400	0.00368800
C	3.29759400	-1.20946700	0.00225900
C	1.92671400	-1.22282700	-0.00009300
H	-2.93311400	-2.15440000	-0.00286100
H	-0.51959900	-2.17502500	-0.00891400
H	1.42220700	2.17980700	0.00181200
H	3.83498700	2.15185200	0.00534500
H	5.10001800	-0.00000700	0.00544000
H	3.83498300	-2.15186000	0.00322700
H	1.42220200	-2.17980600	-0.00043000
H	-0.51962500	2.17502700	-0.01112300
H	-2.93312900	2.15439200	-0.00488200
N	-4.46851300	-0.00000500	0.00481000
H	-4.98898300	-0.85913700	0.02951600
H	-4.98899100	0.85913600	0.02872000

Table S34

1 (T_1) optimized at CPCM(CH_3CN)-PBE0-D3/def2-TZVPP

C	3.00748500	-1.19507600	-0.23321700
C	1.64159100	-1.28498800	-0.33132300
C	0.79254400	-0.16804400	-0.03455700
C	1.45412400	1.01487700	0.43682900
C	2.82463100	1.08089700	0.53443800
C	3.62650800	-0.01096900	0.18980000
H	3.61474700	-2.05568400	-0.49054400
H	1.20441100	-2.21063200	-0.68078600
H	0.86434800	1.85567500	0.77606000
H	3.28422900	1.98857200	0.90931400
H	4.70447000	0.04905500	0.26927400
C	-0.60246900	-0.25885400	-0.17373500
C	-1.49105000	0.93306300	-0.19279500
C	-1.31837200	-1.52011200	-0.20607900
C	-2.80462600	0.84000500	0.25267500
C	-2.61662400	-1.57761200	0.18702100
H	-0.78932800	-2.43629400	-0.43002000
C	-3.36701100	-0.40140500	0.51269900
H	-3.41114800	1.73943900	0.28593400
H	-3.10556000	-2.54370600	0.25291000
H	-4.39473600	-0.48594300	0.83824300
N	-1.01461000	2.06719500	-0.74984700
H	-1.62778100	2.84529300	-0.92254500
H	-0.12176400	2.07317600	-1.20944700

Table S35

2 (T_1) optimized at CPCM(CH_3CN)-PBE0-D3/def2-TZVPP

C	3.02425700	-1.41176800	0.00009800
C	1.66078700	-1.24351900	0.00005100
C	1.05710200	0.05490800	-0.00004500
C	1.97095700	1.15591100	0.00000300
C	3.33618900	0.96871900	0.00005700
C	3.89298600	-0.31206700	0.00008700
H	3.43117600	-2.41742600	0.00017600
H	1.03637200	-2.12732500	0.00006700
H	1.60050900	2.17181400	0.00005900
H	3.98566200	1.83762500	0.00006700
H	4.96647300	-0.45247800	0.00012600
C	-0.34603400	0.23009300	-0.00015400
C	-0.94479000	1.58199300	-0.00033300
C	-1.27092400	-0.84545200	-0.00016800
C	-2.30296900	1.78793000	0.00004500
C	-2.63151400	-0.63441400	0.00006000
H	-0.92469700	-1.87077900	-0.00037300
C	-3.18848400	0.71758200	0.00040700
H	-2.68751100	2.80082100	0.00002400
H	-0.29912400	2.44808800	-0.00089200
H	-4.26155700	0.85572600	0.00124500
N	-3.52250900	-1.64074700	-0.00013500
H	-3.22514300	-2.60112000	-0.00038000
H	-4.50997400	-1.45922300	0.00017400

Table S36

3 (T_1) optimized at CPCM(CH_3CN)-PBE0-D3/def2-TZVPP

C	-2.38185200	-1.21966000	-0.00633000
C	-1.02659200	-1.23090800	-0.00906600
C	-0.24073600	-0.00000800	-0.00663500
C	-1.02658200	1.23089800	-0.00925100
C	-2.38184300	1.21965400	-0.00651800
C	-3.11514400	0.00000200	0.00175500
C	1.15308500	-0.00001000	-0.00254000
C	1.92539700	1.22302200	0.00027000
C	3.29349600	1.20995200	0.00506100
C	4.01196800	0.00001200	0.00744300
C	3.29351200	-1.20994000	0.00487400
C	1.92541500	-1.22303100	0.00006500
H	-2.92802700	-2.15732500	-0.00942300
H	-0.52327300	-2.18669000	-0.01168500
H	1.42042800	2.17901800	-0.00086900
H	3.83201500	2.15129000	0.00718700
H	5.09427700	0.00001700	0.01134700
H	3.83204600	-2.15127000	0.00687600
H	1.42046300	-2.17903500	-0.00146300
H	-0.52326100	2.18668000	-0.01228100
H	-2.92801700	2.15732100	-0.00974600
N	-4.47532400	0.00000600	-0.02551500
H	-4.97506900	-0.85180500	0.16200100
H	-4.97505600	0.85185100	0.16189000

7. NMR spectra

^{1}H NMR ($CDCl_3$, 300 MHz) of *N*,*N*-dimethyl-2-aminobiphenyl

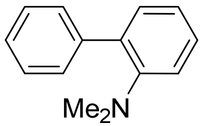

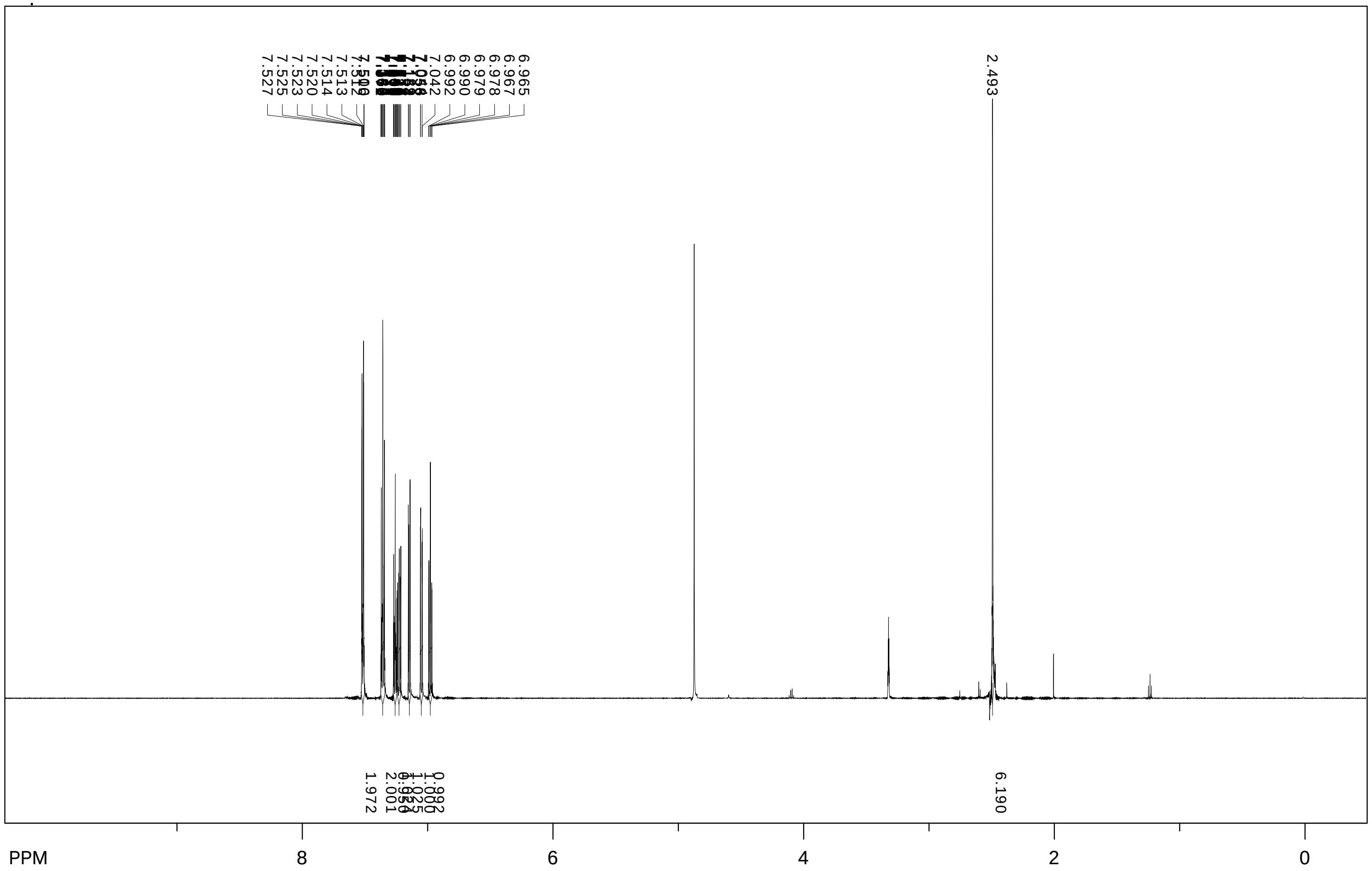

^{1}H NMR ($CDCl_3$, 300 MHz) of *N*,*N*-dimethyl-2-aminobiphenyl (aromatic region)

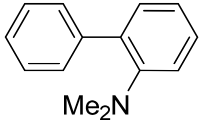

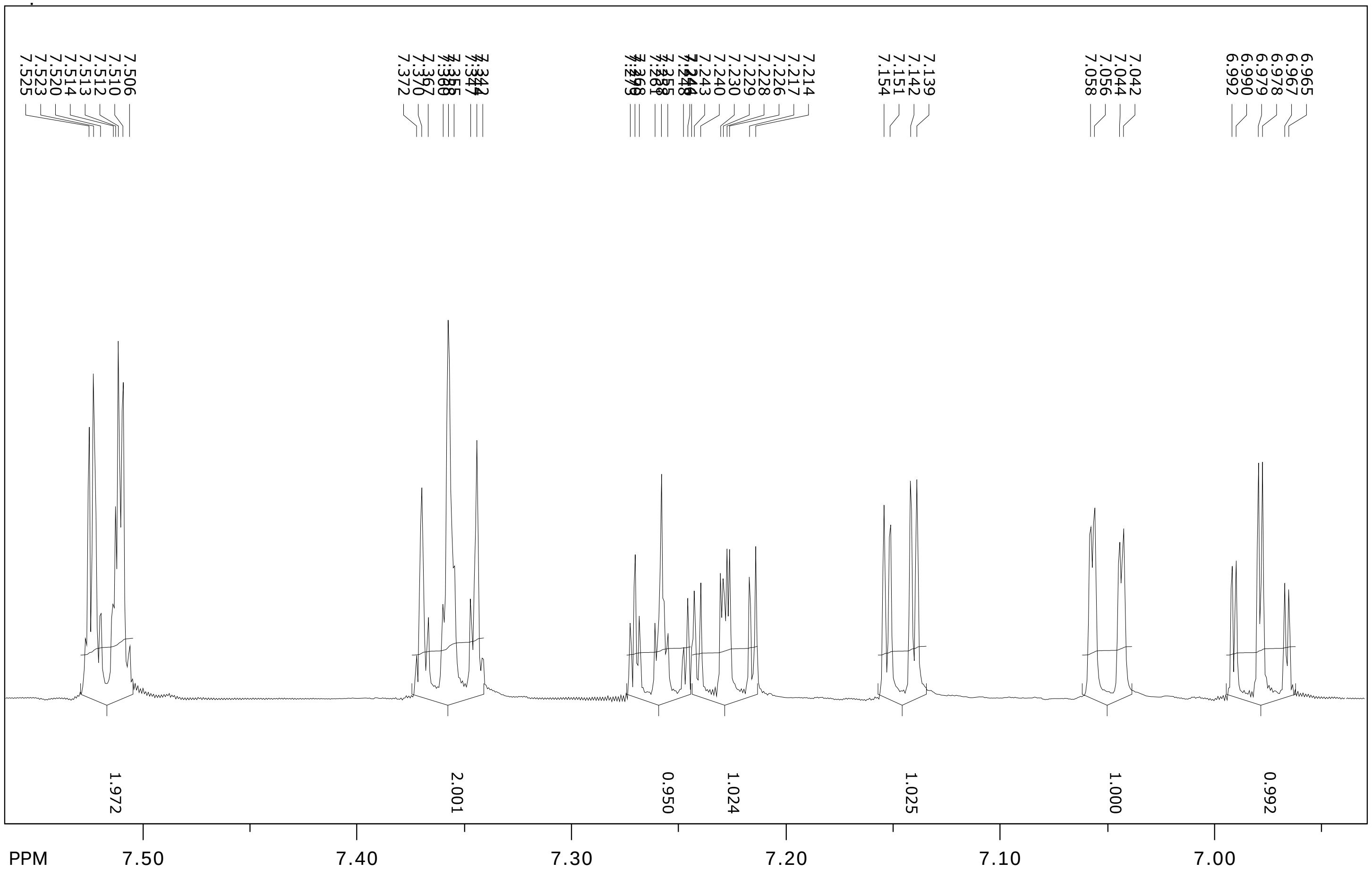

$^{13}C\ \{^{1}H\}$ NMR ($CDCl_3$, 75 MHz) of *N*,*N*-dimethyl-2-aminobiphenyl

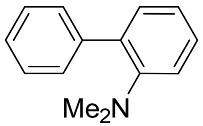

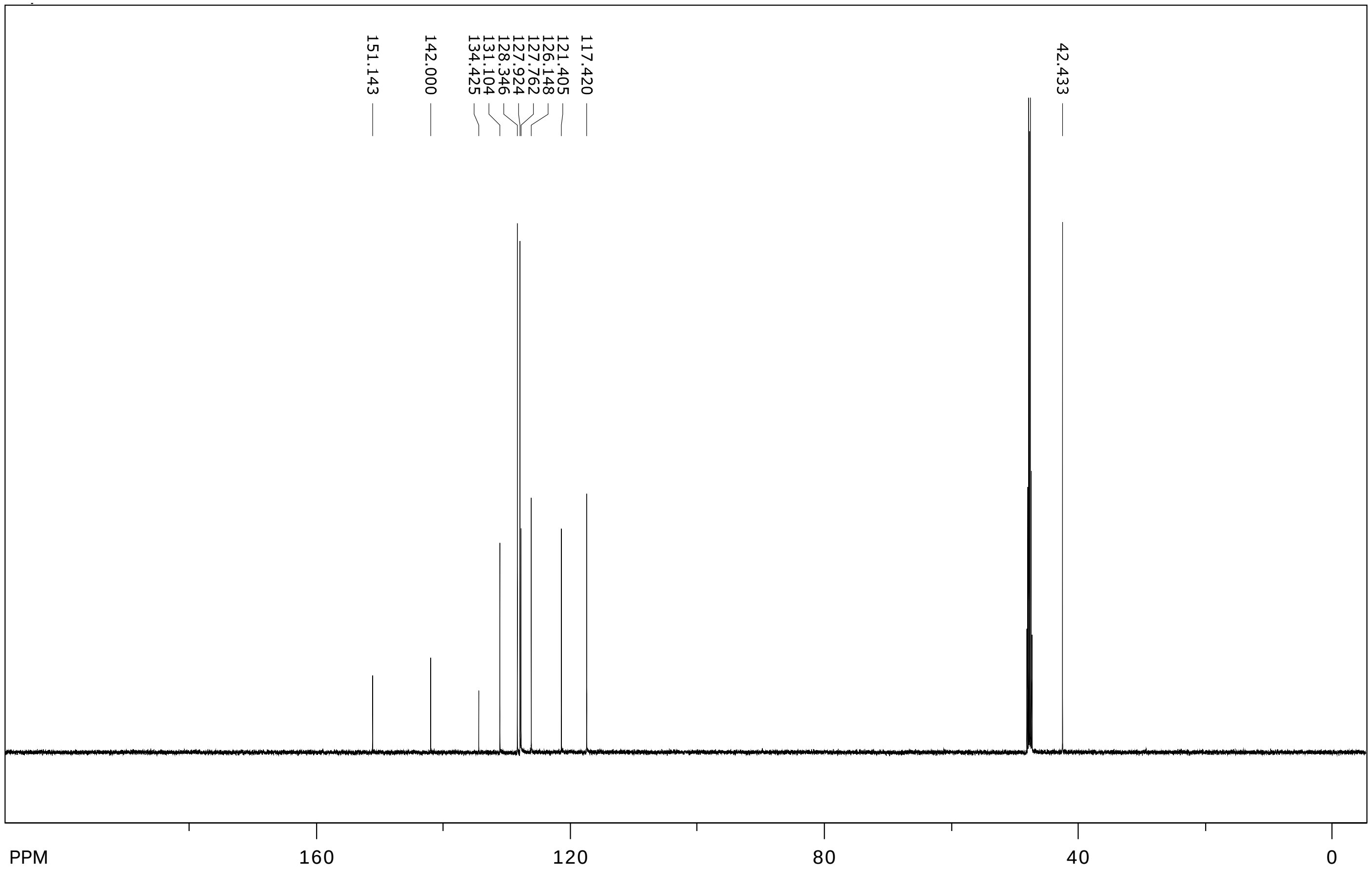

$^{13}C\ \{^{1}H\}$ NMR ($CDCl_3$, 75 MHz) of *N*,*N*-dimethyl-2-aminobiphenyl (aromatic region)

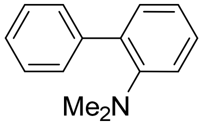

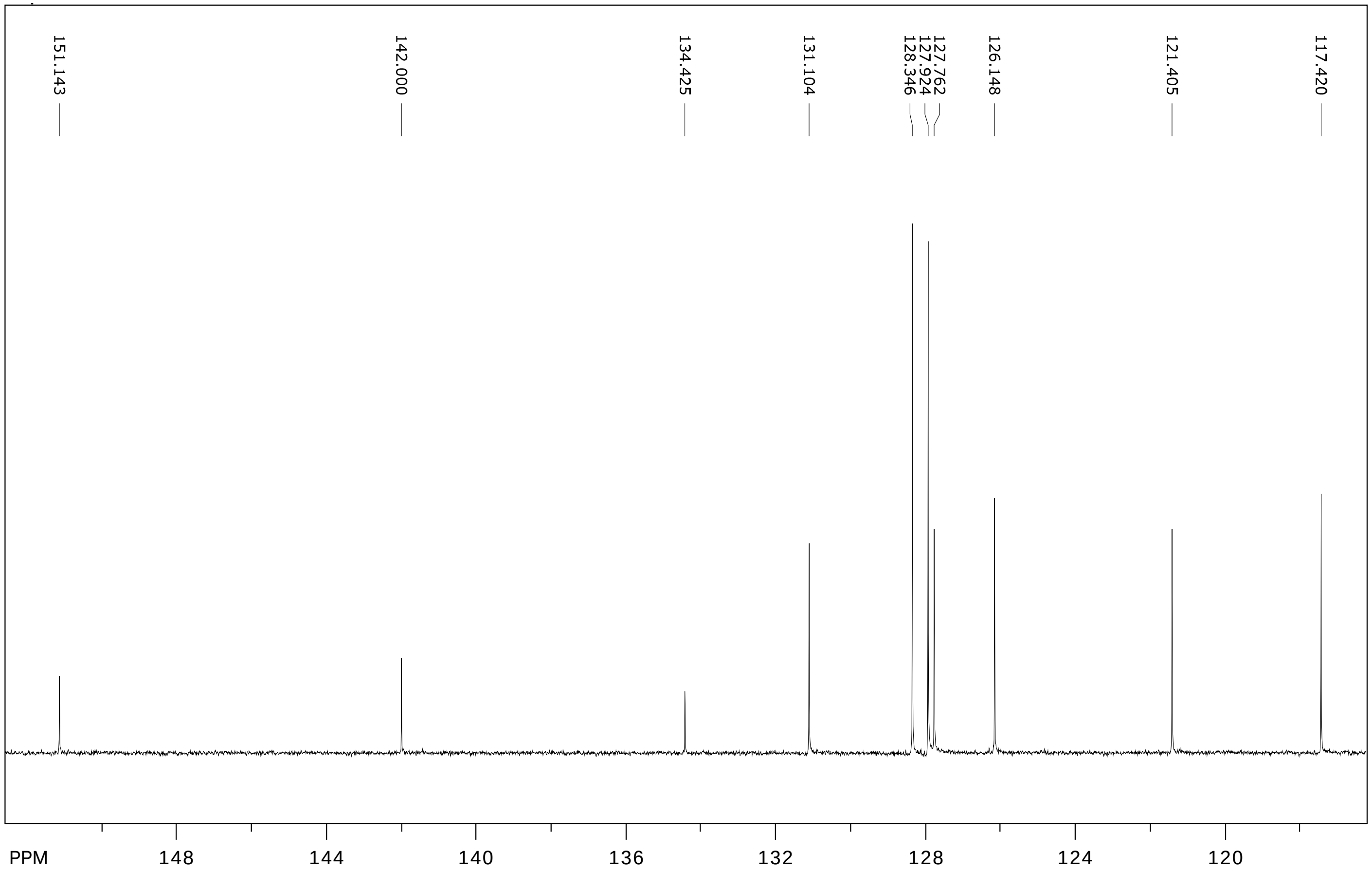

^{1}H NMR (CD_3OD, 600 MHz) of 2-amino-2'-deuteriobiphenyl (**1-D**) after 4 days of irradiation

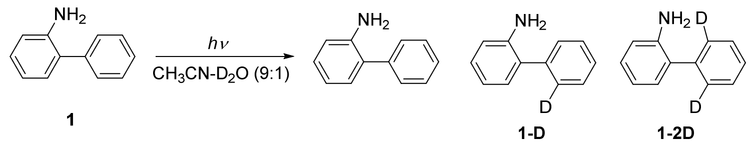

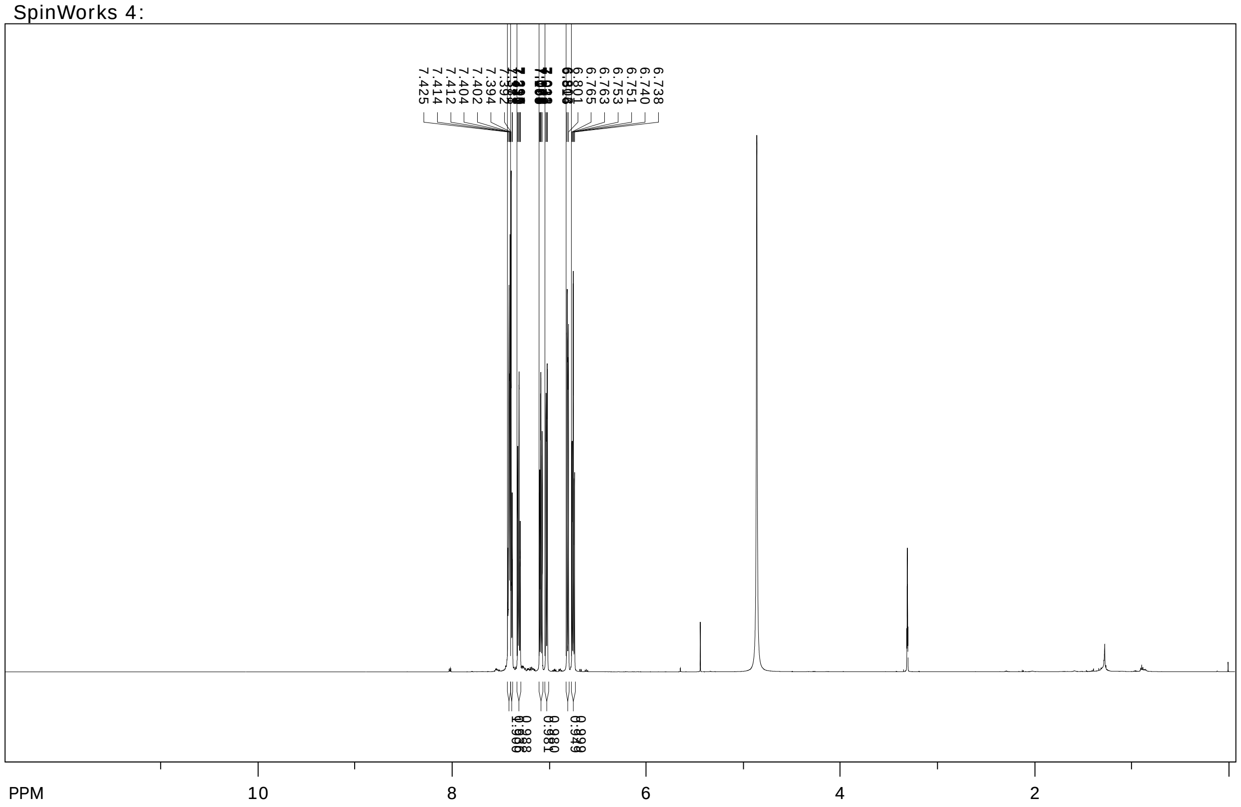

file: ...024-01-18_NB_N-480_3-4_MeOD2\1\fid expt: <zg30>
transmitter freq.: 600.173706 MHz
time domain size: 65536 points
width: 11904.76 Hz = 19.8355 ppm = 0.181652 Hz/pt
number of scans: 8

freq. of 0 ppm: 600.170012 MHz
processed size: 65536 complex points
LB: 0.300 GF: 0.0000

^{1}H NMR (CD_3OD, 600 MHz, enlarged aromatic part) of 2-amino-2’-deuteriobiphenyl (**1-D**) after 4 days of irradiation

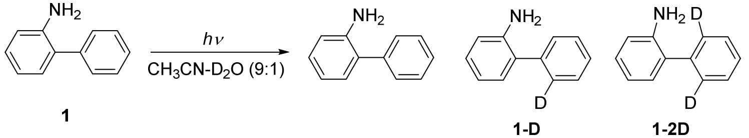

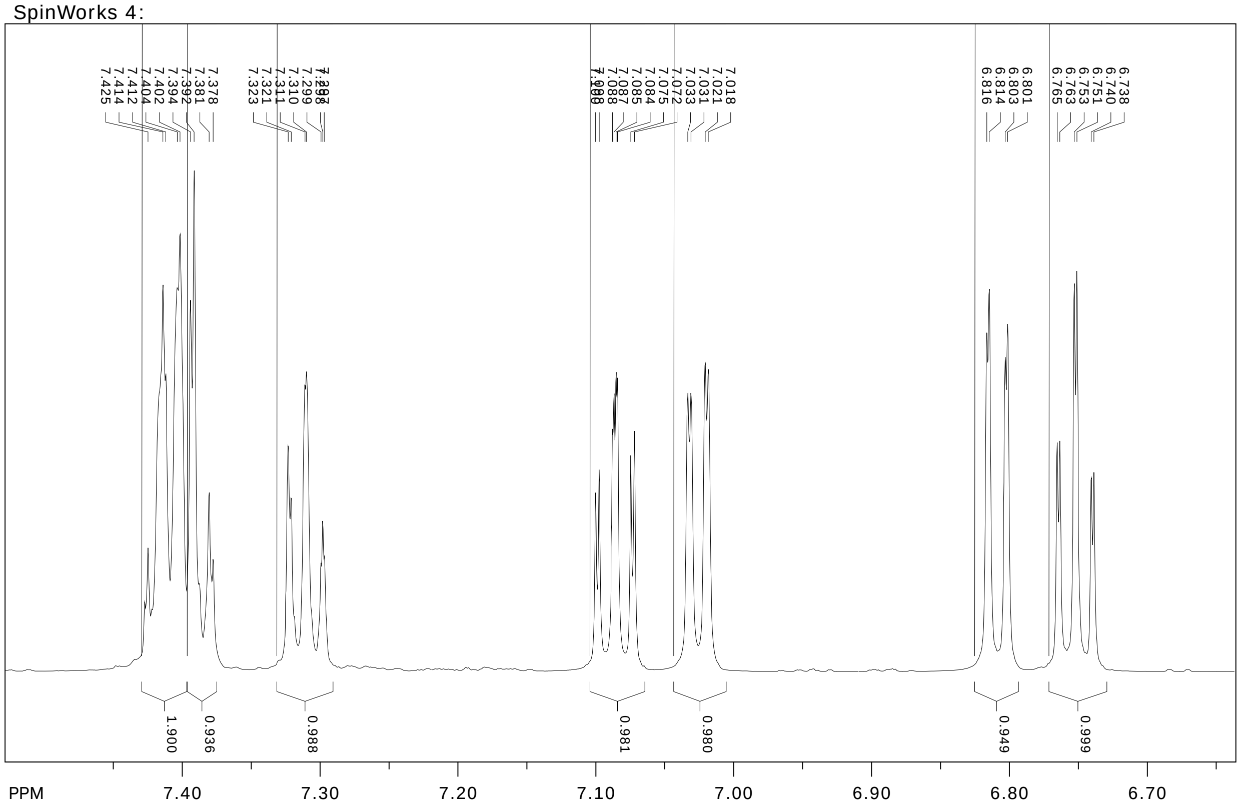

file: ...024-01-18_NB_N-480_3-4_MeOD2\1\fid expt: <zg30>
transmitter freq.: 600.173706 MHz
time domain size: 65536 points
width: 11904.76 Hz = 19.8355 ppm = 0.181652 Hz/pt
number of scans: 8

freq. of 0 ppm: 600.170012 MHz
processed size: 65536 complex points
LB: 0.300 GF: 0.0000

$^{13}C\ \{^{1}H\}$ NMR (CD_3OD, 150 MHz) of 2-amino-2’-deuteriobiphenyl (**1-D**) after 4 days of irradiation

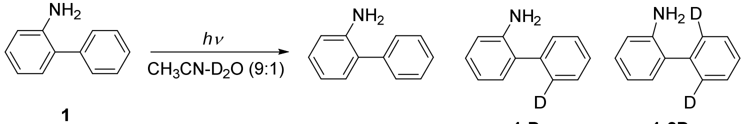

1 **1-D** **1-2D**

SpinWorks 4:

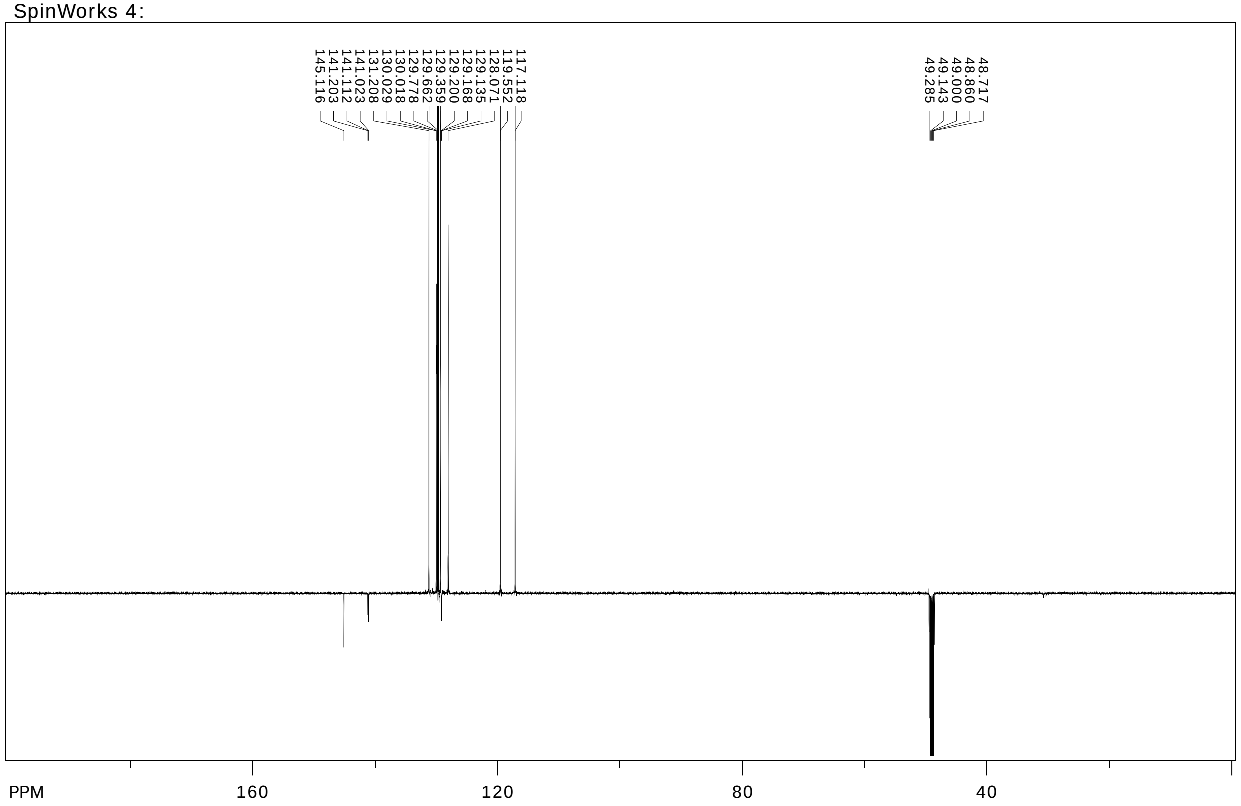

file: ...024-01-18_NB_N-480_3-4_MeOD2\2\fid expt: <deptqgpsp>
transmitter freq.: 150.927958 MHz
time domain size: 65536 points
width: 35714.29 Hz = 236.6313 ppm = 0.544957 Hz/pt
number of scans: 1706

freq. of 0 ppm: 150.912662 MHz
processed size: 32768 complex points
LB: 1.000 GF: 0.0000

$^{13}C\ \{^{1}H\}$ NMR (CD_3OD, 150 MHz, enlarged aromatic part) of 2-amino-2'-deuteriobiphenyl (**1-D**) after 4 days of irradiation

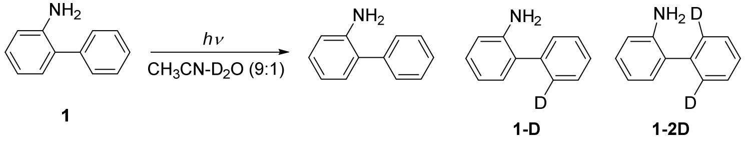

SpinWorks 4:

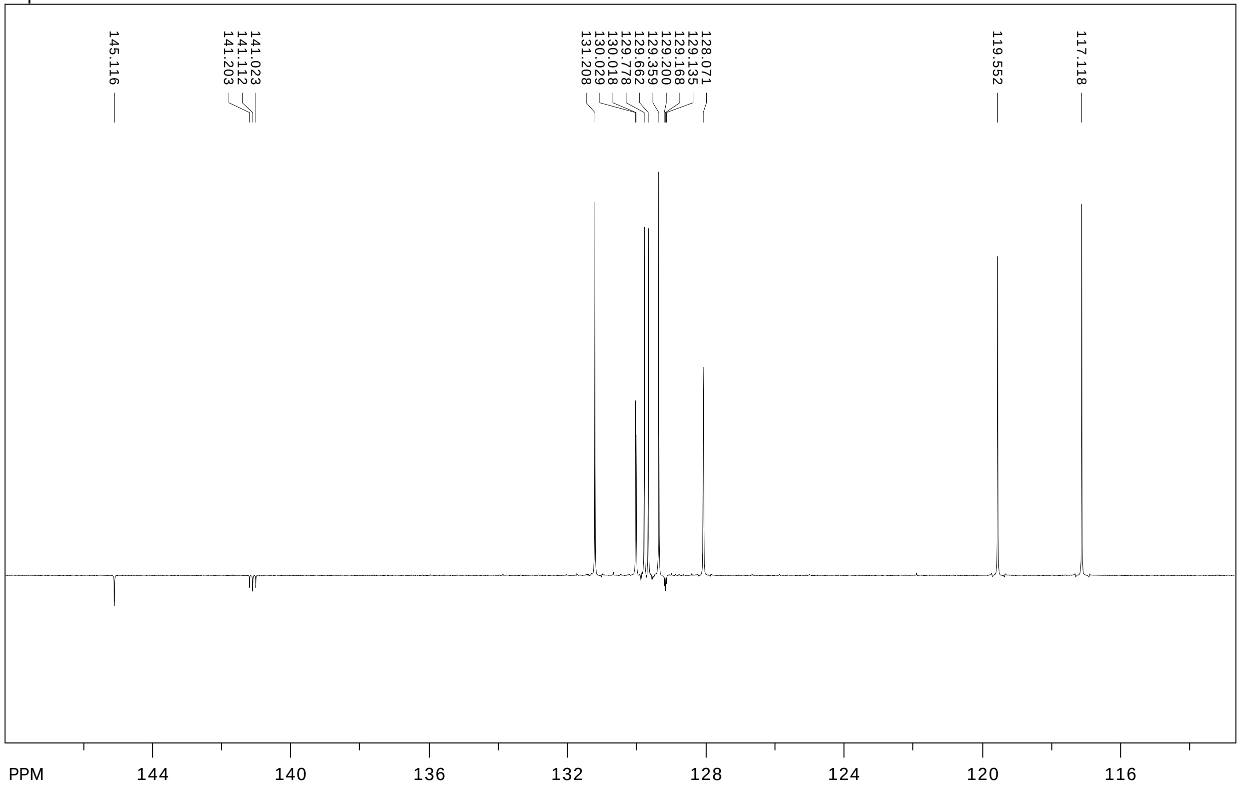

file: ...024-01-18_NB_N-480_3-4_MeOD2\2\fid expt: <deptqgpsp>
transmitter freq.: 150.927958 MHz
time domain size: 65536 points
width: 35714.29 Hz = 236.6313 ppm = 0.544957 Hz/pt
number of scans: 1706

freq. of 0 ppm: 150.912662 MHz
processed size: 32768 complex points
LB: 1.000 GF: 0.0000

MS (ESI+) of 2-amino-2'-deuteriobiphenyl (**1-D**) after 2 days of irradiation

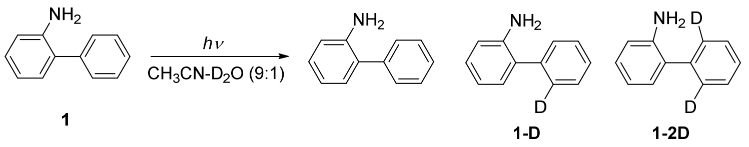

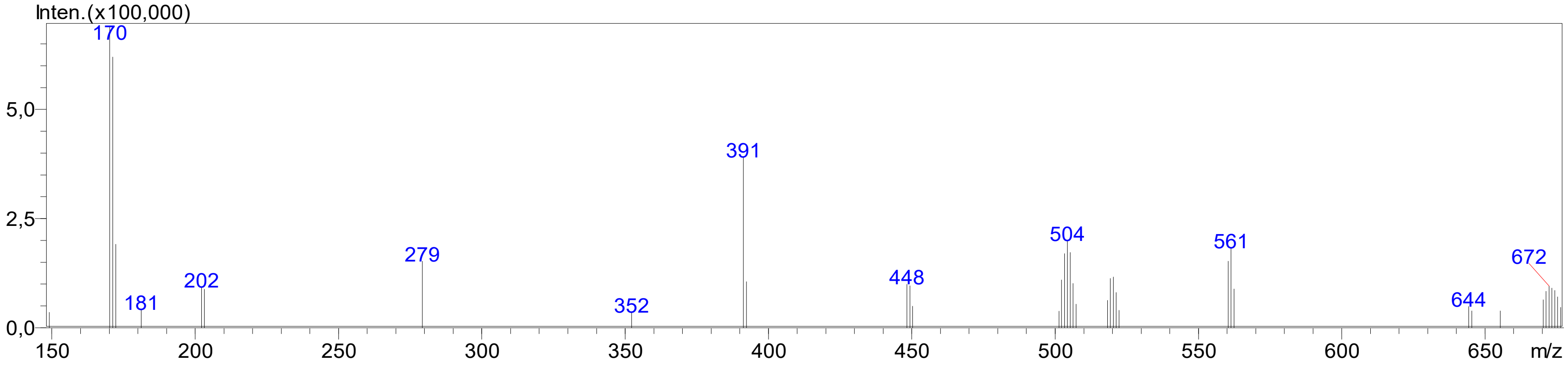

m/z	Absolute Intensity	Relative Intensity
170.2	689356	100
171.2	620070	89.95
172.25	191088	27.72

MS data indicates the presence of 50% **1**, 40% **1-D** and 10% **1-2D**

MS (ESI+) of 2-amino-2'-deuteriobiphenyl (**1-D**) after 4 days of irradiation

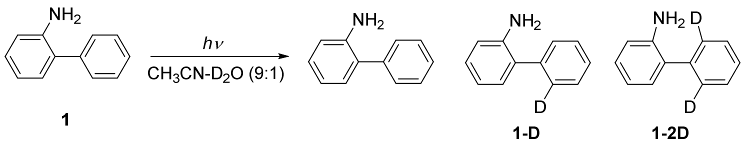

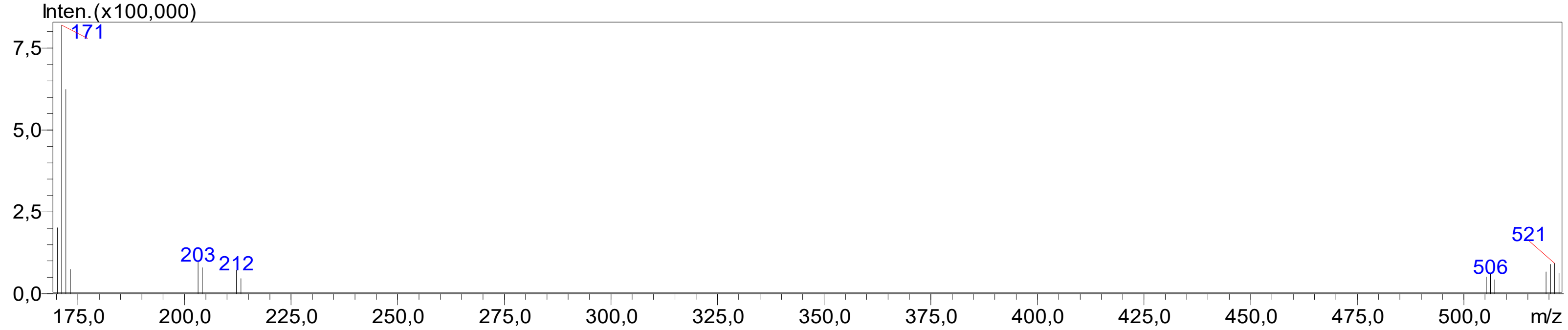

m/z	Absolute Intensity	Relative Intensity
170.2	201273	24.54
171.2	820348	100
172.2	624254	76.1
173.25	74603	9.09

MS data indicates the presence of 12% **1**, 21% **1-D**, 35% **1-2D** and 1% **1-3D**

^{1}H NMR (CD_3OD, 600 MHz) of 2-amino-2’-deuteriobiphenyl (**1-D**) after 8 days of irradiation

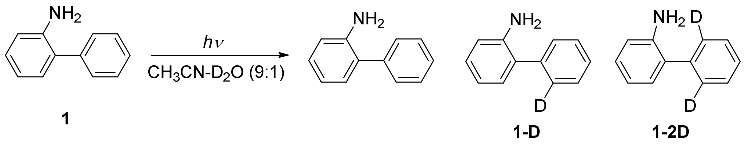

SpinWorks 4:

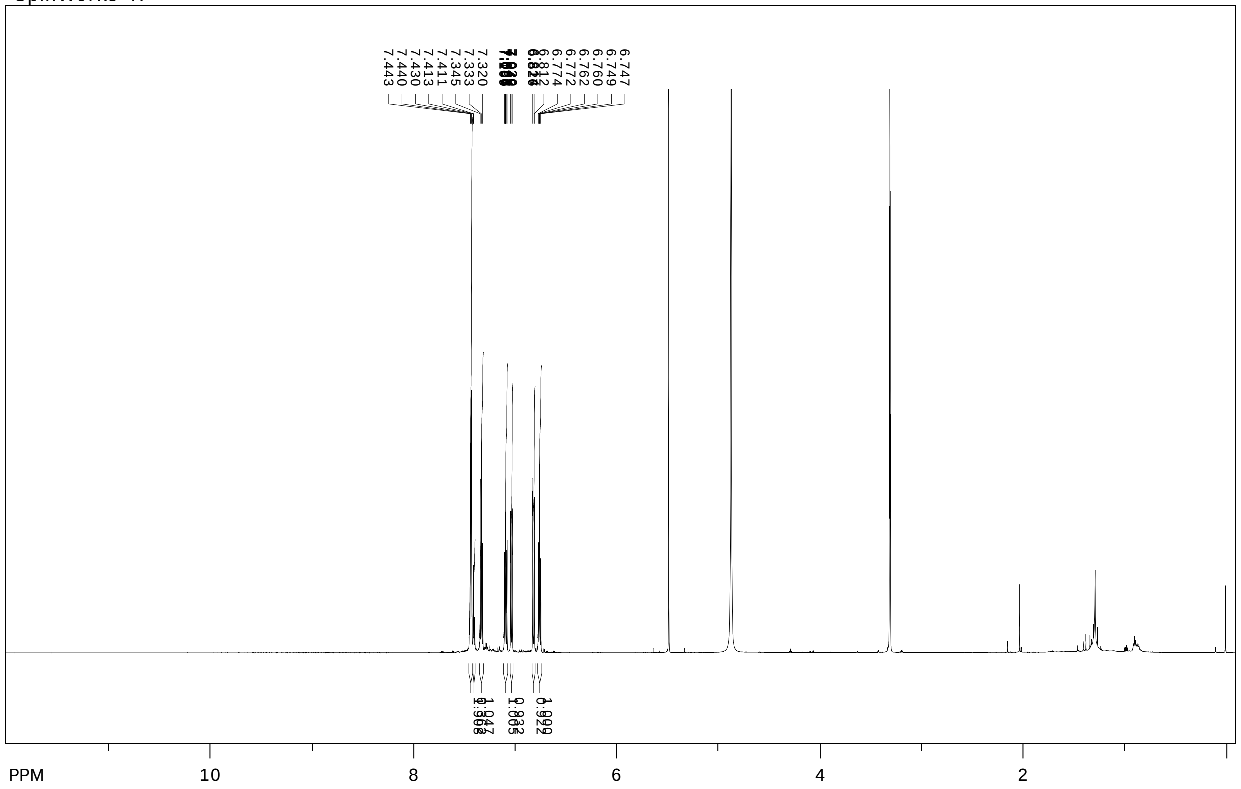

file: ...F\2024-01-23_NB_N-481-4_MeOD\1\fid expt: <zg30>
transmitter freq.: 600.173706 MHz
time domain size: 65536 points
width: 11904.76 Hz = 19.8355 ppm = 0.181652 Hz/pt
number of scans: 8

freq. of 0 ppm: 600.170012 MHz
processed size: 65536 complex points
LB: 0.300 GF: 0.0000

^{1}H NMR (CD_3OD, 600 MHz, enlarged aromatic part) of 2-amino-2'-deuteriobiphenyl (**1-D**) after 8 days of irradiation

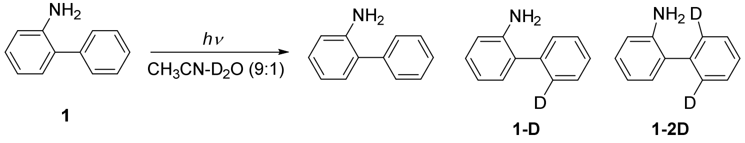

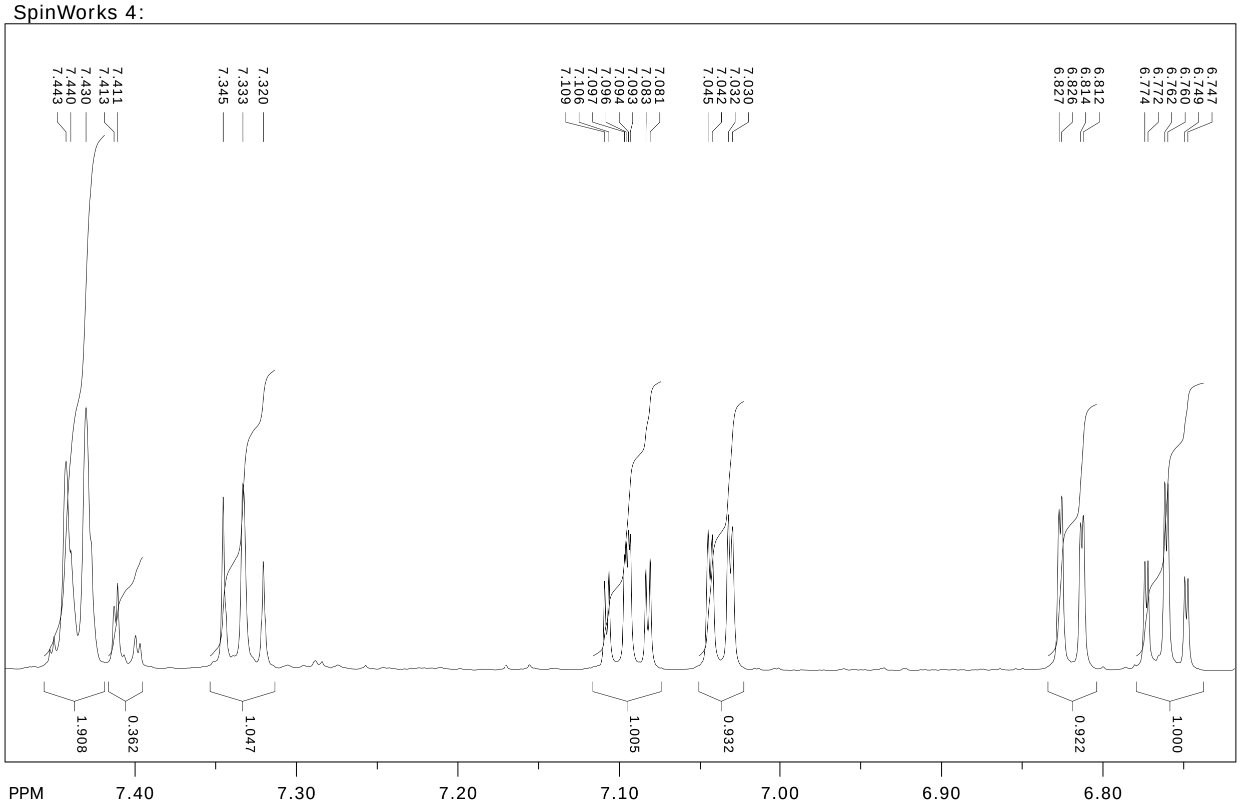

file: ...F\2024-01-23_NB_N-481-4_MeOD\1\fid expt: <zg30>
transmitter freq.: 600.173706 MHz
time domain size: 65536 points
width: 11904.76 Hz = 19.8355 ppm = 0.181652 Hz/pt
number of scans: 8

freq. of 0 ppm: 600.170012 MHz
processed size: 65536 complex points
LB: 0.300 GF: 0.0000

^{13}C {^{1}H} NMR (CD_3OD, 150 MHz) of 2-amino-2'-deuteriobiphenyl (**1-D**) after 8 days of irradiation

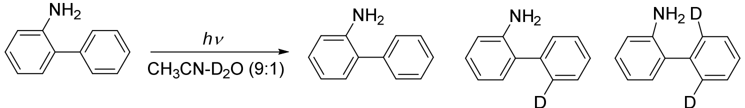

1 **1-D** **1-2D**

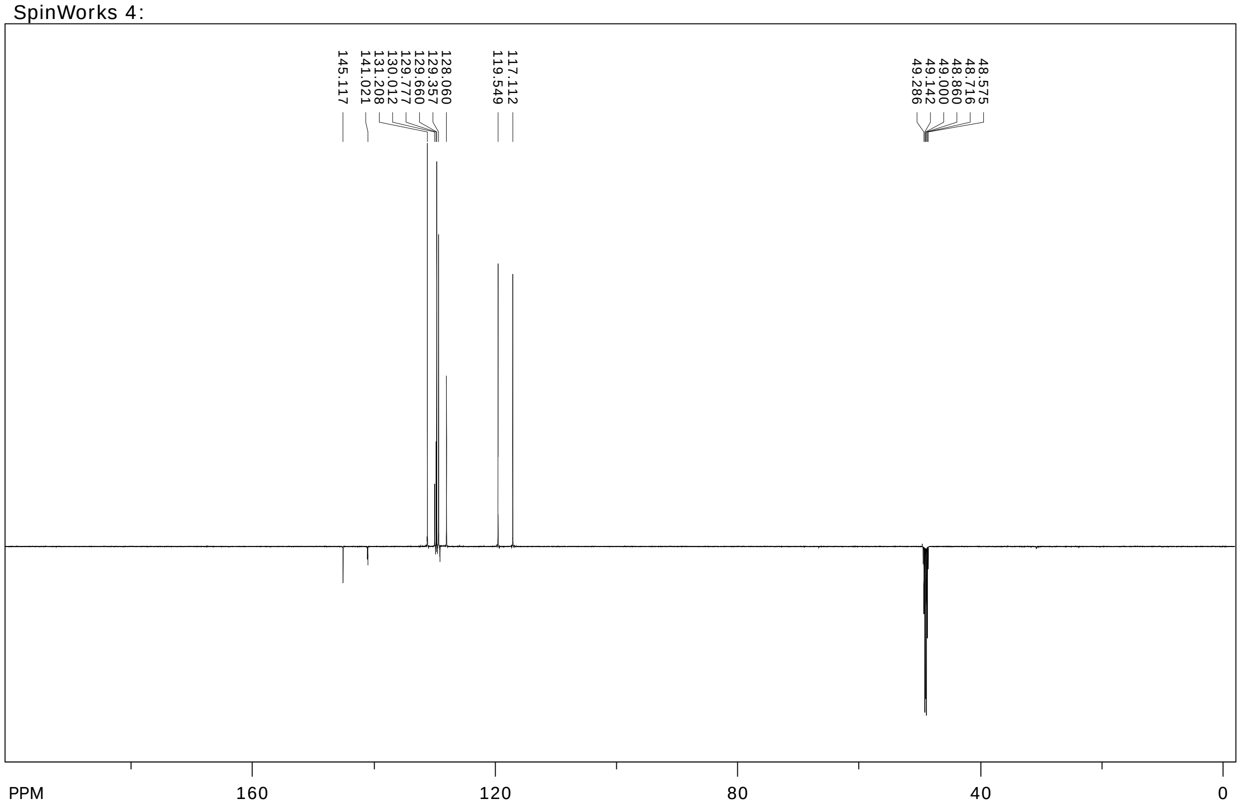

file: ...F\2024-01-23_NB_N-481-3_MeOD\2\fid expt: <deptqgpsp>
transmitter freq.: 150.927958 MHz
time domain size: 65536 points
width: 35714.29 Hz = 236.6313 ppm = 0.544957 Hz/pt
number of scans: 2560

freq. of 0 ppm: 150.912663 MHz
processed size: 32768 complex points
LB: 1.000 GF: 0.0000

^{13}C {^{1}H} NMR (CD_3OD, 150 MHz, enlarged aromatic part) of 2-amino-2'-deuteriobiphenyl (**1-D**) after 8 days of irradiation

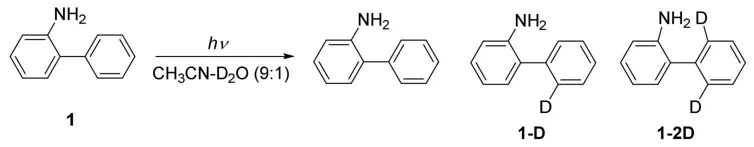

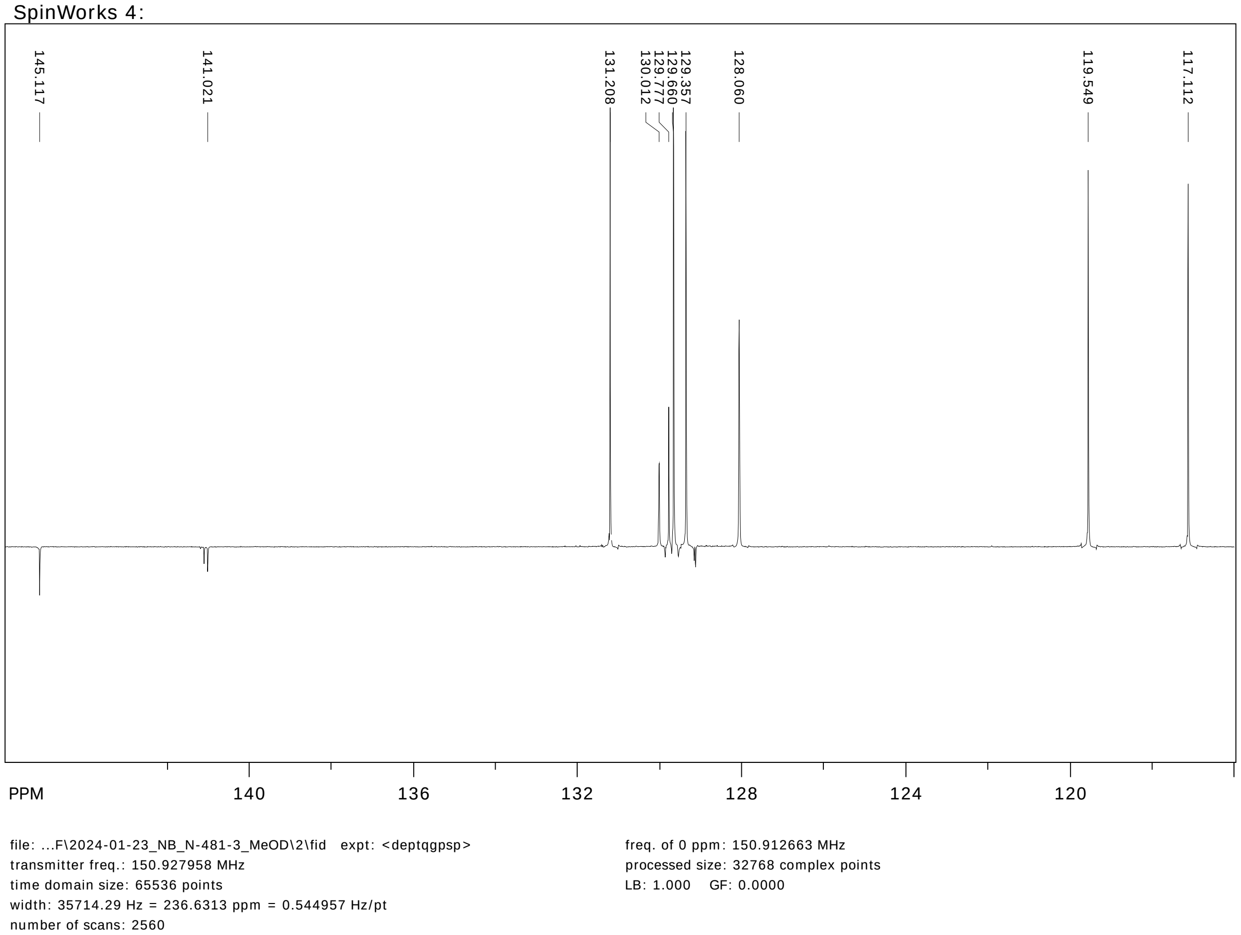

^{1}H NMR (CD_3OD, 600 MHz, enlarged aromatic part) of 2-amino-2'-deuteriobiphenyl (**1-D**) after 2 (top), 4 (middle), and 8 days (bottom) of irradiation

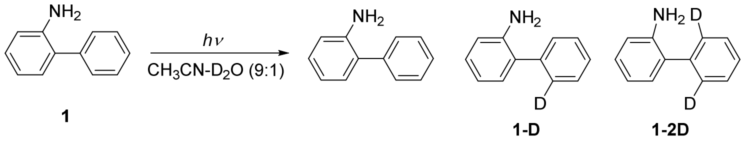

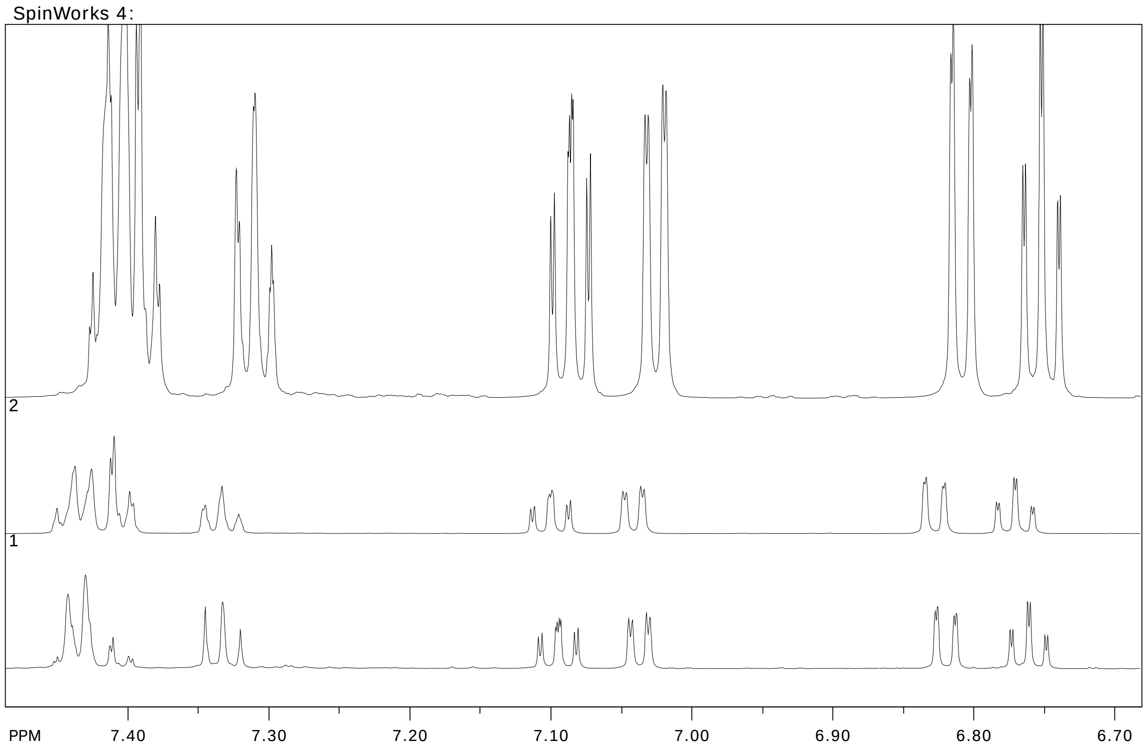

file: ...F\2024-01-23_NB_N-481-4_MeOD\1\fid expt: <zg30>
transmitter freq.: 600.173706 MHz
time domain size: 65536 points
width: 11904.76 Hz = 19.8355 ppm = 0.181652 Hz/pt
number of scans: 8

freq. of 0 ppm: 600.170012 MHz
processed size: 65536 complex points
LB: 0.300 GF: 0.0000

^{13}C {^{1}H} NMR (CD_3OD, 150 MHz, enlarged aromatic part) of 2-amino-2’-deuteriobiphenyl (**1-D**) after 4 (top) and 8 days (bottom) of irradiation

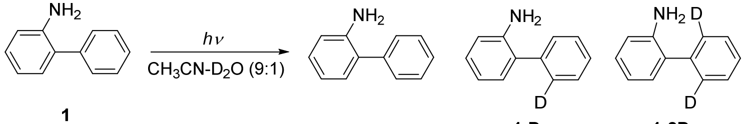

1 **1-D** **1-2D**

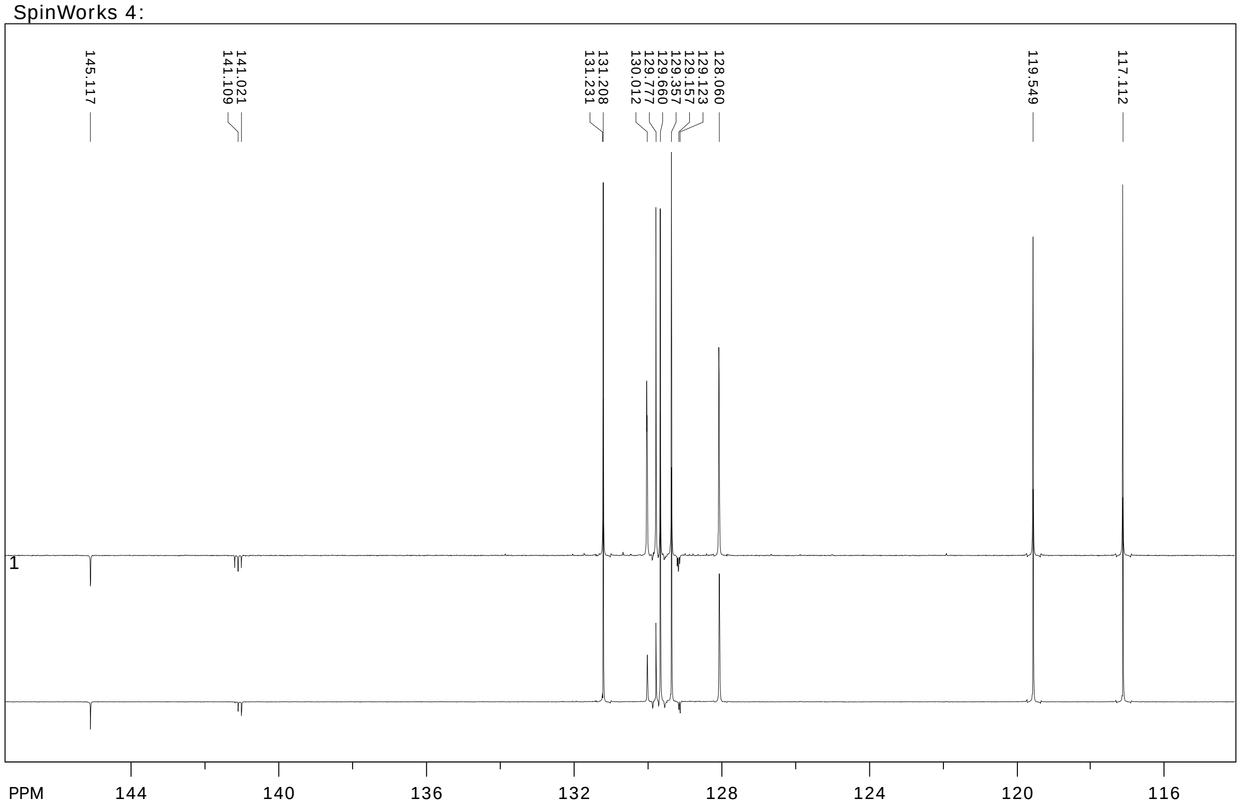

file: ...F\2024-01-23_NB_N-481-3_MeOD\2\fid expt: <deptqgpsp>
transmitter freq.: 150.927958 MHz
time domain size: 65536 points
width: 35714.29 Hz = 236.6313 ppm = 0.544957 Hz/pt
number of scans: 2560

freq. of 0 ppm: 150.912663 MHz
processed size: 32768 complex points
LB: 1.000 GF: 0.0000

MS (ESI+) of 2-amino-2'-deuteriobiphenyl (**1-D**) after 8 days of irradiation

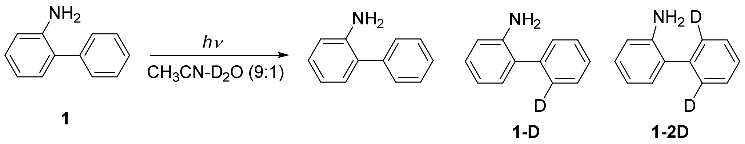

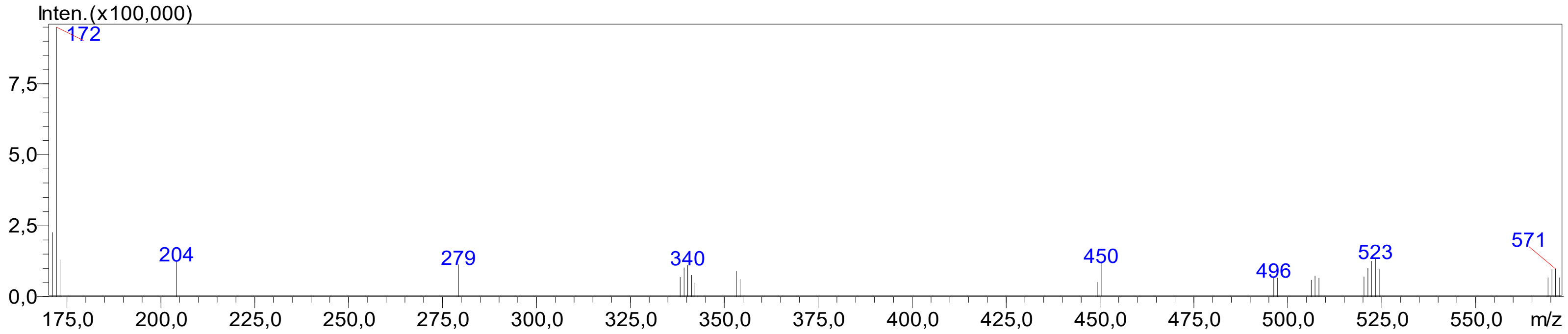

m/z	Absolute Intensity	Relative Intensity
171.2	226175	23.82
172.2	949348	100
173.2	129439	13.63

MS data indicates the presence of 0% **1**, 18% **1-D**, 79% **1-2D** and 2% **1-3D**

8. References

[1] (a) H. Grampp, M. Maeder, C. J. Meyer, A. D. Zuberbühler. Calculation of equilibrium constants from multiwavelength spectroscopic data-I Mathematical considerations. *Talanta* 1985, **32**, 95-101. (b) H. Grampp, M. Maeder, C. J. Meyer, A. D. Zuberbühler. Calculation of equilibrium constants from multiwavelength spectroscopic data--II: SPECFIT: two user-friendly programs in basic and standard FORTRAN 77. *Talanta* 1985, **32**, 257-264. (c) H. Grampp, M. Maeder, C. J. Meyer, A. D. Zuberbühler. Calculation of equilibrium constants from multiwavelength spectroscopic data-III Model-free analysis of spectrophotometric and ESR titrations. *Talanta* 1985, **32**, 1133-1139.

[2] Đ. Škalamera, V. Blažek Bregović, I. Antol, C. Bohne, N. Basarić. Hydroxymethylaniline Photocages for Carboxylic Acids and Alcohols. *J. Org. Chem.* 2017, **82**, 12554-12568.

[3] S. Goldstein, J. Rabani. The Ferrioxalate and Iodide-Iodate Actinometers in the UV Region. *J. Photochem. Photobiol.* 2008, **193**, 50-55; R. O. Rahn. Potassium Iodide as a Chemical Actinometer for 254 nm Radiation: Use of Iodate as an Electron Scavenger. *Photochem. Photobiol.* 1997, **66**, 450-455.

[4] M. Lukeman, P. Wan. A New Type of Excited-State Intramolecular Proton Transfer: Proton Transfer from Phenol OH to a Carbon Atom of an Aromatic Ring Observed for 2-Phenylphenol. *J. Am. Chem. Soc.* 2002, **124**, 9458-9464.

[5] I. Carmichael, G. L. Hug. Triplet-triplet absorption spectra of organic molecules in condensed phases. *J. Phys. Chem. Ref. Data* 1986, **15**, 1-250.

[6] A. P. Darmanyan, W. Lee, W. S. Jenks. Charge Transfer Interactions in the Generation of Singlet Oxygen $O_2(^1\Delta_g)$ by Strong Electron Donors. *J. Phys. Chem. A* 1999, **103**, 2705-2711.

[7] A. O. Lykhin, D. G. Truhlar, L. Gagliardi. Role of Triplet States in the Photodynamics of Aniline. *J. Am. Chem. Soc.* 2021, **143**, 5878-5889.

[8] G. Porter, F. J. Wright. Primary Photochemical Processes in Aromatic Molecules Part 3. Absorption Spectra of Benzyl, Anilino, Phenoxy and Related Free Radicals. *J. Chem. Soc. Faraday Trans.* 1955, **51**, 1469-1474.

[9] F. Saito, S. Tobita, H. Shizuka. Photoionization of aniline in aqueous solution and its photolysis in cyclohexane. *J. Chem. Soc. Faraday Trans.* 1996, **92**, 4177-4185.

[10] E. Leyva, M. S. Platz, B. Niu, J. Wirz. Aryiaminyl Radicals Studied by Laser Flash Photolysis of Di-*tert*-butyl Peroxide in the Presence of Arylamines. *J. Phys. Chem.* 1987, **91**, 2293-2298.

[11] J. Draženović, T. Rožić, N. Došlić, N. Basarić. Excited State Intramolecular Proton Transfer (ESIPT) from $-NH_2$ to a Carbon Atom of Naphthyl Ring. *J. Org. Chem.* 2022, **87**, 9148-9156.

[12] J. Ma, X. Zhang, N. Basarić, P. Wan, D. L. Phillips. Observation of excited state proton transfer reactions in 2-phenylphenol and 2-phenyl-1-naphthol and formation of quinone methide species. *Phys. Chem. Chem. Phys.* 2015, **17**, 9205-9211.
